# Concise Synthesis of Naphthalene-Based 14-Aza-12-Oxasteroids

**DOI:** 10.3390/molecules30020415

**Published:** 2025-01-19

**Authors:** Smriti Srivastava, Jun Luo, Daniel Whalen, Katherine N. Robertson, Amitabh Jha

**Affiliations:** 1 Department of Chemistry, Acadia University, Wolfville, NS B4P 2R6, Canada; 2 Department of Chemistry, Saint Mary’s University, Halifax, NS B3H C3C, Canada

**Keywords:** heterosteroids, 14-aza-12-oxosteroid, Sugasawa reaction, Dean–Stark condensation, double dehydration

## Abstract

A concise, transition metal-free four-step synthetic pathway has been developed for the synthesis of tetracyclic heterosteroidal compounds, 14-aza-12-oxasteroids, starting from readily available 2-naphthol analogues. After conversion of 2-naphthols to 2-naphthylamines by the Bucherer reaction, subsequent selective C-acetylation was achieved via the Sugasawa reaction and reduction of the acetyl group using borohydride, which resulted into the corresponding amino-alcohols. The naphthalene-based amino-alcohols underwent double dehydrations and double intramolecular cyclization with oxo-acids leading to one-pot formation of a C-N bond, a C-O bond and an amide bond in tandem, to generate two additional rings completing the steroidal framework. A series of 14-aza-12-oxasteroids were synthesized using our developed synthetic strategy in moderate yields, and the structure of one of the final products, 12a-Methyl-11-phenyl-11,12a-dihydro-1*H*-naphtho[2,1-d]pyrrolo[2,1-b][1,3]oxazin-3(2*H*)-one, was further confirmed by single crystal X-ray crystallography.

## 1. Introduction

**Figure 1:**
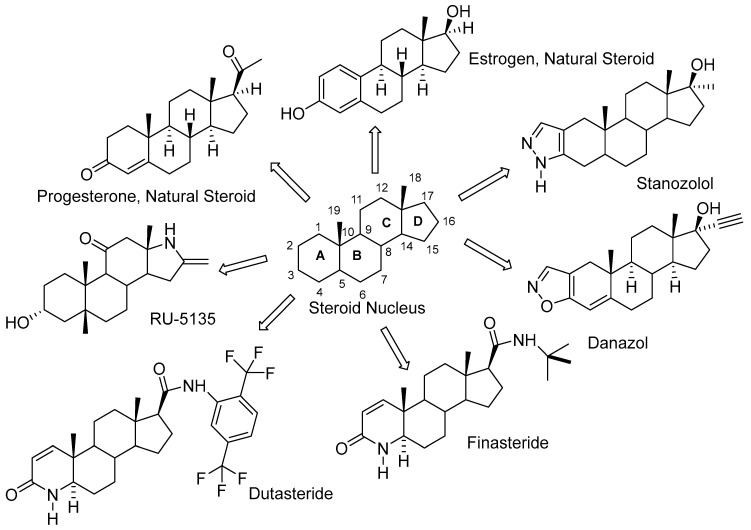
Representative natural steroids and clinically used heterosteroids.

Steroids serve as an important class of natural products by virtue of their ability to penetrate cells and bind to membranes and nuclear receptors [1,2]. Steroidal hormones are classified as glucocorticoids, mineralocorticoids, estrogen, progestogens, and androgens because of their binding to a particular receptor to express their biological response [3,4]. Estrogens (Figure 1), an aromatic ring containing steroidal hormone, bind with estrogen receptors (ER) and trigger them in promoting a variety of physiological responses such as bone maturation, neuroprotective effects, reproductive functions, modulation of blood lipid profile, and breast cell proliferation [5,6]. Progesterone is a pro-gestational steroid hormone secreted by the female reproductive system. It is associated with the menstrual cycle, pregnancy, and development of an embryo (Figure 1) [7]. Medicinal chemists are intrigued with the biological activities of various modified steroids for the drug design and development process. While retaining the steroidal framework, the structural modifications allow for modulation of the biological activities for the resulting molecules. The naturally occurring steroidal frameworks have been modified in various ways with the aim of identifying active compounds with improved efficacy and reduced toxicity [8,9]. Heterosteroids are one such modification of the natural steroids, where one or more carbons are replaced by heteroatom(s). The incorporation of heteroatoms leads to distinct chemical and biological properties, enabling a wide range of potential therapeutic applications of these compounds. The electron-rich nitrogen atoms in N-containing heterocycles are capable of easily accepting or donating protons. Due to their ability to form diverse weak interactions, they can also readily interact with enzymes and receptors in biological targets [10].

The medicinal chemistry of heterosteroids has been extensively studied [11,12,13,14,15]. A few of the heterosteroids have already culminated into successful drugs, such as stanozolol (hereditary angioedema) [16], danazol (antiestrogen) [17], RU-5135 (antagonist of GABA in the CNS) [18], finasteride (antihyperplastics) [19], and dutasteride (benign prostatic hyperplasia) [20] (Figure 1).

Although synthesis of these structurally complex molecules in a short route from readily available starting materials is a challenge in the field of organic chemistry, several aza- and oxa-steroids have been synthesized by various research groups in the past by following different synthetic protocols. Oumzil et al. [21] reported total synthesis of an 11-oxasteroid having a pyridine ring as the A ring, using an intramolecular Diels–Alder reaction. Singh and Panda [22] described an efficient approach for the synthesis of 14-azasteroids using L-proline via an intramolecular S_N_2′ cyclization reaction as a key step for the construction of the nitrogen-containing ring C. Bernath et al. [23] reported the stereospecific synthesis of 8-aza-12-oxasteroids. In continuation of our efforts in developing complex heterosteroids [24,25], we herein report a concise synthesis of 14-aza-12-oxosteroids in four simple steps. We have previously reported the synthesis of tricyclic 7-oxa-2-azatricyclo[7.4.0.02,6]trideca-1(9),10,12-trien-3-ones and their homologs, by the reaction of keto acids with methyl chloroformate and variously substituted o-aminobenzyl alcohols using triethylamine as a base in toluene at room temperature [26]; a similar type of synthesis with a different approach is reported here.

## 2. Results

**Scheme 1:**
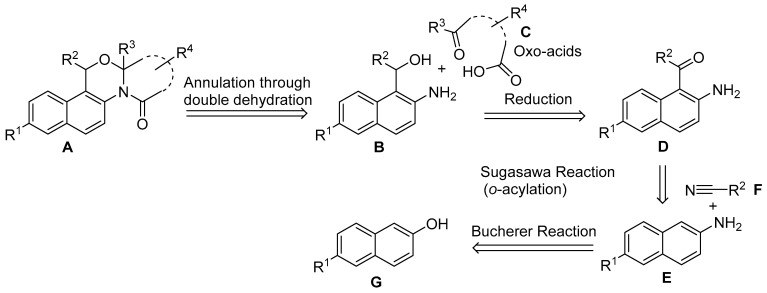
Retrosynthetic analysis for the synthesis of 14-aza-12-oxasteroids **A**.

We envisioned a synthetic strategy to obtain our designed 14-aza-12-oxasteroids with general structure **A**, the retrosynthetic analysis of which is presented in Scheme 1. Double dehydration and cyclization between **B** and the oxo-acids **C** would yield the target 14-aza-12-oxasteroids **A**. Naphthalene-based amino-alcohols **B** can be prepared by the reduction of 1-acyl-2-aminonaphthalenes **D**, which in turn can be synthesized via the Sugasawa reaction [27] of **E** with various aryl and alkyl nitriles **F**. 2-Aminonaphthalenes **E** can be obtained from commercially available 2-naphthols **G** via the Bucherer reaction [28].

**Scheme 2:**
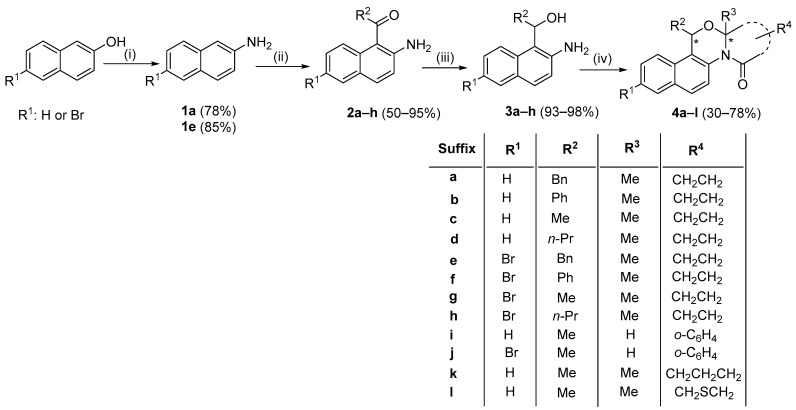
Reagents and conditions: (i) (NH_4_)_2_SO_3_ (2.0 equiv.), 28% aq. NH_3_, 100 psi, 150 °C, 5–6 h; (ii) (a) R^2^CN (6.5 equiv.), BCl_3_ (1.2 equiv.), AlCl_3_ (1.2 equiv.), N_2_, toluene, 2–4 h; (b) 1.0 M HCl (1.2 equiv. except for **2b** and **2f** where 12 M, 500.0 equiv. used), 30 min–4 h; (iii) NaBH_4_ (6.0 equiv., 12.0 equiv. for **2b** and **2f**, respectively), MeOH:THF (1:1), N_2_, 1 h; (iv) keto acids [1.5 equiv., levulinic acid/2-carboxybenzaldehyde/4-acetylbutyric acid/(2-oxopropylsulfanyl)-acetic acid], toluene, reflux, Dean–Stark trap, 2 h. “*” represents chiral centers. The yields reported are unoptimized.

Our synthetic scheme started from commercially available 2-naphthol and 6-bromo-2-naphthol (Scheme 2), which were converted into 2-napthylamine (**1a**, 78% yield, Caution: Carcinogenic!) and 6-bromo-2-napthylamine (**1e**, 85% yield), respectively, by using the Bucherer reaction [24,28]. The Friedel–Craft acylation is a common technique to attach acetyl groups on aromatic rings [29]. However, in an aniline system, coordination of the amino group with the Lewis acid results in a deactivated aromatic system. Sugasawa [27] took a unique approach to achieve *ortho-*acylation of anilines by using nitriles and two Lewis acids for the reaction. We successfully achieved *ortho*-acylation of 2-napthylamines (**1a** and **1e**) by using various nitriles (benzyl nitrile, benzonitrile, acetonitrile, and butyronitrile) by the Sugasawa protocol employing the BCl_3_ and AlCl_3_ system to obtain our desired amino-ketone compounds **2a**–**h** (Scheme 2). The reactions were performed under inert atmosphere since moisture in the air would react with the Lewis acids to form the respective hydroxides. During the work-up of this reaction, dilute acid was used to convert imine intermediates to the desired ketones, although in the case of compounds **2b** and **2f**, extensive amounts of conc. acids and significantly longer heating times were required. Requirement of the forcing conditions for imine hydrolysis was probably due to poor solubility or greater stability of these imines having conjugation with naphthalene and benzene rings. The ortho-acylated compounds **2a**–**h** were obtained in 50–95% yields after purification by column chromatography. Furthermore, these acylated 2-aminonaphthalenes **2a**–**h** were reduced to the corresponding amino-alcohol analogues **3a**–**h**, by borohydride reduction [30,31]. The crude compounds **3a**–**h** were obtained in 93–98% yields; these compounds were used for the next reaction without further purification. A greener approach not involving any reagents, with recoverable solvent and lesser reaction time, was explored to induce the double dehydration reaction between keto acids and the amino-alcohols **3a**–**h** utilizing a Dean–Stark apparatus as compared to our previous report for the synthesis of similar tricyclic compounds [26]. It was gratifying that the attempt to achieve the double dehydration and subsequent formation of three bonds in tandem between naphthalene-based amino-alcohols **3a**–**h** and various keto acids (levulinic acid/2-carboxybenzaldehyde/4-acetylbutyric acid/(2-oxopropyl sulfanyl)-acetic acid) was successful. Formation of new C-N, C-O, and amide bonds along with two cyclization reactions lead to the synthesis of our desired 14-aza-12-oxasteroid compounds **4a**–**l**, in moderate to decent yields (30–78%, Scheme 2). The structures of all the synthesized compounds **1a**, **1e**, **2a**–**h**, **3a**–**h,** and **4a**–**l** were unambiguously established based on their spectral (^1^H-, ^13^C NMR, and HRMS) data analysis. The structures of known compounds **1a** and **1e** were further confirmed on the basis of comparison of their physical and spectral data with those reported in the literature [24,28].

## 3. Discussion

**Scheme 3:**
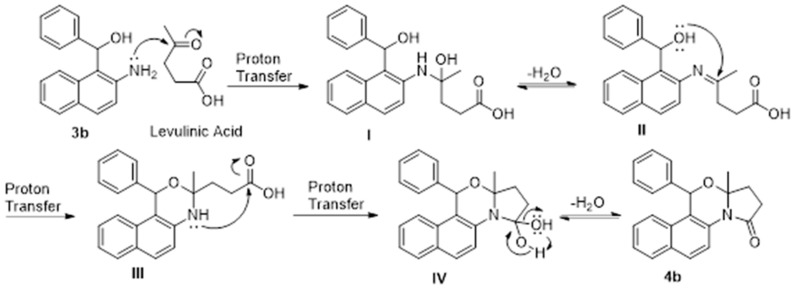
A plausible mechanism for synthesis of 14-aza-12-oxasteroids considering synthesis of **4b** as the representative example. The intermediates are labelled as **I**–**IV**.

The most plausible mechanism for the cyclization and dehydration reaction is shown in Scheme 3, taking **4b** as a representative example. The amino-alcohol **3b** contains two nucleophiles with varying nucleophilicity. The amine group is more nucleophilic than the alcohol group; therefore, it attacks the more electrophilic ketone carbonyl on levulinic acid first, eventually leading to imine formation via dehydration (**I** and **II**). Subsequent nucleophilic attack from the alcoholic hydroxy group onto the imine carbon results in intramolecular cyclization, forming the amino-acetal C-ring (**III)**. After proton transfer, the final attack from the newly formed more nucleophilic secondary amino group, on the carboxylic acid under azeotropic distillation conditions, forces the formation of the lactam ring in **4b**.

The heterosteroidal products **4a**–**l** obtained following this synthetic pathway were expected to give a mixture of up to four stereoisomeric products: the (*R*,*R*) and (*S*,*S*) enantiomeric pair and the (*R*,*S*) and (*S*,*R*) enantiomeric pair, as we did not incorporate any means for enantioselection. In most cases (except in **4i** and **4j**), there was no evidence for the formation of mixtures of diastereomers in the NMR data. The final compounds **4a–h** and **4k**–**l** showed only one set of peaks in their NMR spectra (see Supporting Information), which indicates that these molecules were synthesized as one set of enantiomeric pairs.

**Figure 2:**
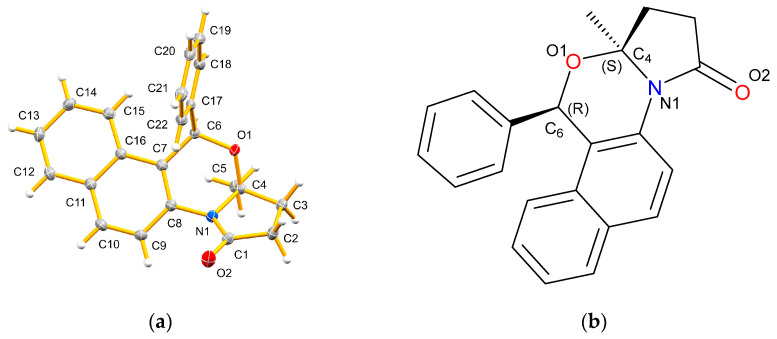
(**a**) ORTEP diagram of compound **4b** drawn in 50% thermal probability ellipsoids as a single (4*S*,6*R*) enantiomer; (**b**) chemical structure of **4b** with similar numbering and atom colouring patterns as in the X-ray crystal structure.

The diastereoselectivity observed in the formation of the target compounds indicated that among the diastereomeric pairs, the thermodynamically more stable pair of enantiomers were exclusively obtained except in the cases of **4i** and **4j** where diastereoselectivities of 6:4 and 8:2, respectively, were obtained. Inspection of the 3D structure of the target molecules reveals that the *RS/SR* enantiomeric pair (e.g., see structure of *SR*-**4b** in Figure 2) would be more thermodynamically stable as the two bulky substituents on the ring C at position C4 (Me/Ph) and C6 (the part of pyrrolidine ring) are *cis* to each other [32]. Such thermodynamic stability is expected to diminish in the case of 4i and 4j where the angular Me at position C4 is replaced by H. This explains the formation of all four stereoisomers (albeit diastereoselectively) in the cases of **4i** and **4j**.

To address the actual stereochemistry of the compounds **4a–l**, X-ray crystallographic studies on a representative compound (**4b**) were undertaken. The analysis of the X-ray crystallographic data of compound **4b** (Figure 2) showed it to crystallize in the non-centrosymmetric, polar space group *Pna*2_1_, with two molecules in the independent unit. The crystal is not a racemic mixture, containing only one of the possible enantiomeric pairs. The *R* (C6 and C28), *S* (C4 and C28) enantiomer (see SI) may have crystallized preferentially, or a crystal of this type may have been randomly selected for analysis from a mixture of crystal forms.

While there are two chiral carbon centers in each molecule, the nitrogen atom is not pyramidal and therefore not chiral. In each molecule, it is best described as being sp^2^ hybridized, with bond angles close to 120°, distorted slightly by the geometry restrictions caused by being part of two different ring systems. Neither of the rings is planar but the nitrogen atom does not lie any further out of the ring planes than the other atoms in any of the rings. The sum of the bond angles around the nitrogen center in each molecule is 354°, which is again characteristic of trigonal planar hybridization; pyramidal nitrogen centers have total angle sums of closer to 340° [33]. The Cx=O and Cx-N bond lengths (1.218(2) and 1.376(2) Å in both molecules) suggest donation of electron density from the former into the latter bond, possible from the sp^2^ hybridization at the nitrogen centers. These bond lengths are similar to those found in lactams [34], with the Cx=O bonds slightly shorter and the latter Cx-N bonds slightly longer, suggesting less donation in the molecules of **4b**. Finally, the crystal structure of compound **4b** shows that the methyl group (bonded to C4) and the phenyl group (bonded to C6) are in a trans geometry indicating that the most thermodynamically stable product has been formed. The crystallographic data of compound **4b** were deposited in the Cambridge Crystallographic Data Centre with CCDC no. 2006680. The ORTEP diagram of one of the independent molecules in compound **4b** is shown in Figure 2.

## 4. Materials and Methods

General: All the chemicals and solvents used for these syntheses were obtained from commercial sources and were used without modification, except for toluene and methanol. Toluene was dried with sodium metal overnight and distilled before reactions. Methanol was dried with molecular sieves overnight before use. (2-Oxopropylsulfanyl)-acetic acid was synthesized by following a literature procedure [35] starting from mercaptoacetic acid. TLCs were performed on pre-coated Merck silica gel 60F254 plates with the spots detected under UV light. The silica gel used in column chromatography was 230–400 mesh. All column chromatographies were performed using DCM: methanol gradient as the eluent and the TLCs were analyzed using UV chamber by comparing the Rf values of the starting material and the product. NMR spectra were recorded using a Bruker AC-300 Avance spectrometer (Bruker Corporation, Billerica, MA, USA) at 300 MHz for ^1^H NMR and 75 MHz for ^13^C NMR. The ^1^H and ^13^C NMR spectra were recorded in either CDCl_3_ or DMSO-*d_6_* solvents. ^1^H NMR spectra were reported relative to CHCl_3_ (δ: 7.26) or DMSO (δ: 2.50). ^13^C NMR spectra were reported relative to CDCl_3_ (δ: 77.16) or DMSO-d_6_ (δ: 39.52). HRMS (ESI) spectra were recorded using an Orbitrap Q-exactive analyzer (Thermo Fisher Scientific, San Jose, CA, USA; Software: Xcalibur, version 3.1). In X-ray crystallography, the crystal chosen was attached to the tip of a MicroLoop with Paratone-N oil. Measurements were made on a Bruker D8 VENTURE diffractometer equipped with a PHOTON III CMOS detector using monochromated Cu Kα radiation (λ = 1.54178 Å) from an Incoatec micro-focus sealed tube at 100 K (Bruker AXS Inc., Madison, WI, USA).

General procedure for the synthesis of 1-acyl-2-aminonaphthalenes (**2a–h**):

In a N_2_-flushed 250 mL RB flask, 2-naphthylamines (2.00 g, **1a**: 13.96 mmol; **1e**: 9.01 mmol, 1.0 equiv.) and AlCl_3_ (1.2 equiv.) were added. The flask was flushed again with N_2_. Toluene was added to the reaction mixture followed by a BCl_3_ heptane solution (1.0 M, 1.2 equiv.). The R^2^CN (acetonitrile/butyronitrile/benzonitrile/benzyl nitrile, 6.5 equiv.) was then added to the reaction mixture. The mixture was refluxed and was monitored with TLC (2% MeOH in DCM). Compounds **2a**–**b**, **2c**–**d**, **2e**–**f**, and **2g**–**h** required 3.5 h, 2.0 h, 4.0 h, and 2.5 h, respectively, for reaction completion. After cooling to rt, aq. HCl (1.0 M, 1.2 equiv., **2b** and **2f**: 12.0 M, ~500 equiv.) was added to the resultant mixture forming a yellow-orange mixture, which was heated at 80 °C for 30 min (4 h for **2b** and **2f**). During the work-up, the reaction mixture was cooled to rt, and the yellow-orange mixture was poured into ice water (ca. 50 mL). This was followed by the addition of aq. NaOH (5.0 M) until pH > 13. More ice and NaOH pellets were used as needed with **2b** and **2f**, to bring the pH > 13. The mixture was extracted with EtOAc (3 × 50 mL). The organic layers were added together and washed with brine (50 mL) and dried with anh. Na_2_SO_4_. The solvent was removed in vacuo to form the crude product. The crude product was purified by column chromatography (using DCM as eluent and slowly increasing the polarity with MeOH) to yield **2a**–**h**.

*1-(2-Aminonaphthalen-1-yl)-2-phenylethanone* (**2a**): Dark orange solid; 50% yield (1.81 g); m.p. 72–73 °C; **^1^H NMR** (CDCl_3_, 300 MHz): δ_H_ 7.85 (d, ^3^*J*_H-H_ = 9.0 Hz, 1H, Ar*H*), 7.75 (d, ^3^*J*_H-H_ = 9.0 Hz, 1H, Ar*H*), 7.70 (d, ^3^*J*_H-H_ = 9.0 Hz, 1H, Ar*H*), 7.51 (t, ^3^*J*_H-H_ = 7.5 Hz, 1H, Ar*H*), 7.39–7.28 (m, 6H, Ar*H*), 6.83 (d, ^3^*J*_H-H_ = 9.0 Hz, 1H, Ar*H*), 5.48 (brs, 2H, N*H*_2_), 4.36 (s, 2H, C*H*_2_Ph); **^13^C NMR** (CDCl_3_, 75 MHz): δ_C_ 203.6 (*C*O), 146.0, 135.6, 133.4, 129.4, 128.7, 128.4, 127.4, 126.6, 124.1, 122.6, 122.6, 119.2, 115.4 (Ar*Cs*), 50.0 (*C*H_2_Ph); **HRMS (ESI^+^)** MeOH/CHCl_3_, *m/z* (RI): 262.1227 ([M + H]^+^, 100%). [M + H]^+^ calcd. for C_18_H_15_NO, 262.1226.
*(2-Aminonaphthalen-1-yl)(phenyl)methanone* (**2b**): Yellow crystals; 60% yield (2.06 g); m.p. 166–167 °C; **^1^H NMR** (DMSO-d_6_, 300 MHz): δ_H_ 7.78 (d, ^3^*J*_H-H_ = 9.0 Hz, 1H, Ar*H*), 7.71 (d, ^3^*J*_H-H_ = 6.0 Hz, 1H, Ar*H*), 7.66 (d, ^3^*J*_H-H_ = 6.0 Hz, 2H, Ar*H*), 7.59 (t, ^3^*J*_H-H_ = 6.0 Hz, 1H, Ar*H*), 7.46 (t, ^3^*J*_H-H_ = 7.5 Hz, 2H, Ar*H*), 7.17–7.06 (m, 4H, Ar*H*), 5.93 (brs, 2H, N*H*_2_); **^13^C NMR** (DMSO-d_6_, 75 MHz): δ_C_ 197.8 (*C*O), 145.7, 138.6, 132.8, 132.2, 131.4, 129.0, 128.5, 128.0, 126.3, 125.9, 123.3, 121.2, 118.9, 112.4 (Ar*Cs*); **HRMS (ESI^+^)** MeOH/CHCl_3_, *m/z* (RI): 248.1070 ([M + H]^+^, 100%). [M + H] calcd. for C_17_H_13_NO, 248.1070.
*1-Acetyl-2-aminonaphthalene* (**2c**): Orange solid; 63% yield (1.62 g); m.p. 107–108 °C; **^1^H NMR** (CDCl_3_, 300 MHz): δ_H_ 7.82 (d, ^3^*J*_H-H_ = 9.0 Hz, 1H, Ar*H*), 7.68 (d, ^3^*J*_H-H_ = 6.0 Hz, 1H, Ar*H*), 7.65 (d, ^3^*J*_H-H_ = 6.0 Hz, 1H, Ar*H*), 7.46 (t, ^3^*J*_H-H_ = 9.0 Hz, 1H, Ar*H*), 7.27 (t, ^3^*J*_H-H_ = 9.0 Hz, 1H, Ar*H*), 6.85 (d, ^3^*J*_H-H_ = 9.0 Hz, 1H, Ar*H*), 5.80 (brs, 2H, N*H*_2_), 2.70 (s, 3H, C*H*_3_); **^13^C NMR** (CDCl_3_, 75 MHz): δ_C_ 202.9 (*C*O), 146.4, 133.8, 132.6, 128.7, 127.7, 127.4, 124.4, 122.6, 119.5, 115.2 (Ar*Cs*), 32.3 (*C*H_3_); **HRMS (ESI^+^)** MeOH/CHCl_3_, *m*/*z* (RI): 186.2337 ([M + H]^+^, 100%). [M + H] calcd. for C_12_H_11_NO, 186.2335.
*1-(2-Aminonaphalen-1-yl)butan-1-one* (**2d**): Orange oil; 50% yield (1.33 g); **^1^H NMR** (CDCl_3_, 300 MHz): δ_H_ 7.74–7.68 (m, 2H, Ar*H*), 7.66 (d, ^3^*J*_H-H_ = 9.0 Hz, 1H, Ar*H*), 7.46 (t, ^3^*J*_H-H_ = 9.0 Hz, 1H, Ar*H*), 7.28 (t, ^3^*J*_H-H_ = 7.5 Hz, 1H, Ar*H*), 6.87 (d, ^3^*J*_H-H_ = 9.0 Hz, 1H, Ar*H*), 5.17 (brs, 2H, N*H*_2_), 2.99 (t, ^3^*J*_H-H_ = 7.5 Hz, 2H, C*H*_2_CH_2_CH_3_), 1.86 (sx, ^3^*J*_H-H_ = 7.8 Hz, 2H, CH_2_C*H*_2_CH_3_), 0.97 (t, ^3^*J*_H-H_ = 6.0 Hz, 3H, CH_2_CH_2_C*H*_3_); **^13^C NMR** (CDCl_3_, 75 MHz): δ_C_ 207.7 (*C*O), 144.8, 132.8, 131.2, 128.6, 127.6, 127.2, 124.0, 122.5, 119.6, 116.4 (ArCs), 46.2, 19.3, 13.8 (*n*-Pr); **HRMS (ESI^+^)** MeOH/CHCl_3_, *m*/*z* (RI): 214.1231 ([M + H]^+^, 100%). [M + H]^+^ calcd. for C_14_H_15_NO, 214.1226.
*1-(2-Amino-6-bromonaphthalen-1-yl)-2-phenylethanone* (**2e**): Dark purple solid; 75% yield (2.29 g); m.p. 101–102 °C; **^1^H NMR** (CDCl_3_, 300 MHz): δ_H_ 7.84 (s, 1H, Ar*H*), 7.65 (d, ^3^*J*_H-H_ = 9.0 Hz, 1H, Ar*H*), 7.55 (m, 2H, Ar*H*), 7.34–7.26 (m, 5H, Ar*H*), 6.83 (d, ^3^*J*_H-H_ = 12.0 Hz, 1H, Ar*H*), 5.46 (brs, 2H, N*H*_2_), 4.26 (s, 2H, C*H*_2_Ph); **^13^C NMR** (CDCl_3_, 75 MHz): δ_C_ 203.2 (*C*O), 146.0, 135.3, 132.3, 130.6, 130.4, 129.3, 128.7, 128.5, 126.8, 125.7, 120.3, 115.9 (Ar*Cs*), 50.1 (*CH_2_*Ph); **HRMS (ESI^+^)** MeOH/CHCl_3_, *m/z* (RI): 340.0335 ([M + H]^+^, 98%). [M + H]^+^ calcd. for C_18_H_14_BrNO, 340.0332.
*(2-Amino-6-bromonaphthalen-1-yl)(phenyl)methanone* (**2f**): Yellow crystals; 65% yield (1.90 g); m.p. 120–121 °C; **^1^H NMR** (DMSO-d_6_, 300 MHz): δ_H_ 7.97 (d, ^4^*J*_H-H_ = 1.5 Hz, 1H, Ar*H*), 7.77 (d, ^3^*J*_H-H_ = 12.0 Hz, 1H, Ar*H*), 7.64 (d, ^3^*J*_H-H_ = 6.0 Hz, 2H, Ar*H*), 7.60 (d, ^3^*J*_H-H_ = 6.0 Hz, 1H, Ar*H*), 7.46 (t, ^3^*J*_H-H_ = 7.5 Hz, 2H, Ar*H*), 7.27 (d, ^3^*J*_H-H_ = 6.0 Hz, 1H, Ar*H*), 7.16 (d, ^3^*J*_H-H_ = 9.0 Hz, 1H, Ar*H*), 7.00 (d, ^3^*J*_H-H_ = 9.0 Hz, 1H, Ar*H*), 6.03 (s, 2H, N*H*_2_); **^13^C NMR** (DMSO-d_6_, 75 MHz): δ_C_ 197.6 (*C*O), 146.4, 138.6, 133.3, 131.1, 130.9, 129.9, 129.3, 128.9, 127.4, 125.6, 120.4, 113.9, 112.3 (Ar*Cs*); **HRMS (ESI^+^)** MeOH/CHCl_3_, *m/z* (RI): 326.0184 ([M + H]^+^, 100%). [M + H]^+^ calcd. for C_17_H_12_BrNO, 326.0175.
*1-Acetyl-2-amino-6-bromonaphthalene* (**2g**): Orange solid; 95% yield (2.25 g); m.p. 135–137 °C. **^1^H NMR** (CDCl_3_, 300 MHz): δ_H_ 7.80 (s, 1H, Ar*H*), 7.68 (d, ^3^*J*_H-H_ = 9.0 Hz, 1H, Ar*H*), 7.55 (d, ^3^*J*_H-H_ = 9.0 Hz, 2H, Ar*H*), 6.84 (d, ^3^*J*_H-H_ = 9.0 Hz, 1H, Ar*H*), 5.94 (brs, 2H, N*H*_2_), 2.66 (s, 3H, C*H*_3_); **^13^C NMR** (CDCl_3_, 75 MHz): δ_C_ 202.3 (*C*O), 147.0, 132.7, 131.2, 130.6, 130.4, 130.0, 128.8, 128.2, 125.9, 120.6 (Ar*Cs*), 32.2 (*C*H_3_); **HRMS (ESI^+^)** MeOH/CHCl_3_, *m/z* (RI): 265.1291 ([M + H]^+^, 100%). [M + H]^+^ calcd. for C_12_H_11_BrNO, 265.1295.
*1-(2-Amino-6-bromonaphalen-1-yl)butan-1-one* (**2h**): Brown solid; 60% yield (1.57 g); m.p. 42–43 °C; **^1^H NMR** (CDCl_3_, 300 MHz): δ_H_ 7.81 (s, 1H, Ar*H*), 7.59–7.47 (m, 3H, Ar*H*), 6.86 (d, ^3^*J*_H-H_ = 9.0 Hz, 1H, Ar*H*), 5.02 (brs, 2H, N*H*_2_), 2.92 (t, ^3^*J*_H-H_ = 7.5 Hz, 2H, C*H*_2_CH_2_CH_3_), 1.79 (dd, ^3^*J*_H-H_ = 15.0 Hz, 9.0 Hz, 2H, CH_2_C*H*_2_CH_3_), 0.95 (t, ^3^*J*_H-H_ = 7.5 Hz, 3H, CH_2_CH_2_C*H*_3_); **^13^C NMR** (CDCl_3_, 75 MHz): δ_C_ 207.1 (*C*O), 145.1, 131.8, 130.7, 130.5, 130.2, 128.7, 125.6, 120.3, 116.1, 115.7 (Ar*Cs*), 46.2, 19.3, 13.8 (*n*-Pr); **HRMS (ESI^+^)** MeOH/CHCl_3_, *m*/*z* (RI): 292.0337 ([M + H]^+^, 100%). [M + H]^+^ calcd. for C_14_H_14_BrNO, 292.0332.

General procedure for the synthesis of 1-(1-hydroxyalkyl)-2-aminonaphthalenes (**3a**–**h**):

In a 50 mL RB flask, **2a**–**h** (500 mg, 1.0 equiv.) was dissolved in a 1:1 mixture of methanol and THF (10 mL). The reaction flask was flushed with N_2_ gas and cooled to 0 °C in an ice bath. While stirring, NaBH_4_ powder (6.0 equiv., except **2b** and **2f**: 12.0 equiv.) was added in small batches. The reaction was stirred in an RB flask with a N_2_ balloon for 5 min. in the ice bath, and further stirred at rt until no starting material was observed on the TLC (ca. 1 h). The solvent was removed in vacuo leaving a solid. Distilled water (ca. 5 mL) was added, and the products were extracted with EtOAc (2 × 25 mL) and washed with distilled water (2 × 10 mL). The organic layer was dried with anh. Na_2_SO_4_ and the solvent was removed in vacuo yielding the products in pure form.

*1-(2-Amimonaphthalen-1-yl)-2-phenylethanol* (**3a**): Orange solid; 93% yield (467 mg); m.p. 129–131 °C; **^1^H NMR** (CDCl_3_, 300 MHz): δ_H_ 7.82 (d, ^3^*J*_H-H_ = 6.0 Hz, 1H, Ar*H*), 7.73 (d, ^3^*J*_H-H_ = 6.0 Hz, 1H, Ar*H*), 7.61 (d, ^3^*J*_H-H_ = 9.0 Hz, 1H, Ar*H*), 7.44 (t, ^3^*J*_H-H_ = 7.5 Hz, 1H, Ar*H*), 7.39–7.25 (m, 6H, Ar*H*), 6.89 (d, ^3^*J*_H-H_ = 9.0, 1H, Ar*H*), 5.85 (dd, ^3^*J*_H-H_ = 9.0 Hz, 3.0 Hz, 1H, Ar*H*), 3.81 (brs, 3H, N*H*_2_ and O*H*), 3.39 (dd, ^2^*J*_H-H_ = 15.0 Hz, ^3^*J*_H-H_ *=* 9.0 Hz, 1H, C*H*_2_Ph), 3.12 (dd, ^2^*J*_H-H_ = 12.0 Hz, ^3^*J*_H-H_ *=* 3.0 Hz, 1H, C*H*_2_Ph); **^13^C NMR** (CDCl_3_, 75 MHz): δ_C_ 143.1, 138.6, 131.8, 129.5, 129.1, 128.8, 128.5, 128.1, 126.6, 126.6, 121.8, 120.6, 120.3, 115.9 (Ar*Cs*), 72.0 (*C*HOH), 40.7 (*CH_2_*Ph); **HRMS (ESI^+^)** MeOH/CHCl_3_, *m*/*z* (RI): 264.1383 ([M + H]^+^, 26%). [M + H]^+^ calcd. for C_18_H_17_NO, 264.1383.
*(2-Aminonaphthalen-1-yl)(phenyl)methanol* (**3b**): Pale orange solid; 94% yield (474 mg); m.p. 131–133 °C; **^1^H NMR** (DMSO-d_6_, 300 MHz): δ_H_ 7.97 (d, ^3^*J*_H-H_ = 9.0 Hz, 1H, Ar*H*), 7.67 (d, ^3^*J*_H-H_ = 9.0 Hz, 1H, Ar*H*), 7.59 (d, ^3^*J*_H-H_ = 9.0 Hz, 1H, Ar*H*), 7.38 (d, ^3^*J*_H-H_ = 6.0 Hz, 2H, Ar*H*), 7.31–7.23 (m, 3H, Ar*H*), 7.19–7.09 (m, 2H, Ar*H*), 7.02 (d, ^3^*J*_H-H_ = 9.0 Hz, 1H, Ar*H*), 6.62 (s, 1H, Ar*H*), 6.16 (s, 1H, O*H*), 5.61 (brs, 2H, N*H*_2_); **^13^C NMR** (DMSO-d_6_, 75 MHz): δ_C_ 144.7, 144.4, 132.8, 128.4, 128.3, 127.8, 127.0, 126.3, 126.0, 125.9, 122.1, 120.6, 119.9, 116.0 (Ar*Cs*), 68.5 (*C*HOH); **HRMS (ESI^+^)** MeOH/CHCl_3_, *m/z* (RI): 250.1214 ([M + H]^+^, 36%). [M + H]^+^ calcd. for C_17_H_15_NO, 250.1226.
*1-(1-Hydroxyethyl)-2-aminonaphthalene* (**3c**): Colorless solid; 98% yield (499 mg); m.p. 89.9–91.2 °C; **^1^H NMR** (CDCl_3_, 300 MHz): δ_H_ 7.78 (d, ^3^*J*_H-H_ = 9.0 Hz, 1H, Ar*H*), 7.70 (d, ^3^*J*_H-H_ = 9.0 Hz, 1H, Ar*H*), 7.58 (d, ^3^*J*_H-H_ = 9.0 Hz, 1H, Ar*H*), 7.41 (t, ^3^*J*_H-H_ = 6.0 Hz, 1H, Ar*H*), 7.23 (t, ^3^*J*_H-H_ = 6.0 Hz, 1H, Ar*H*), 6.87 (d, ^3^*J*_H-H_ = 9.0 Hz, 1H, Ar*H*), 5.91 (q, ^3^*J*_H-H_ = 7.5 Hz, 1H, Ar*H*), 4.82 (brs, 1H, O*H*), 3.33 (brs, 2H, N*H*_2_), 1.65 (d, ^3^*J*_H-H_ = 7.5 Hz, 3H, C*H*_3_); **^13^C NMR** (CDCl_3_, 75 MHz): δ_C_ 142.8, 131.6, 128.8, 128.7, 127.9, 126.6, 121.8, 120.6, 120.2, 117.4 (Ar*Cs*), 67.0 (C*H*OH), 20.2 (*C*H_3_); **HRMS (ESI^+^)** MeOH/CHCl_3_, *m/z* (RI)**:** 188.1068 ([M + H]^+^, 100%). [M + H]^+^ calcd. for C_12_H_13_NO, 188.1070.
*1-(2-Aminonaphthalen-1-yl)butan-1-ol* (**3d**): Orange solid; 95% yield (480 mg); m.p. 86–88 °C; **^1^H NMR** (DMSO-d_6_, 300 MHz): δ_H_ 7.87 (d, ^3^*J*_H-H_ = 6.0 Hz, 1H, Ar*H*), 7.64 (d, ^3^*J*_H-H_ = 9.0 Hz, 1H, Ar*H*), 7.52 (d, ^3^*J*_H-H_ = 9.0 Hz, 1H, Ar*H*), 7.32 (t, ^3^*J*_H-H_ = 7.5 Hz, 1H, Ar*H*), 7.10 (t, ^3^*J*_H-H_ = 7.5 Hz, 1H, Ar*H*), 6.97 (d, ^3^*J*_H-H_ = 9.0, 1H, Ar*H*), 5.64 (brs, 2H, C*H*OH, O*H*), 5.53 (brs, 2H, N*H*_2_), 2.01–1.89 (m, 1H, C*H*_2_CH_2_CH_3_), 1.73–1.62 (m, 1H, C*H*_2_CH_2_CH_3_), 1.57–1.46 (m, 1H, CH_2_C*H*_2_CH_3_), 1.34–1.23 (m, 1H, CH_2_C*H*_2_CH_3_), 0.89 (t, ^3^*J*_H-H_ = 7.5 Hz, 3H, CH_2_CH_2_C*H*_3_); **^13^C NMR** (DMSO-d_6_, 75 MHz): δ_C_ 144.5, 132.2, 128.4, 127.7, 126.9, 121.2, 120.5, 120.0, 116.5, 68.1 (*C*HOH), 37.8, 19.1, 14.1 (CH_2_CH_2_CH_3_ and CH_3_); **HRMS (ESI^+^)** MeOH/CHCl_3_, *m*/*z* (RI): 216.1375 ([M + H]^+^, 100%). [M + H]^+^ calcd. for C_14_H_17_NO, 216.1383.
*1-(2-Amimo-6-bromonaphthalen-1-yl)-2-phenylethanol* (**3e**): Purple solid; 93% yield (468 mg); m.p. 144–146 °C; **^1^H NMR** (DMSO-d_6_, 300 MHz): δ_H_ 7.86 (s, 1H, Ar*H*), 7.54 (d, *J* = 9.0 Hz, 1H, Ar*H*), 7.35 (d, ^3^*J*_H-H_ = 9.0 Hz, 1H, Ar*H*), 7.25–7.21 (m, 4H, Ar*H*), 7.18–7.12 (m, 2H, Ar*H*), 7.06 (d, ^3^*J*_H-H_ = 9.0, 1H, Ar*H*), 5.80 (brs, 2H, N*H*_2_), 5.61 (brs, 1H, O*H*), δ 5.69 (s, 1H, C*H*OH), 3.20 (dd, ^2^*J*_H-H_ = 15.0 Hz, ^3^*J*_H-H_ *=* 9.0 Hz, 1H, C*H*_2_Ph), 2.96 (dd, ^3^*J*_H-H_ = 12.0 Hz, ^3^*J*_H-H_ = 6.0 Hz, 1H, C*H*_2_Ph); **^13^C NMR** (DMSO-d_6_, 75 MHz): δ_C_ 145.1, 140.5, 139.3, 130.7, 129.9, 129.5, 128.4, 128.2, 127.9, 127.3, 125.8, 121.1, 115.9, 113.0 (ArCs), 69.6 (*C*HOH), 40.4 (*CH_2_*Ph); **HRMS (ESI^+^)** MeOH/CHCl_3_, *m/z* (RI): 342.0495 ([M + H]^+^, 100%). [M + H]^+^ calcd. for C_18_H_16_BrNO, 342.0488.
*(2-Amino-6-bromonaphthalen-1-yl)(phenyl)methanol* (**3f**): Very pale orange solid; 94% yield (474 mg); m.p. 171–172 °C; **^1^H NMR** (DMSO-d_6_, 300 MHz): δ_H_ 7.94–7.89 (m, 2H, Ar*H*), 7.58 (d, ^3^*J*_H-H_ = 9.0 Hz, 1H, Ar*H*), 7.34 (d, ^3^*J*_H-H_ = 9.0 Hz, 3H, Ar*H*), 7.25 (t, ^3^*J*_H-H_ = 7.5 Hz, 2H, Ar*H*), 7.16 (t, ^3^*J*_H-H_ = 6.0 Hz, 1H, Ar*H*), 7.06 (d, ^3^*J*_H-H_ = 9.0 Hz, 1H, Ar*H*), 6.54 (s, 1H, Ar*H*), 6.17 (s, 1H), 5.72 (brs, 2H, N*H*_2_); **^13^C NMR** (DMSO-d_6_, 75 MHz): δ_C_ 145.2, 144.2, 131.4, 129.8, 128.4, 127.8, 127.7, 126.3, 125.9, 124.9, 120.9, 116.1, 113.1 (Ar*Cs*), 68.2 (*C*HOH); **HRMS (ESI^+^)** MeOH/CHCl_3_, *m/z* (RI): 328.0319 ([M + H]^+^, 76%). [M + H]^+^ calcd. for C_17_H_14_BrNO, 328.0332.
*1-(1-Hydroxyethyl)-2-amino-6-bromonaphthalene* (**3g**): Colorless solid; 98% yield (495 mg); m.p. 120–123 °C; **^1^H NMR** (DMSO-d_6_, 300 MHz): δ_H_ 7.90 (s, 1H, Ar*H*), 7.87 (d, ^3^*J*_H-H_ = 3.0 Hz, 1H, Ar*H*), 7.50 (d, ^3^*J*_H-H_ = 9.0 Hz, 1H, Ar*H*), 7.39 (d, ^3^*J*_H-H_ = 3.0 Hz, 1H, Ar*H*), 6.99 (d, ^3^*J*_H-H_ = 9.0 Hz, 1H, Ar*H*), 5.74 (brs, 2H, N*H*_2_), 5.59 (q, ^3^*J*_H-H_ = 6.0 Hz, 1H, C*H*OH), 5.52 (brs, 1H, O*H*), 1.41 (d, ^3^*J*_H-H_ = 6.0 Hz, 3H, C*H*_3_); **^13^C NMR** (DMSO-d_6_, 75 MHz): δ_C_ 144.6, 130.3, 129.9, 128.4, 128.2, 126.9, 123.7, 121.0, 117.1, 112.9 (Ar*Cs*), 64.4 (*C*HOH), 21.1 (*C*H_3_); **HRMS (ESI^+^)** MeOH/CHCl_3_, *m/z* (RI)**:** 266.0173 ([M + H]^+^, 100%). [M + H]^+^ calcd. for C_12_H_12_BrNO, 266.0175.
*1-(2-Amino-6-bromonaphthalen-1-yl)butan-1-ol* (**3h**): Brown solid; 98% yield (493 mg); m.p. 138–140 °C; **^1^H NMR** (DMSO-d_6_, 300 MHz): δ_H_ 7.87 (d, ^4^*J*_H-H_ = 1.5 Hz, 2H, Ar*H*), 7.52 (d, ^3^*J*_H-H_ = 9.0 Hz, 1H, Ar*H*), 7.40 (d, ^3^*J*_H-H_ = 9.0 Hz, 1H, Ar*H*), 7.01 (d, ^3^*J*_H-H_ = 9.0, 1H, Ar*H*), 5.80 (brs, 2H, N*H*_2_), 5.61 (brs, 1HO*H*), 5.42 (t, ^3^*J*_H-H_ = 6.0 Hz, 1H, C*H*OH), 1.96–1.84 (m, 1H, C*H*_2_CH_2_CH_3_), 1.69–1.56 (m, 1H, C*H*_2_CH_2_CH_3_), 1.54–1.42 (m, 1H, CH_2_C*H*_2_CH_3_), 1.30–1.18 (m, 1H, CH_2_C*H*_2_CH_3_), 0.87 (t, ^3^*J*_H-H_ = 7.5 Hz, 3H, CH_2_CH_2_C*H*_3_); **^13^C NMR** (DMSO-d_6_, 75 MHz): δ_C_ 144.7, 130.8, 129.9, 128.4, 127.0, 121.1, 116.9, 113.0 (Ar*Cs*), 68.0 (*C*HOH), 36.9, 19.1, 14.1 (*CH*_2_*C*H_2_*C*H_3_); **HRMS (ESI^+^)** MeOH/CHCl_3_, *m/z* (RI) (RI): 294.0490 ([M + H]^+^, 3%), 276.0390 ([M + H-H_2_O]^+^, 100%). [M + H]^+^ calcd. for C_14_H_16_BrNO, 294.0488.

General procedure for the synthesis of 14-aza-12-oxasteroid analogues (**4a–l**):

In a 50 mL RB flask with a stir bar, **3a–h** (200 mg, 1 equiv.) was dissolved in toluene (ca. 5 mL), and then the appropriate keto acid [levulinic acid/2-carboxybenzaldehyde/4-acetylbutyric acid/(2-oxopropyl sulfanyl)-acetic acid, 1.5 equiv.] was added. The reaction mixture was refluxed for 2 h using a Dean–Stark apparatus, with dry toluene in the vertical column. At this point, TLC (2% MeOH in DCM) revealed complete consumption of the starting materials and formation of the product. The solvent was removed from the reaction mixture in vacuo and the crude product was purified using column chromatography (using DCM as eluent and slowly increasing the polarity with MeOH) to yield the respective 14-aza-12-oxasteroid analogues.

*rac-(11R,12aS)-11-benzyl-12a-methyl-11,12a-dihydro-1H-naphtho[2,1-d]pyrrolo[2,1-b][1,3]oxazin-3(2H)-one* (**4a**): Orange solid; 40% yield (104 mg); R_f_ 0.55 (2% MeOH in DCM); m.p. 124–126 °C; **^1^H NMR** (CDCl_3_, 300 MHz): δ_H_ 8.16 (d, ^3^*J*_H-H_ = 9.0 Hz, 1H, Ar*H*), 7.93 (t, ^3^*J*_H-H_ = 7.5 Hz, 2H, Ar*H*), 7.84 (d, ^3^*J*_H-H_ = 9.0 Hz, 1H, Ar*H*), 7.62 (t, ^3^*J*_H-H_ = 7.5 Hz, 1H, Ar*H*), 7.52 (t, ^3^*J*_H-H_ = 7.5 Hz, 1H, Ar*H*), 7.27–7.22 (m, 3H, Ar*H*), 7.07 (d, ^4^*J*_H-H_ = 3.0, 2H, Ar*H*), 5.85 (d, ^3^*J*_H-H_ = 3.0 Hz, 1H, PhCH_2_C*H*), 3.43 (d, ^2^*J*_H-H_ = 12.0 Hz, 1H, Ph*CH_2_*CH), 3.06 (dd, ^2^*J*_H-H_ = 15.0 Hz, ^3^*J*_H-H_ = 6.0 Hz, 1H, Ph*CH_2_*CH), 2.58 (t, ^3^*J*_H-H_ = 7.5 Hz, 2H, *CH_2_*CH_2_CO), 2.37–2.20 (m, 2H, CH_2_*CH_2_*CO), 1.42 (s, 3H, *CH_3_*); **^13^C NMR** (DMSO, 75 MHz): δ_C_ 172.1 (*C*O), 137.7, 131.4, 129.5, 129.1, 128.4, 127.8, 126.5, 126.5, 124.9, 122.6, 121.8, 120.7 (Ar*Cs*), 89.3 (*C*12), 72.1 (*C*11), 42.8 (Ph*CH_2_*), 32.6, 29.8, 22.4 (*C*H_2_*C*H_2_ and *C*H_3_); **HRMS (ESI^+^)** MeOH/CHCl_3_, *m/z* (RI): 344.1644 ([M + H]^+^, 100%). [M + H]^+^ calcd. for C_23_H_21_NO_2_, 344.1645.
*rac-(11R,12aS)-12a-methyl-11-phenyl-11,12a-dihydro-1H-naphtho[2,1-d]pyrrolo[2,1-b][1,3]oxazin-3(2H)-one* (**4b**): Yellow-orange powder; 45% yield (119 mg); R_f_ 0.46 (2% MeOH in DCM); m.p. 189–191 °C; **^1^H NMR** (CDCl_3_, 300 MHz): δ_H_ 8.46 (d, ^3^*J*_H-H_ = 9.0 Hz, 1H, Ar*H*), 7.90 (d, ^3^*J*_H-H_ = 9.0 Hz, 1H, Ar*H*), 7.83 (d, ^3^*J*_H-H_ = 9.0 Hz, 1H, Ar*H*), 7.48 (d, ^3^*J*_H-H_ = 9.0 Hz, 1H, Ar*H*), 7.36 (t, ^3^*J*_H-H_ = 6.0 Hz, 1H, Ar*H*), 7.32–7.26 (m, 6H, Ar*H*), 6.45 (s, 1H, PhC*H*), 2.71–2.66 (m, 2H, *CH_2_*CH_2_CO), 2.29 (t, ^3^*J*_H-H_ = 9.0 Hz, 2H, CH_2_*CH_2_*CO), 1.64 (s, 3H, *CH_3_*); **^13^C NMR** (DMSO-d_6_, 75 MHz): δ_C_ 172.6 (*C*O), 141.6, 132.3, 131.5, 129.7, 129.5, 129.2, 129.0, 129.0, 128.8, 126.6, 125.2, 124.6, 120.7, 120.6 (Ar*Cs*), 90.6 (*C*12), 75.8 (*C*11), 33.1, 30.6, 22.0 (*C*H_2_*C*H_2_ and *C*H_3_); **HRMS (ESI^+^)** MeOH/CHCl_3_, *m/z* (RI): 330.1489 ([M + H]^+^, 100%). [M + H]^+^ calcd. for C_22_H_19_NO_2_, 330.1489.
*rac-(11R,12aS)-11,12a-dimethyl-11,12a-dihydro-1H-naphtho[2,1-d]pyrrolo[2,1-b][1,3]oxazin-3(2H)-one* (**4c**): Viscous orange liquid; 34% yield (96 mg); R_f_ 0.39 (2% MeOH in DCM); **^1^H NMR** (CDCl_3_, 300 MHz): δ_H_ 8.35 (d, ^4^*J*_H-H_ = 1.5 Hz, 1H, Ar*H*), 7.87–7.78 (m, 3H, Ar*H*), 7.53–7.43 (m, 2H, Ar*H*), 5.66 (q, ^3^*J*_H-H_ = 6.0 Hz, 1H, CH_3_C*H*), 2.67 (t, ^3^*J*_H-H_ = 9.0 Hz, 2H, *CH*_2_CH*_2_*CO), 2.30 (t, ^3^*J*_H-H_ = 9.0 Hz, 2H, C*H*_2_*C*H*_2_*CO), 1.65 (d, ^3^*J*_H-H_ = 6.0 Hz, 3H, C*H*_3_), 1.47 (s, 3H, C*H*_3_); **^13^C NMR** (CDCl_3_, 75 MHz): δ_C_ 172.1 (*C*O), 131.2, 130.2, 129.2, 128.9, 128.2, 126.2, 124.8, 123.3, 123.1, 120.4 (Ar*Cs*), 89.3 (*C*12), 68.0 (*C*11), 32.8, 30.1, 23.1, 21.5 (*C*H_2_*C*H_2_ and *C*H_3_); **HRMS (ESI^+^)** MeOH/CHCl_3_, *m/z* (RI)**:** 268.1332 ([M + H]^+^, 100%). [M + H]^+^ calcd. for C_17_H_17_NO_2_, 268.1331.
*rac-(11R,12aS)-12a-methyl-11-propyl-11,12a-dihydro-1H-naphtho[2,1-d]pyrrolo[2,1-b][1,3]oxazin-3(2H)-one* (**4d**): Orange solid; yield 45% (123 mg); R_f_ 0.40 (2% MeOH in DCM); m.p. 123–125 °C; **^1^H NMR** (CDCl_3_, 300 MHz): δ_H_ 8.27 (d, ^3^*J*_H-H_ = 9.0 Hz, 1H, Ar*H*), 7.87–7.76 (m, 3H, Ar*H*), 7.52 (t, ^3^*J*_H-H_ = 9.0 Hz, 1H, Ar*H*), 7.45 (t, ^3^*J*_H-H_ = 9.0 Hz, 1H, Ar*H*), 5.58 (dd, ^3^*J*_H-H_ = 6.0 Hz, ^4^*J*_H-H_ = 1.5 Hz, 1H, CH_3_C*H*), 2.67 (t, ^3^*J*_H-H_ = 7.5 Hz, 2H, *CH*_2_CH*_2_*CO), 2.30 (t, ^3^*J*_H-H_ = 7.5 Hz, 2H, *C*H_2_C*H_2_*CO), 2.12–2.01 (m, 1H, C*H*_2_CH_2_CH_3_), 1.87–1.75 (m, 1H, C*H*_2_CH_2_CH_3_), 1.55–1.44 (m, 1H, CH_2_C*H*_2_CH_3_), 1.44 (s, 3H, C*H*_3_), 1.32–1.21 (m, 1H, CH_2_C*H*_2_CH_3_), 0.87 (t, ^3^*J*_H-H_ = 6.0 Hz, 3H, CH_2_CH_2_C*H*_3_); **^13^C NMR** (DMSO-d_6_, 75 MHz): δ_C_ 172.3 (*C*O), 131.2, 131.0, 129.2, 128.9, 128.1, 126.2, 124.8, 122.9, 122.6, 120.5 (Ar*Cs*), 89.3 (*C*12), 71.3 (*C*11), 38.8, 32.8, 30.1, 21.9, 18.0, 13.9 (*C*H_2_*C*H_2,_ *C*H_3_, *n-*Pr); **HRMS (ESI^+^)** MeOH/CHCl_3_, *m/z* (RI): 296.1631 ([M + H]^+^, 100%). [M + H]^+^ calcd. for C_19_H_21_NO_2_, 296.1645.
*rac-(11R,12aS)-11-benzyl-8-bromo-12a-methyl-11,12a-dihydro-1H-naphtho[2,1-d]pyrrolo[2,1-b][1,3]oxazin-3(2H)-one* (**4e**): Purple solid; 33% yield (82 mg); R_f_ 0.49 (2% MeOH in DCM); m.p. 106–108 °C; **^1^H NMR** (CDCl_3_, 300 MHz): δ_H_ 8.16 (d, ^3^*J*_H-H_ = 9.0 Hz, 1H, Ar*H*), 8.05 (d, ^4^*J*_H-H_ = 1.5 Hz, 1H), 7.79 (d, ^3^*J*_H-H_ = 9.0 Hz, 1H, Ar*H*), 7.72 (d, ^3^*J*_H-H_ = 9.0 Hz, 1H, Ar*H*), 7.66 (d, ^3^*J*_H-H_ = 9.0 Hz, 1H, Ar*H*), 7.23–7.19 (m, 3H, Ar*H*), 7.00–6.99 (m, 2H, Ar*H*), 5.79 (dd, ^3^*J*_H-H_ = 6.0 Hz, ^4^*J*_H-H_ = 3.0 Hz, 1H, PhCH_2_C*H*), 3.36 (dd, ^2^*J*_H-H_ = 15.0 Hz, ^3^*J*_H-H_ = 3.0 Hz, 1H, Ph*CH_2_*CH), 3.04 (dd, ^2^*J*_H-H_ = 15.0 Hz, ^3^*J*_H-H_ = 6.0 Hz, 1H, Ph*CH_2_*CH), 2.56 (t, ^3^*J*_H-H_ = 7.5 Hz, 2H, *CH_2_*CH_2_CO), 2.18–2.34 (m, 2H, *C*H*_2_*C*H*_2_CO), 1.40 (s, 3H, C*H*_3_); **^13^C NMR** (DMSO-d_6_, 75 MHz): δ_C_ 172.0 (*C*O), 137.2, 132.3, 131.7, 131.0, 129.8, 129.4, 127.8, 127.5, 126.6, 124.4, 122.0, 121.8, 118.8 (Ar*Cs*), 71.9 (*C*12), 89.3 (*C*11), 42.8 (Ph*CH_2_*), 32.6, 29.8, 22.3 (CH_2_CH_2_, CH_3_); **HRMS (ESI^+^)** MeOH/CHCl_3_, *m/z* (RI): 421.0740 ([M + H]^+^, 100%). [M + H]^+^ calcd. for C_23_H_20_BrNO_2_, 421.0677.
*rac-(11R,12aS)-8-bromo-12a-methyl-11-phenyl-11,12a-dihydro-1H-naphtho[2,1-d]pyrrolo[2,1-b][1,3]oxazin-3(2H)-one* (**4f**): Purple-brown solid; 30% yield (75 mg); R_f_ 0.46 (2% MeOH in DCM); m.p. 203–205 °C; **^1^H NMR** (CDCl_3_, 300 MHz): δ_H_ 8.49 (d, ^3^*J*_H-H_ = 9.0 Hz, 1H, Ar*H*), 7.97 (s, 1H, Ar*H*), 7.79 (d, ^3^*J*_H-H_ = 9.0 Hz, 1H, Ar*H*), 7.31–7.27 (m, 5H, Ar*H*), 7.23–7.21 (m, 2H, Ar*H*), 6.39 (s, 1H, PhC*H*), 2.70–2.65 (m, 2H, *CH_2_*CH_2_CO), 2.29 (t, ^3^*J*_H-H_ = 6.0 Hz, 2H, *C*H*_2_*C*H*_2_CO), 1.64 (s, 3H, C*H*_3_); **^13^C NMR** (DMSO-d_6_, 75 MHz): δ_C_ 172.2 (*C*O), 140.9, 132.3, 132.2, 130.6, 129.5, 128.9, 128.5, 128.1, 127.8, 125.9, 121.4, 120.3, 118.8 (Ar*Cs*), 90.2 (*C*12), 75.2 (*C*11), 32.7, 30.1, 21.6 (*C*H_2_*C*H_2_, *C*H_3_); **HRMS (ESI^+^)** MeOH/CHCl_3_, *m/z* (RI): 408.0584 ([M + H]^+^, 100%). [M + H]^+^ calcd. for C_22_H_18_BrNO_2_, 408.0594.
*rac-(11R,12aS)-8-bromo-11,12a-dimethyl-11,12a-dihydro-1H-naphtho[2,1-d]pyrrolo[2,1-b][1,3]-oxazin-3(2H)-one* (**4g**): Viscous orange liquid; 35% yield (92 mg); R_f_ 0.41 (2% MeOH in DCM); **^1^H NMR** (CDCl_3_, 300 MHz): δ_H_ 8.35 (d, ^4^*J*_H-H_ = 1.5 Hz, 1H, Ar*H*), 7.98 (s, 1H, Ar*H*), 7.69–7.39 (m, 3H, Ar*H*), 5.58 (q, ^3^*J*_H-H_ = 6.0 Hz, 1H, CH_3_C*H*), 2.65 (t, ^3^*J*_H-H_ = 7.5 Hz, 2H, *CH_2_*CH_2_CO), 2.27 (t, ^3^*J*_H-H_ = 7.5 Hz, 2H, *C*H*_2_*C*H*_2_CO), 1.59 (d, ^3^*J*_H-H_ = 6.0 Hz, 3H, C*H*_3_), 1.43 (s, 3H, C*H*_3_); **^13^C NMR** (CDCl_3_, 75 MHz): δ_C_ 172.2 (*C*O), 132.3, 130.7, 129.4, 127.7, 127.2, 124.8, 123.5, 121.4, 119.0, 118.7 (Ar*Cs*), 89.3 (*C*12), 67.7 (*C*11), 32.7, 30.0, 23.1, 21.4 (*C*H_2_*C*H_2_, *C*H_3_); **HRMS (ESI^+^)** MeOH/CHCl_3_, *m/z* (RI)**:** 346.0433 ([M + H]^+^, 100%). [M + H]^+^ calcd. for C_17_H_16_BrNO_2_, 346.0437.
*rac-(11R,12aS)-8-Bromo-12a-methyl-11-propyl-11,12a-dihydro-1H-naphtho[2,1-d]pyrrolo[2,1-b][1,3]-oxazin-3(2H)-one* (**4h**): Purple oil; 40% yield (102 mg); R_f_ 0.62 (2% MeOH in DCM); **^1^H NMR** (CDCl_3_, 300 MHz): δ_H_ 8.29 (d, ^3^*J*_H-H_ = 9.0 Hz, 1H, Ar*H*), 7.99 (d, ^4^*J*_H-H_ = 1.5 Hz, 1H, Ar*H*), 7.70–7.62 (m, 2H, Ar*H*), 7.56 (d, ^3^*J*_H-H_ = 9.0 Hz, 1H, Ar*H*), 5.53 (dd, ^3^*J*_H-H_ = 6.0 Hz, 3.0 Hz, 1H, CH_3_C*H*), 2.66 (t, ^3^*J*_H-H_ = 7.5 Hz, 2H, *CH*_2_CH*_2_*CO), 2.29 (t, ^3^*J*_H-H_ = 7.5 Hz, 2H, *C*H_2_C*H_2_C*O), 2.06–1.95 (m, 1H, *CH*_2_CH_2_CH_3_), 1.82–1.70 (m, 1H, *CH*_2_CH_2_CH_3_), 1.51–1.39 (m, 1H, CH_2_*CH*_2_CH_3_), 1.43 (s, 3H, *CH*_3_), 1.23–1.15 (m, 1H, CH_2_*CH*_2_CH_3_), 0.85 (t, ^3^*J*_H-H_ = 7.5 Hz, 3H, CH_2_CH_2_*CH*_3_); **^13^C NMR** (DMSO-d_6_, 75 MHz): δ_C_ 172.3 (*C*O), 132.4, 131.3, 130.8, 129.4, 127.6, 127.2, 124.6, 122.7, 121.5, 118.7 (Ar*Cs*), 89.3 (*C*12), 71.1 (*C*11), 38.8, 32.8, 30.0, 21.8, 17.9, 13.8 (*C*H_2_*C*H_2,_ *C*H_3_, *n-*Pr); **HRMS (ESI^+^)** MeOH/CHCl_3_, *m/z* (RI): 374.0739 ([M + H]^+^, 100%). [M + H]^+^ calcd. for C_19_H_20_BrNO_2_, 374.0750.
*7-Methyl-7H-naphtho[2′,1′:4,5][1,3]oxazino[2,3-a]isoindol-13(8aH)-one* (**4i**): Viscous orange liquid; 78% yield (249 mg). R_f_ 0.79 (2% MeOH in DCM); mixture of two diastereomers (6:4). Data for the major diastereomer are presented here; **^1^H NMR** (CDCl_3_, 300 MHz): δ_H_ 8.56 (d, ^4^*J*_H-H_ = 1.5 Hz, 1H, Ar*H*), 7.94–7.43 (m, 9H, Ar*H*), 5.95 (q, ^3^*J*_H-H_ = 9.0 Hz, 1H, CH_3_C*H*), 5.79 (s, 1H, NC*H*O), 1.72 (d, ^3^*J*_H-H_ = 9.0 Hz, 3H, C*H*_3_); **^13^C NMR** (CDCl_3_, 75 MHz): δ_C_ 164.8 (*C*O), 140.2, 132.7, 130.2, 128.9, 128.5, 126.6, 126.4, 124.7, 124.6, 124.0, 123.5, 123.2, 123.1, 122.4, 120.8, 119.0 (Ar*Cs*), 83.1, 72.4, 23.3 (*C*H_3_); **HRMS (ESI^+^)** MeOH/CHCl_3_, *m/z* (RI)**:** 302.1173 ([M + H]^+^, 100%). [M + H]^+^ calcd. for C_20_H_15_NO_2_, 302.1176.
*4-Bromo-7-methyl-7H-naphtho[2′,1′:4,5][1,3]oxazino[2,3-a]isoindol-13(8aH)-one* (**4j**): Colorless solid; 40% yield (112 mg); R_f_ 0.85 (2% MeOH in DCM); m.p. 230–233 °C. Mixture of two diastereomers (8:2). Data for the major diastereomer are presented here; **^1^H NMR** (CDCl_3_, 300 MHz): δ_H_ 8.58 (d, ^3^*J*_H-H_ = 9.0 Hz, 1H, Ar*H*), 8.03 (s, 1H, Ar*H*), 7.95 (d, ^3^*J*_H-H_ = 9.0 Hz, 1H, Ar*H*), 8.03–7.59 (m, 6H, Ar*H*), 5.94 (q, ^3^*J*_H-H_ = 6.0 Hz, 1H, CH_3_C*H*), 5.87 (s, 1H, NC*H*O), 1.70 (d, ^3^*J*_H-H_ = 6.0 Hz, 3H, *CH*_3_); **^13^C NMR** (CDCl_3_, 75 MHz): δ_C_ 164.9 (*C*O), 140.2, 132.9, 132.2, 131.0, 130.5, 130.0, 129.7, 128.2, 127.7, 124.9, 124.2, 123.6, 122.5, 120.3, 118.6 (Ar*Cs*), 83.2 (C-12), 72.3 (C-11), 23.4 (*C*H_3_); **HRMS (ESI^+^)** MeOH/CHCl_3_, *m/z* (RI)**:** 380.0284 ([M + H]^+^, 96%). [M + H]^+^ calcd. for C_20_H_14_BrNO_2_, 380.0281.
*rac-(12R,13aS)-12,13a-dimethyl-1,2,3,13a-tetrahydronaphtho[2,1-d]pyrido[2,1-b][1,3]oxazin-4(12H)-one* (**4k**): Viscous orange liquid; 30% yield (91 mg); R_f_ 0.61 (2% MeOH in DCM); **^1^H NMR** (CDCl_3_, 300 MHz): δ_H_ 7.87–7.70 (m, 4H, Ar*H*), 7.55–7.46 (m, 2H, Ar*H*), 5.65 (q, ^3^*J*_H-H_ = 6.0 Hz, 1H, CH_3_C*H*), 2.63 (t, ^3^*J*_H-H_ = 9.0 Hz, 2H, *CH*_2_CH_2_CH_2_), 2.13 (t, ^3^*J*_H-H_ = 9.0 Hz, 2H, CH_2_*CH*_2_CH_2_), 1.63 (d, ^3^*J*_H-H_ = 6.0 Hz, 3H, C*H*_3_), 1.38 (s, 3H, C*H*_3_), 0.83 (t, ^3^*J*_H-H_ = 9.0 Hz, 2H, CH_2_CH_2_*CH*_2_); **^13^C NMR** (CDCl_3_, 75 MHz): δ_C_ 170.1 (*C*O), 132.8, 131.6, 128.9, 128.8, 127.6, 127.1, 126.2, 125.2, 122.5 (Ar*Cs*), 86.1 (*C*12), 67.3 (*C*11), 37.0, 34.1, 25.0, 23.5, 17.0 (CH_2_CH_2_CH_2_, *C*H_3_); **HRMS (ESI^+^)** MeOH/CHCl_3_, *m/z* (RI)**:** 282.3619 ([M + H]^+^, 100%). [M + H]^+^ calcd. for C_18_H_19_NO_2_: 282.3625.
*rac-(12R,13aS)-12,13a-dimethyl-12,13a-dihydro-1H-naphtho[2,1-d][1,4]thiazino[3,4-b][1,3]oxazin-4(3H)-one* (**4l**): Viscous orange liquid; 30% yield (92 mg); R_f_ 0.62 (2% MeOH in DCM); **^1^H NMR** (CDCl_3_, 300 MHz): δ_H_ 7.87–7.66 (m, 4H, Ar*H*), 7.56–7.47 (m, 2H, Ar*H*), 5.65 (q, ^3^*J*_H-H_ = 7.5 Hz, 1H, CH_3_C*H*), 3.60 (m, 2H, C*H*_2_SCH_2_), 3.06 (s, 2H, CH_2_SC*H*_2_), 1.68 (d, ^3^*J*_H-H_ = 7.5 Hz, 3H, C*H*_3_), 1.54 (s, 3H, C*H*_3_); **^13^C NMR** (CDCl_3_, 75 MHz): δ_C_ 165.6 (CO), 132.1, 131.6, 128.8, 128.7, 127.2, 126.3, 125.5, 124.6, 122.5 (Ar*Cs*), 87.7 (*C*12), 67.5 (*C*11), 39.0, 33.8, 23.4, 23.3 (CH_2_SCH_2_), 2x*C*H_3_); **HRMS (ESI^+^)** MeOH/CHCl_3_, *m/z* (RI)**:** 300.1047 ([M + H]^+^, 100%). [M + H]^+^ calcd. for C_17_H_17_NO_2_S: 300.1053.

## 5. Conclusions

We have devised a concise synthesis of 14-aza-12-oxasteroids, **4a**–**l** in four simple steps, starting from inexpensive and readily available 2-naphthol analogues. The Bucherer reaction was employed to convert naphthol analogues to their corresponding naphthylamines, which in turn were ortho-acylated using the Sugasawa reaction. Furthermore, the 1-acylated-2-aminonaphthylenes were then reduced to the corresponding amino-alcohols. Double dehydrations and double intramolecular cyclizations of the synthesized amino-alcohols with four different oxo-acids resulted in a one-pot formation of a C-N bond, a C-O bond, and an amide bond in tandem. A series of twelve 14-aza-12-oxasteroid analogues were synthesized in moderate yields. The structure, stereochemistry, and connectivity of the bonds were further confirmed by an X-ray crystal structure determination of one of the analogues, 12a-methyl-11-phenyl-11,12a-dihydro-1*H*-naphtho[2,1-d]pyrrolo[2,1-b][1,3]oxazin-3(2*H*)-one (**4b**).

## Data Availability

All spectroscopic and X-ray data obtained during this research are included in the Supplementary Materials (see above).

## Supplementary Materials

The following supporting information can be downloaded at: https://www.mdpi.com/article/10.3390/molecules30020415/s1, ^1^H and ^13^C NMR spectra of all compounds reported in this article. Figure S1: Structure of compound **4b** showing the two molecules of the independent unit in the correct relative orientation. Figure S2: Separate diagrams of the two molecules in compound **4b** showing the full heavy atom labelling. Table S1: Crystal data and structure refinement for Compound **4b**. Refs [36,37,38,39,40,41] are cited in Supplementary Materials.
