# Supplementary material for: Concise Synthesis of Naphthalene-Based 14-Aza-12-Oxasteroids
Source: Molecules. 2025 Jan 19;30(2):415. doi: 10.3390/molecules30020415 (PMC11767449; doi:10.3390/molecules30020415)
Supplement: Supplementary file 1 [file molecules-30-00415-s001.zip › molecules-3333104-supplementary.pdf]

# Concise Synthesis of Naphthalene-based 14-Aza-12-Oxasteroids

**Smriti Srivastava,[1] Jun Luo,[1] Daniel Whalen,[1] Katherine Robertson,[2] Amitabh Jha*,[1]**

[1]Department of Chemistry, Acadia University, Wolfville, NS, B4P 2R6, Canada

[2]Department of Chemistry, Saint Mary's University, Halifax, NS, B3H C3C, Canada

Fax: +1-902-585-1114; Tel: +1-902-585-1515; E-mail:ajha@acadia.ca

Table of Contents

1. General Information S2

2. $^{1}H$ and $^{13}C$ NMR Spectra of compound **2a-2h** S3-S10

3. $^{1}H$ and $^{13}C$ NMR Spectra of compound **3a-3h** S11-18

4. $^{1}H$ and $^{13}C$ NMR Spectra of final compounds **4a-4l** S19-30

5. X-Ray Crystal Data of Compound **4b** S31-33

6. References S34

## General Information

The $^{1}H$ NMR and $^{13}C$ NMR spectra were recorded on a Bruker AC-300 Avance spectrometer or Bruker ARX-300 spectrometer at 300 and at 75.5 MHz, respectively. The chemical shift values are on $\delta$ scale and the coupling constants ($J$) are in Hz. HRMS (ESI) spectra were recorded using Orbitrap Q-exactive analyser. Melting points were determined on a MEL-TEMP II apparatus. All chemicals used were purchased either from Aldrich Chemical Co. or A.K. Scientific, Inc. USA, Canada and used without further purification, except for toluene and methanol. Toluene was dried with sodium metal overnight and distilled before reactions. Methanol was dried with molecular sieves overnight before use. Analytical TLCs were performed on pre-coated Merck silica gel 60F254 plates; the spots were detected under UV light. Silica gel (230-400 mesh) was used for column chromatography. After column chromatography, fraction-containing products were evaporated under reduced pressure and dried under high vacuum to give the desired product.

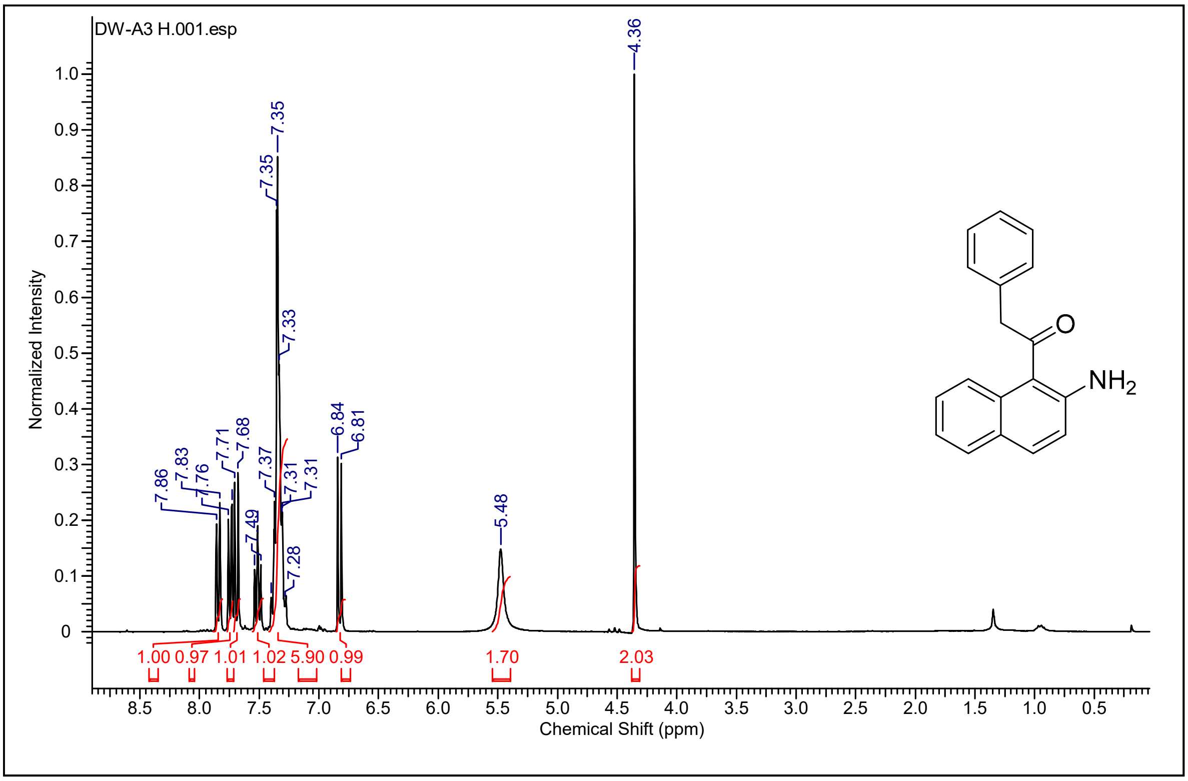


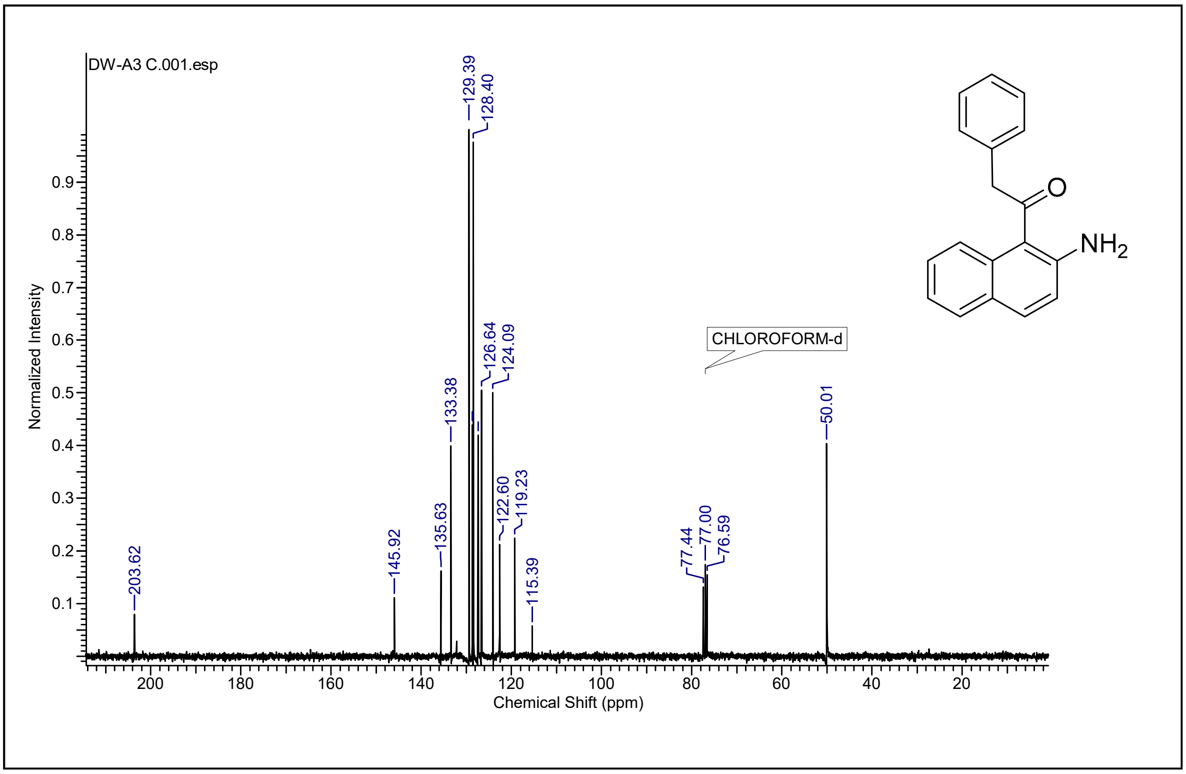


$^{1}H$ and $^{13}C$ NMR spectra of compound **2a**

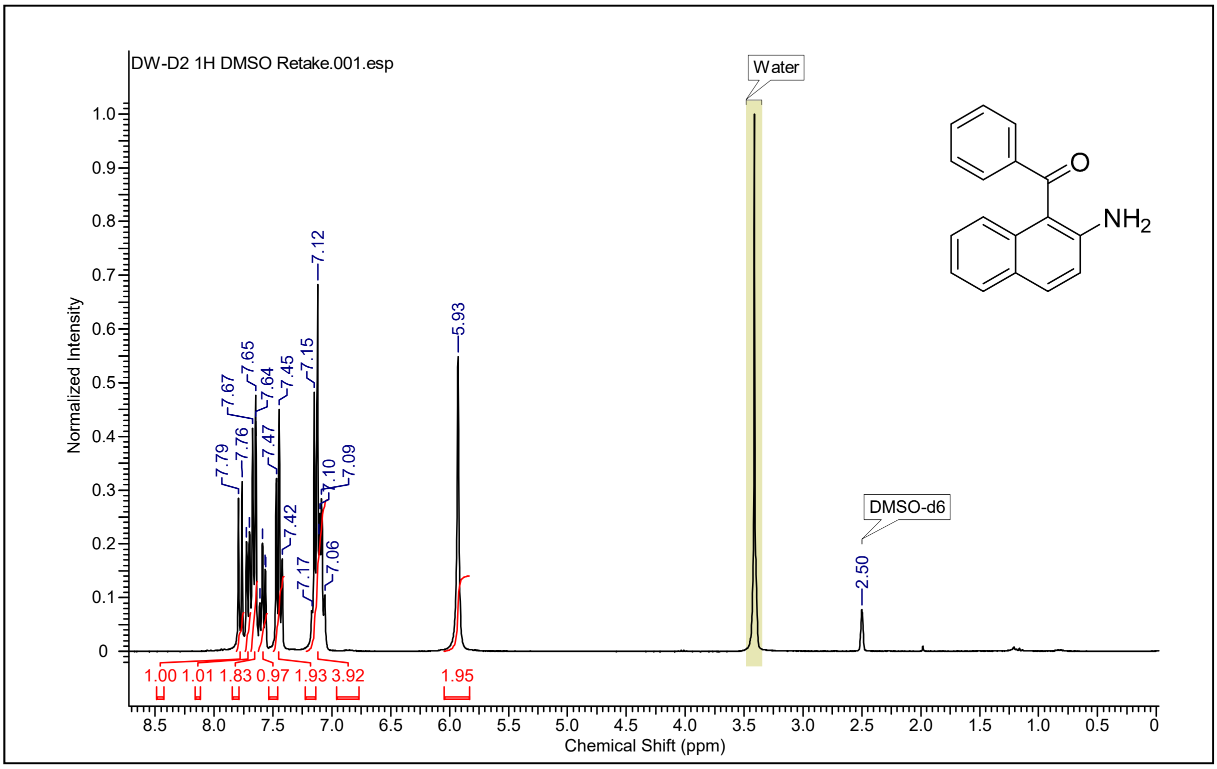


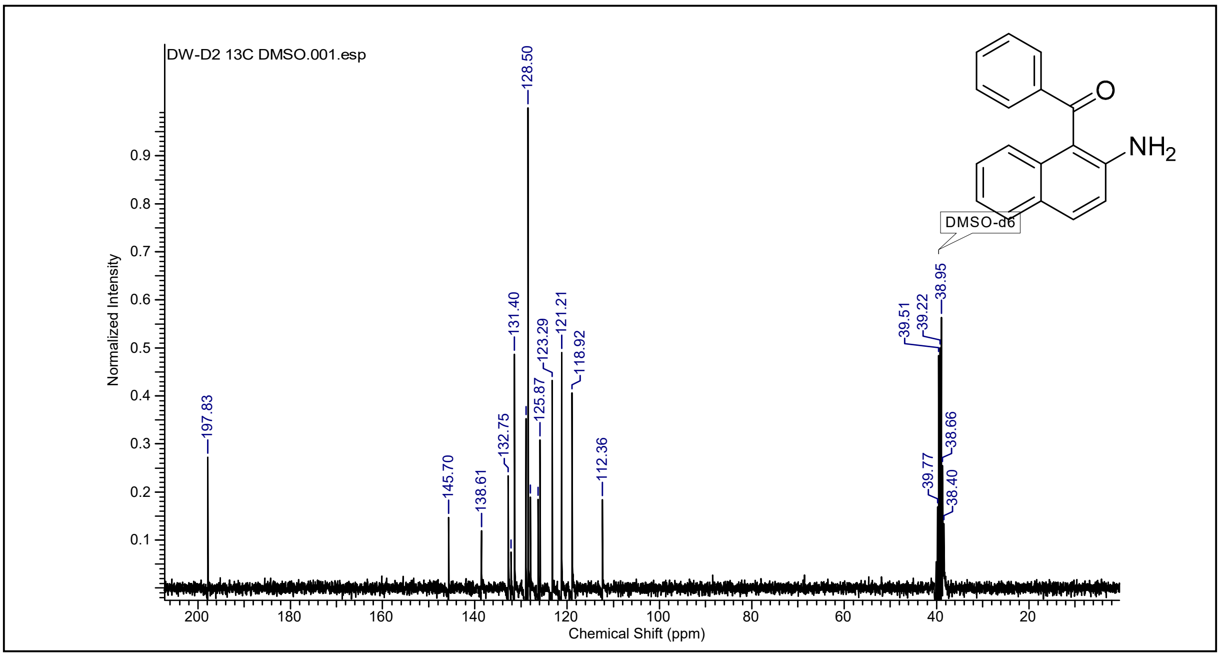


$^1$H and $^{13}$C NMR spectra of compound **2b**

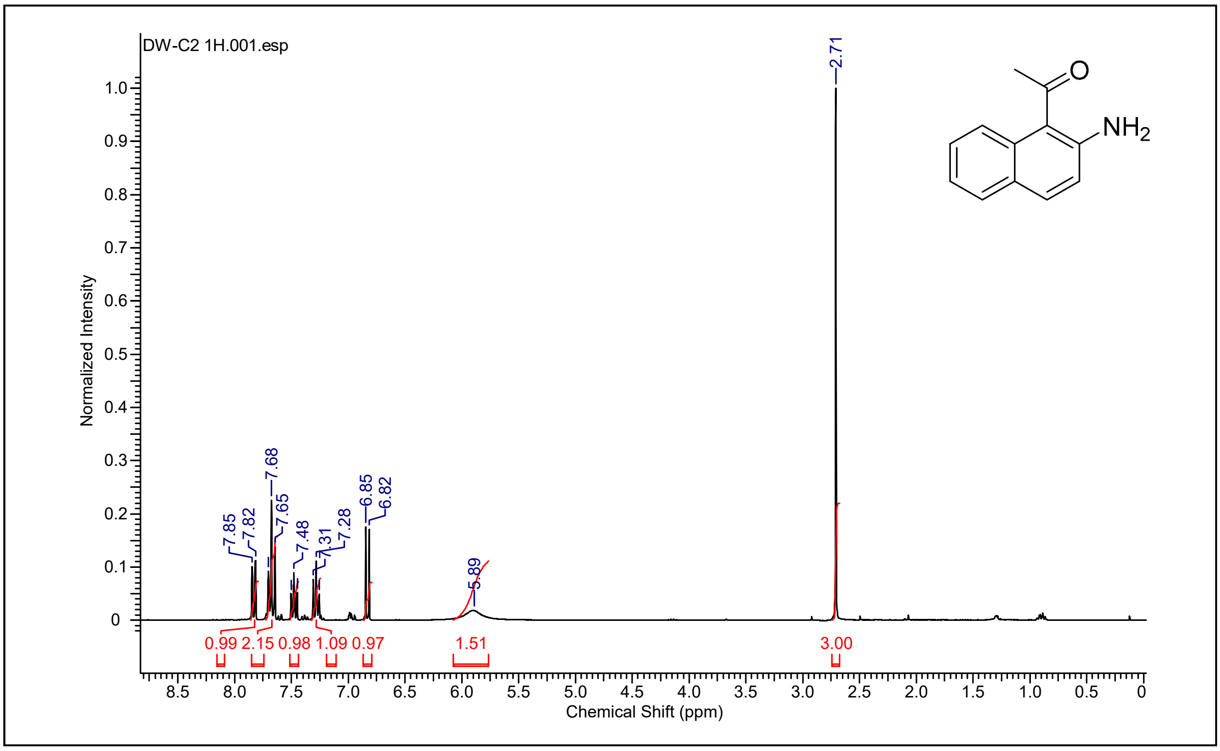


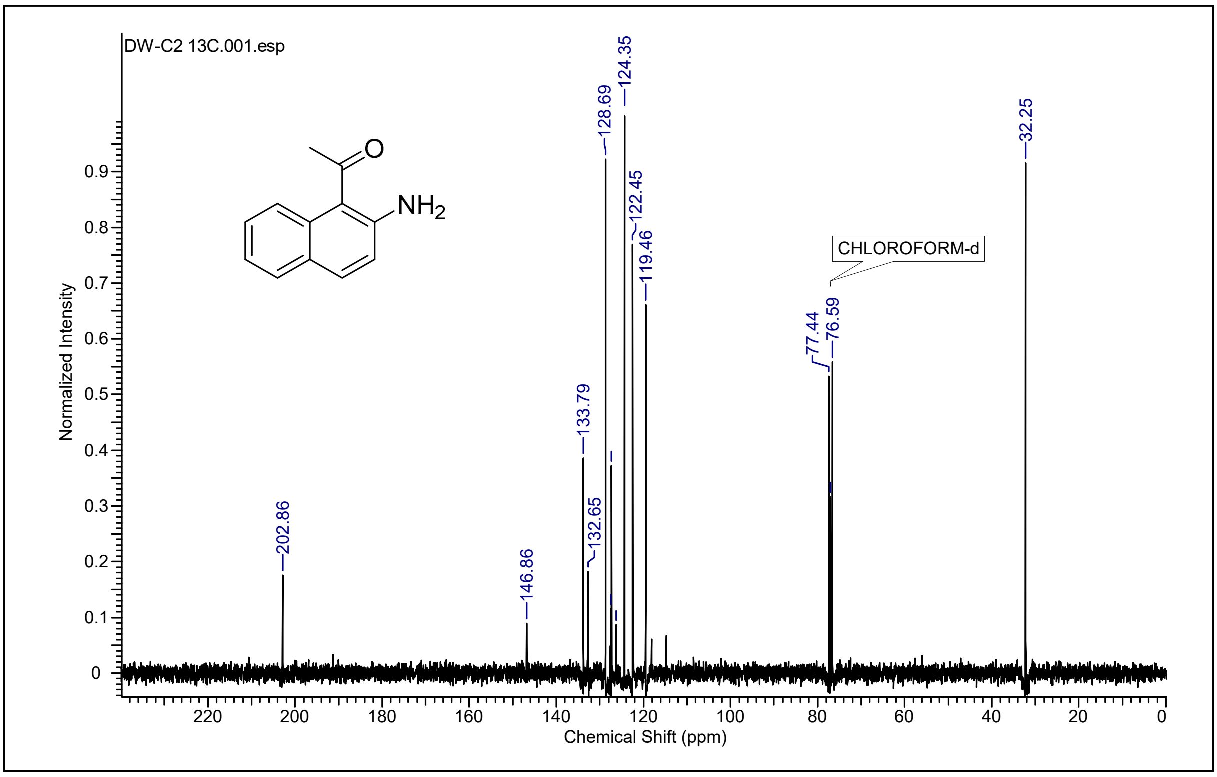


$^{1}H$ and $^{13}C$ NMR spectra of compound **2c**

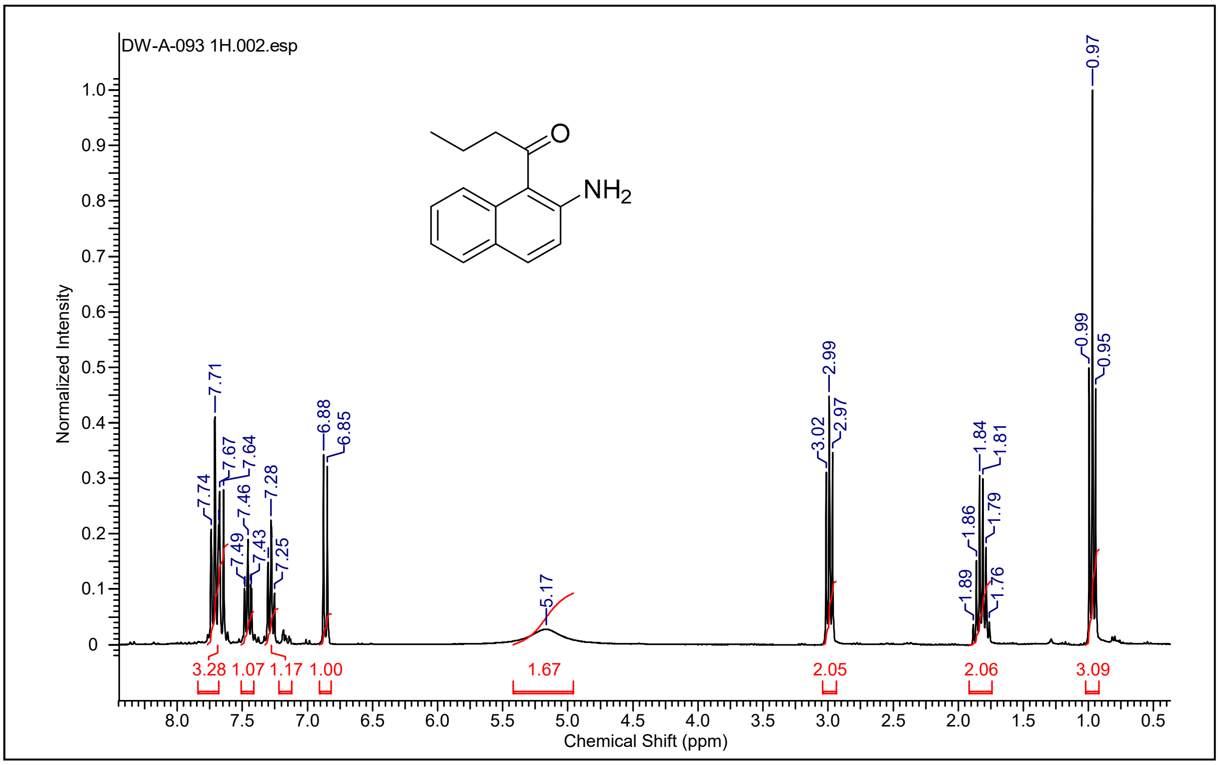


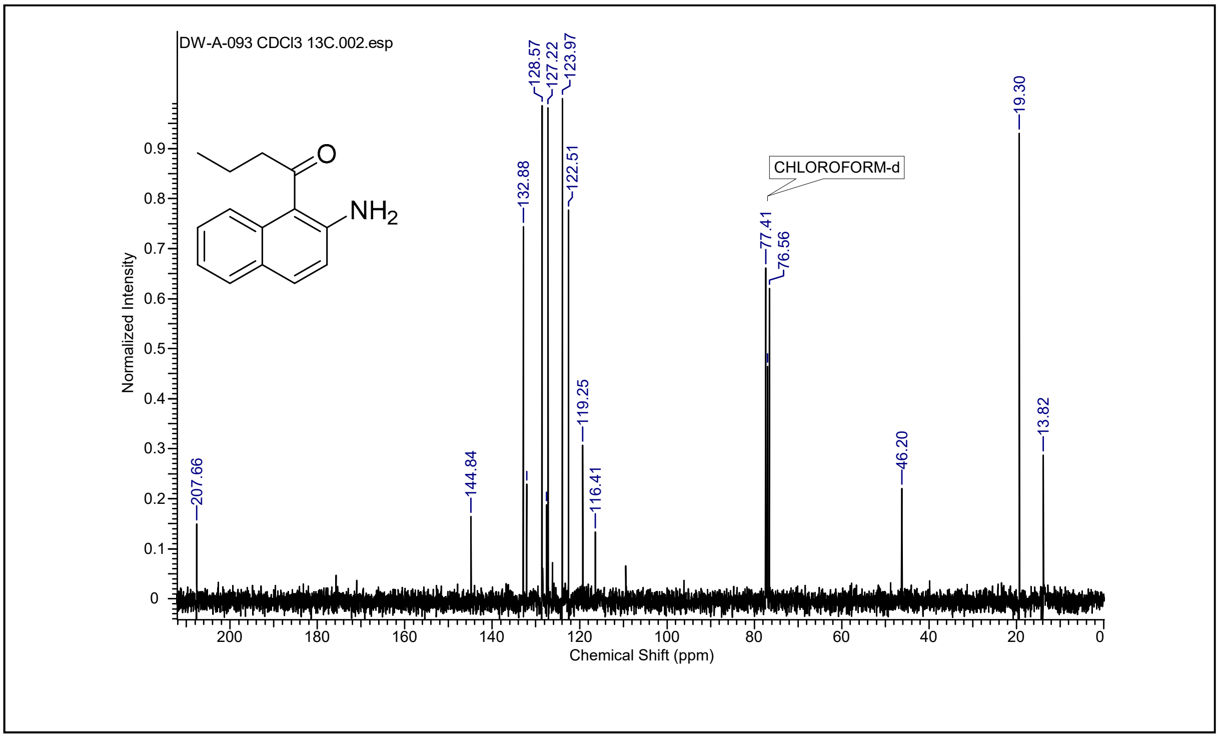


$^{1}$H and $^{13}$C NMR spectra of compound **2d**

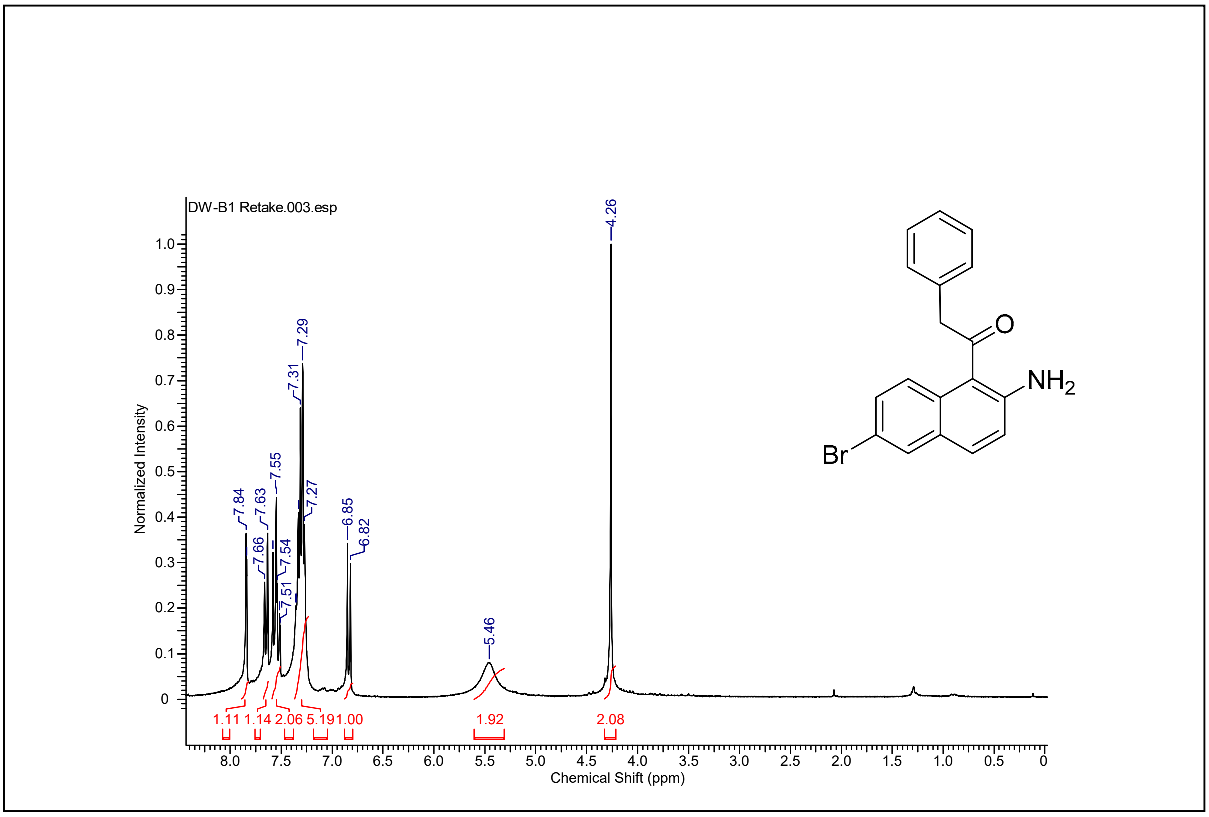


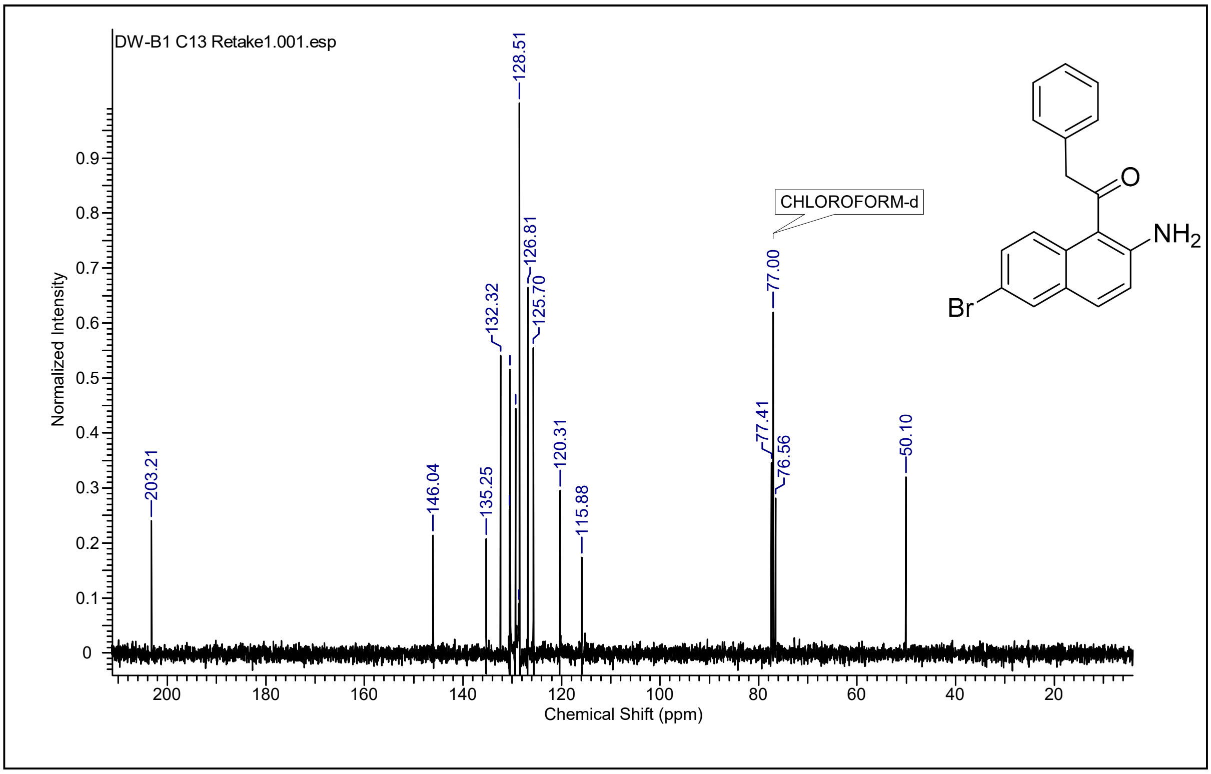


$^{1}$H and $^{13}$C NMR spectra of compound **2e**

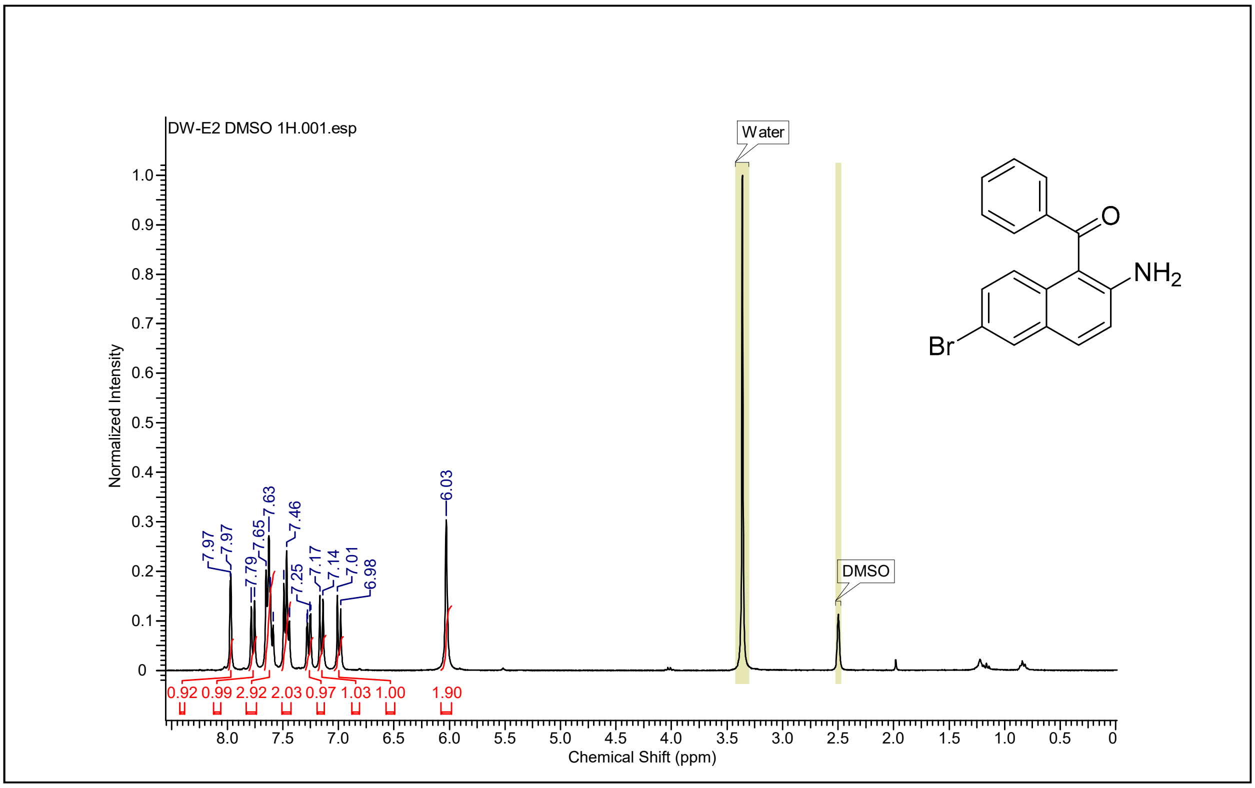


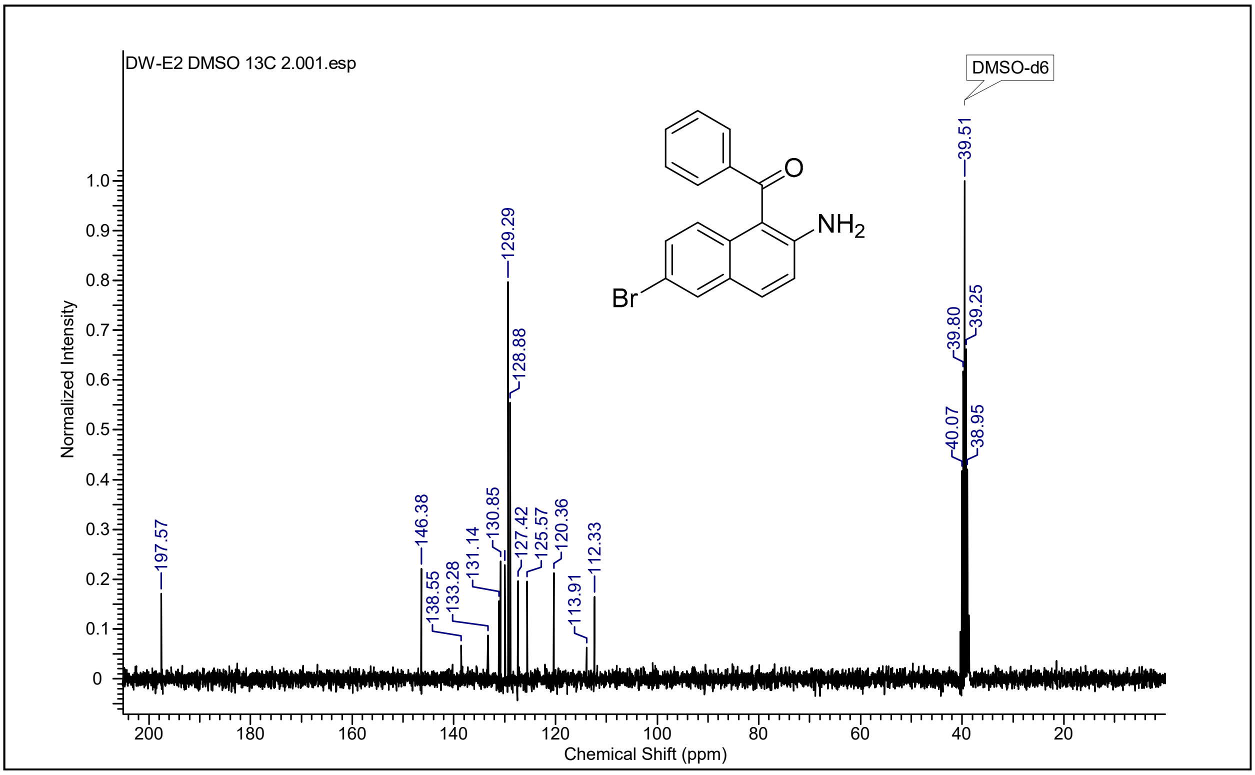


$^{1}$H and $^{13}$C NMR spectra of compound **2f**

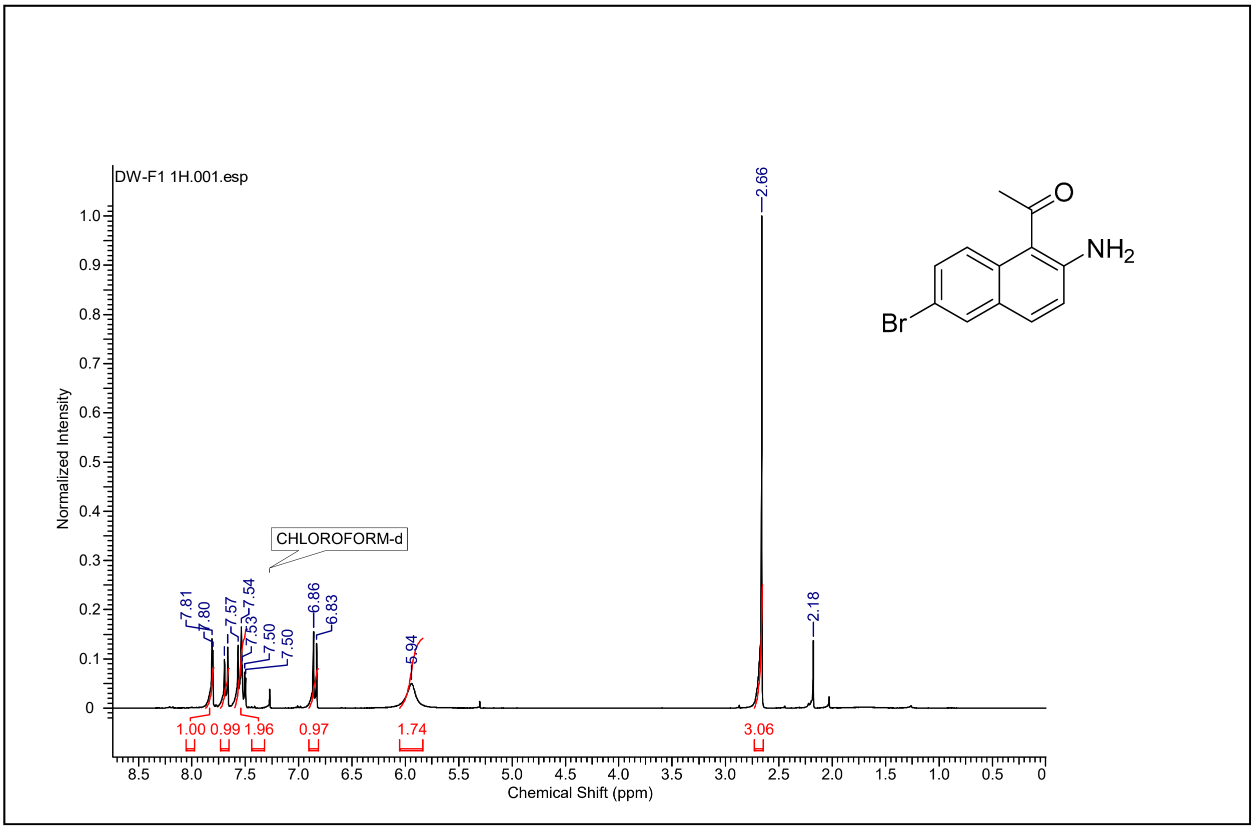


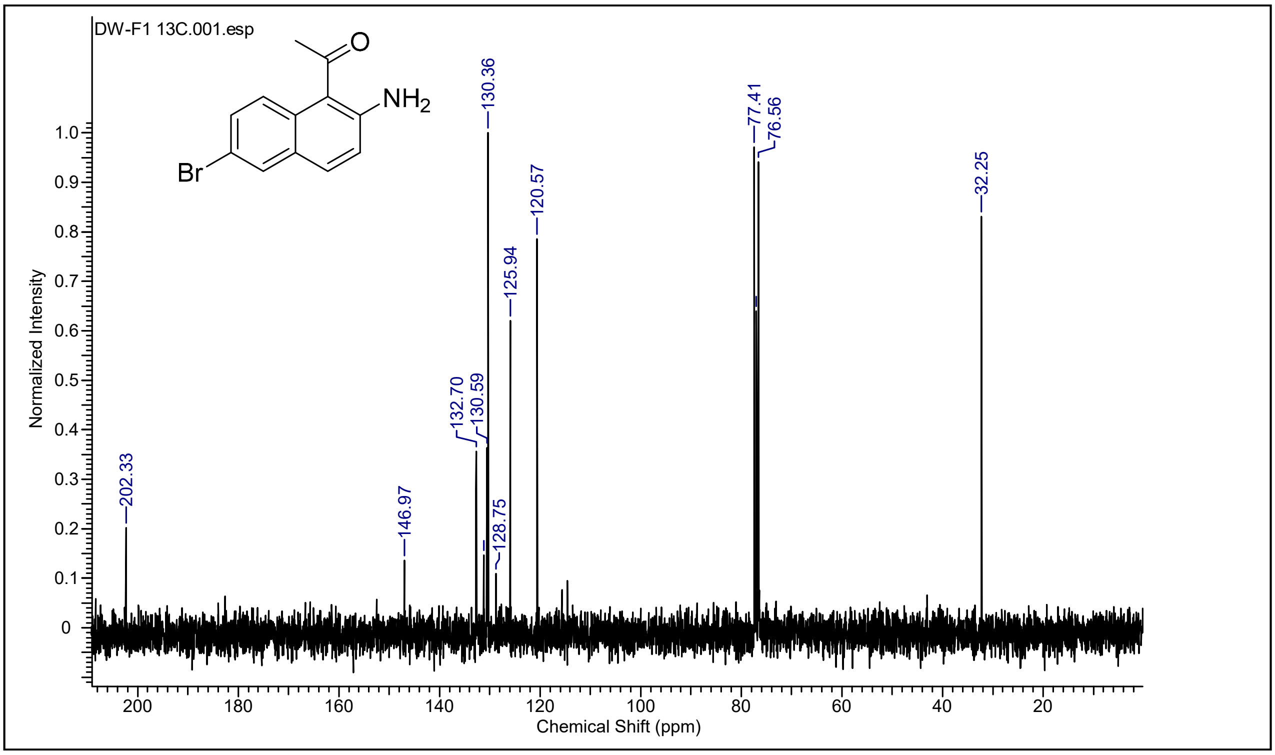


$^{1}H$ and $^{13}C$ NMR spectra of compound **2g**

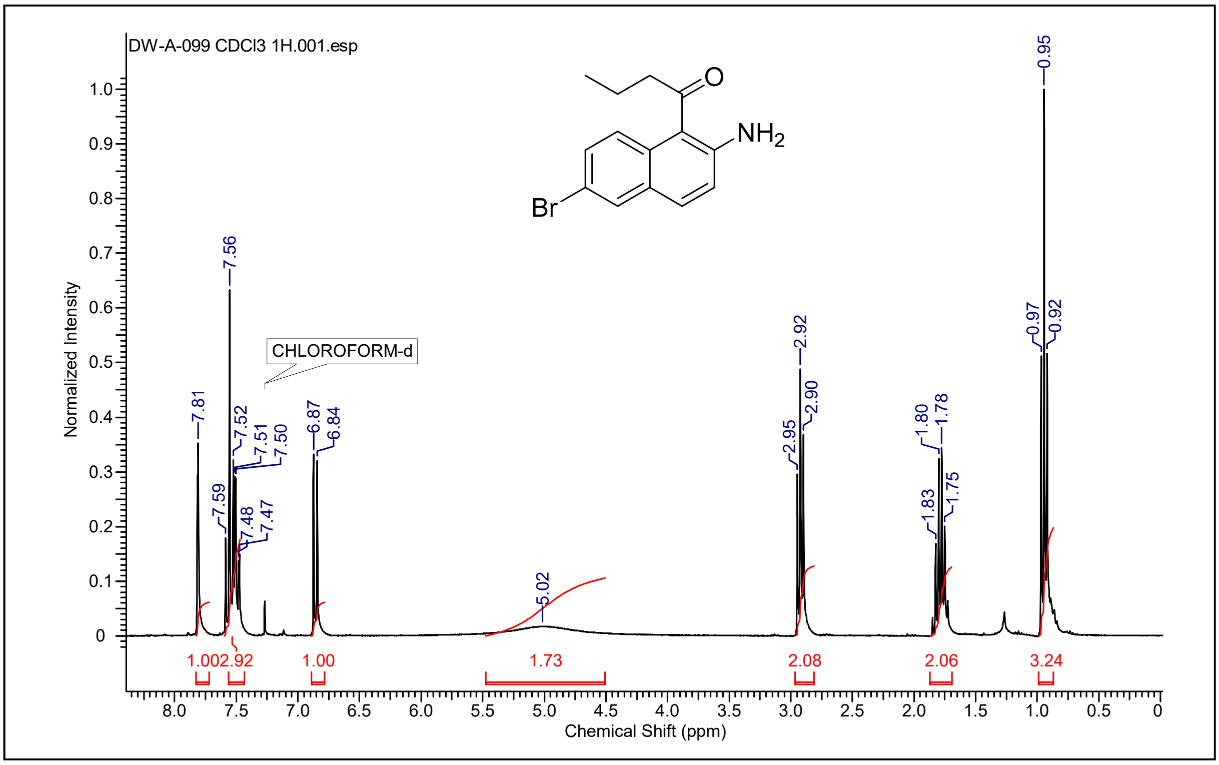


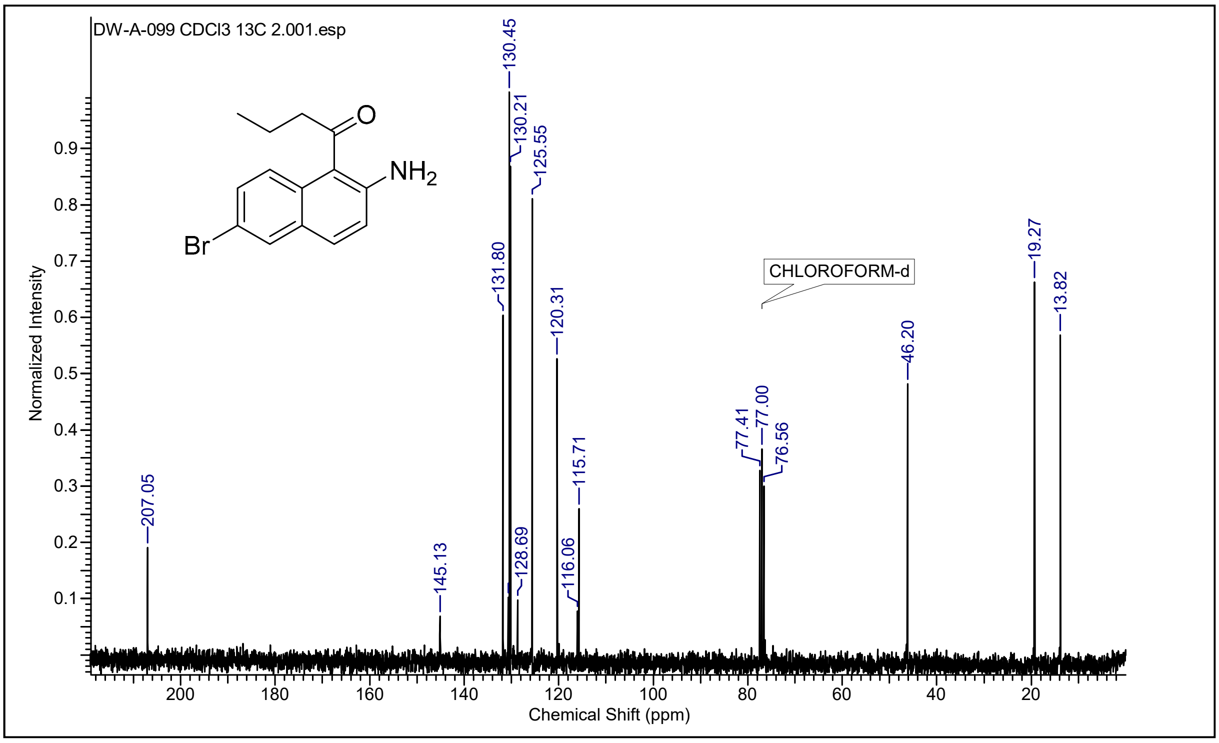


$^{1}H$ and $^{13}C$ NMR spectra of compound **2h**

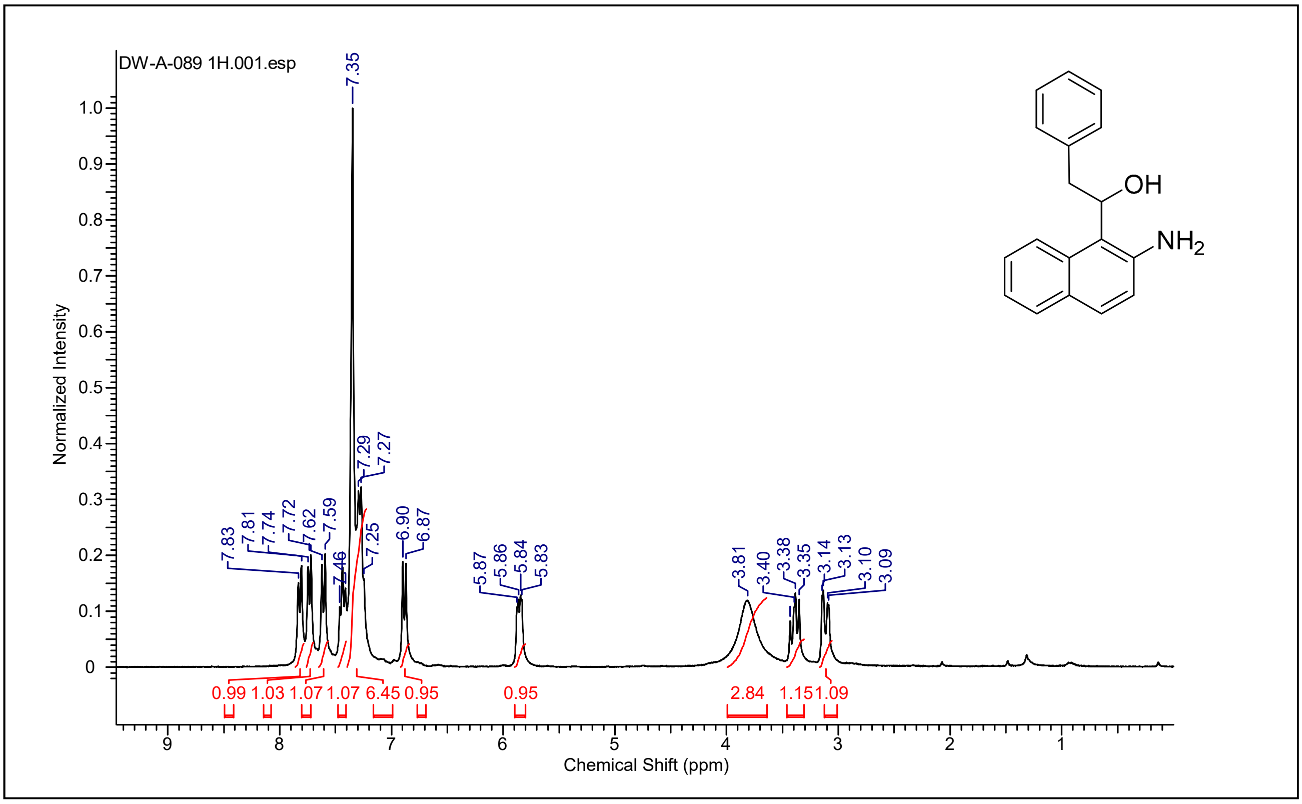


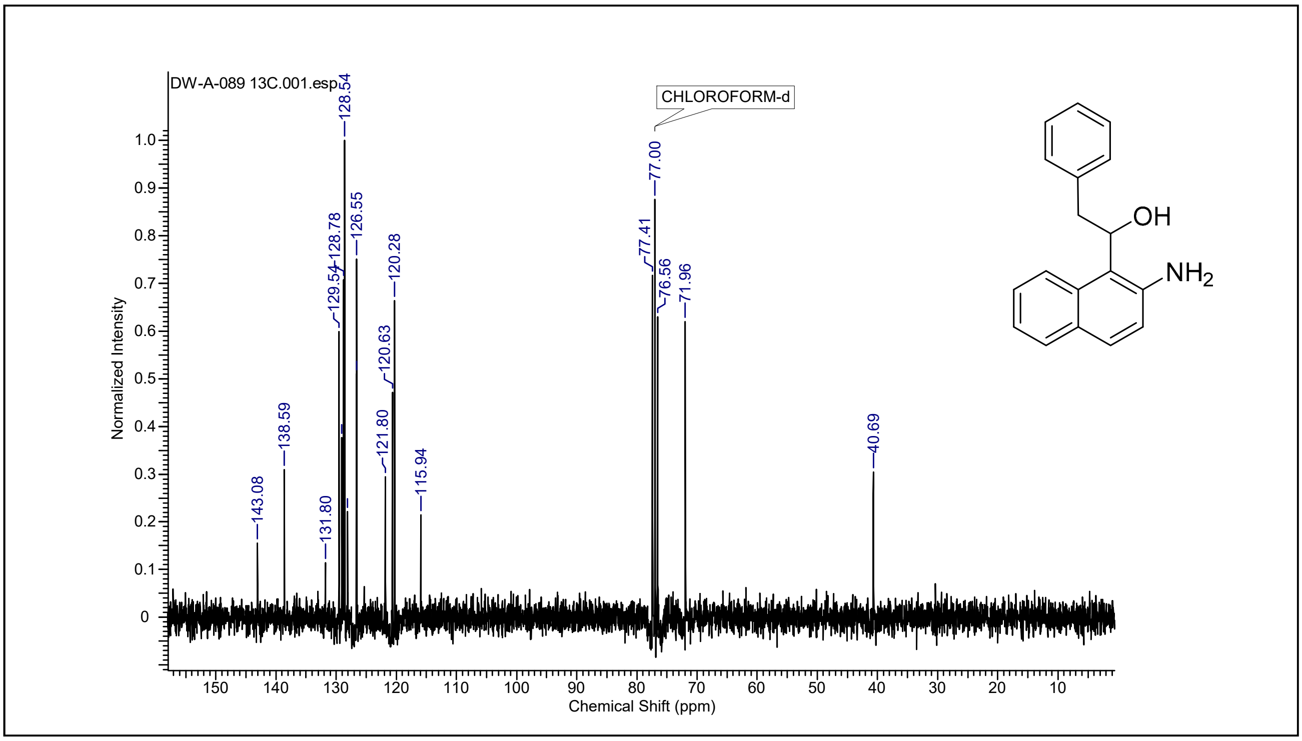


$^{1}$H and $^{13}$C NMR spectra of compound **3a**

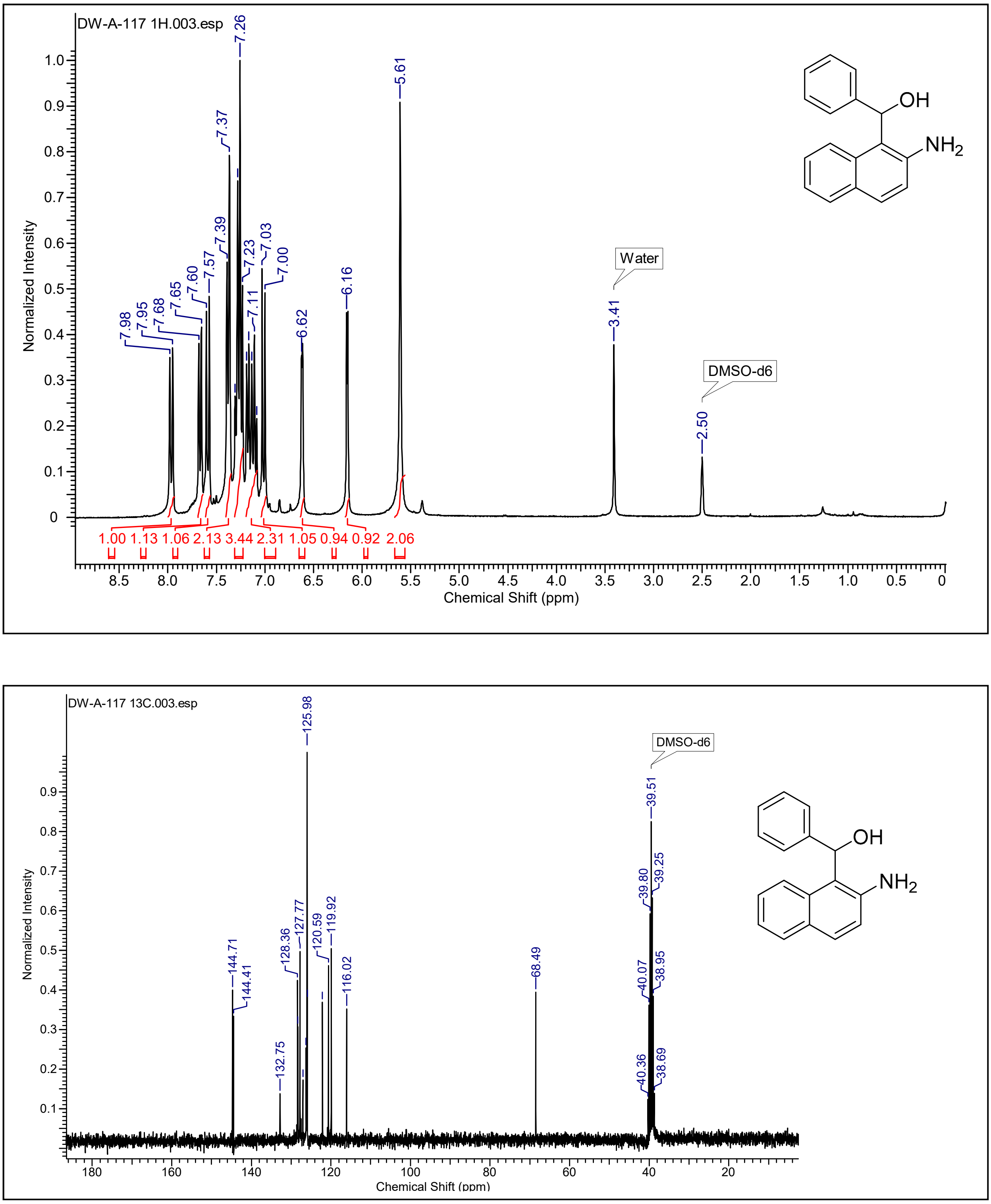


$^1$H and $^{13}$C NMR spectra of compound **3b**

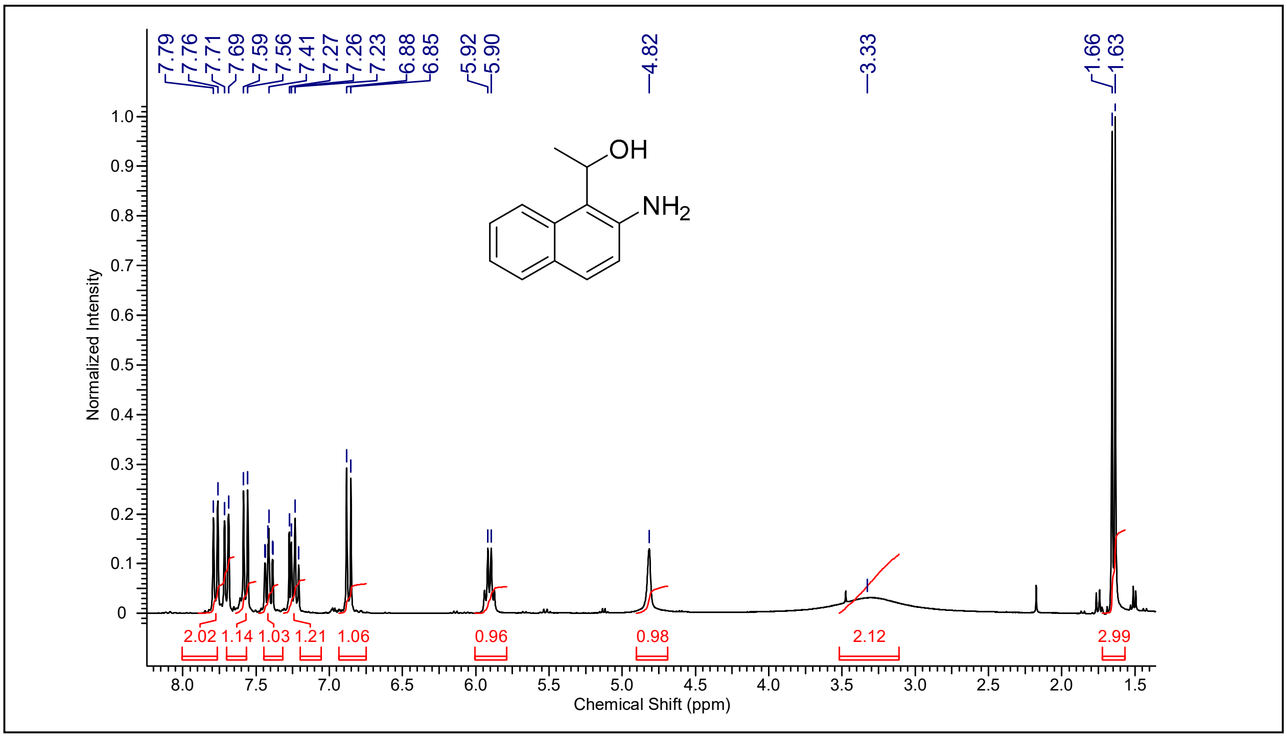


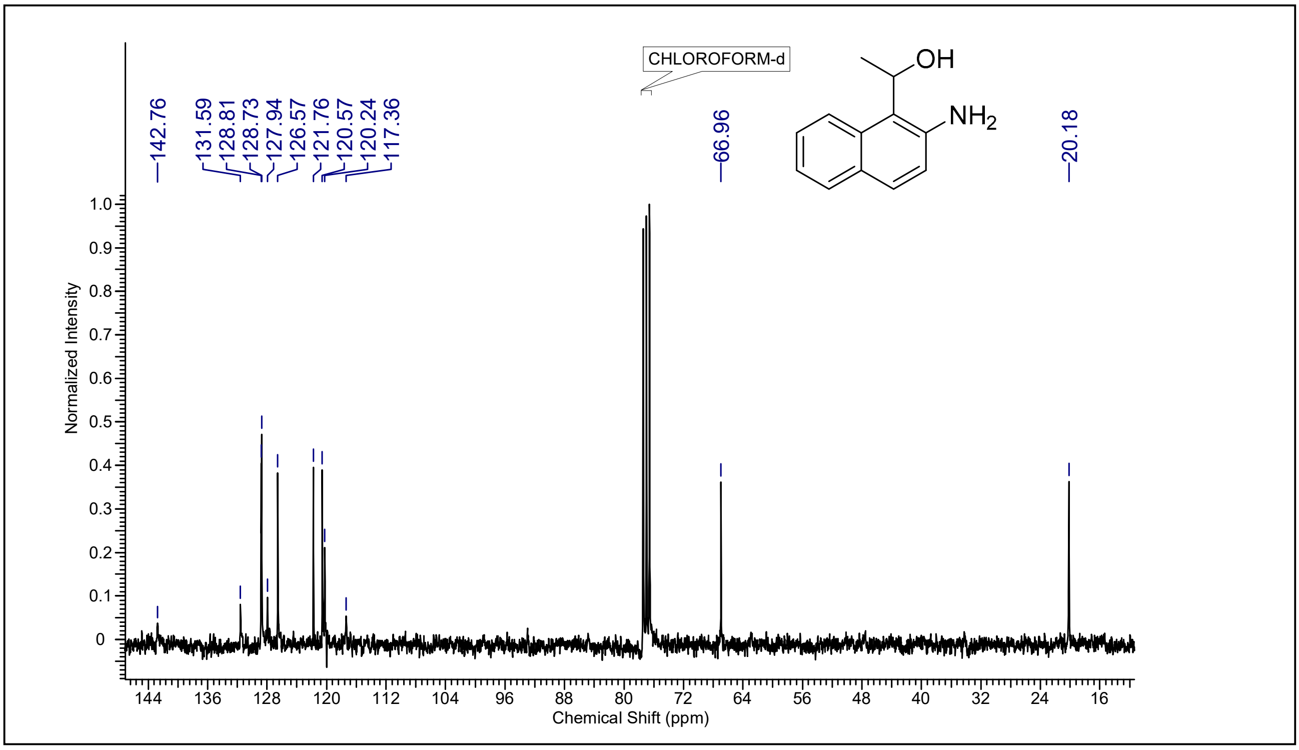


$^{1}$H and $^{13}$C NMR spectra of compound **3c**

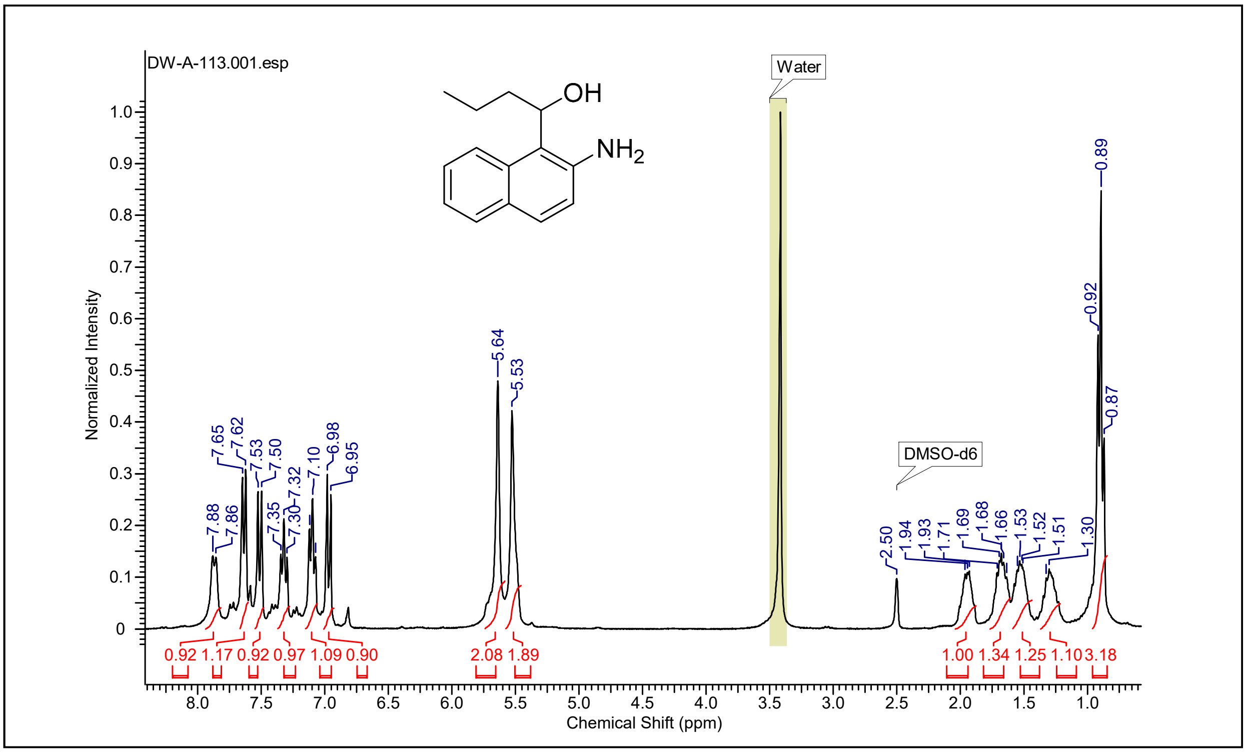


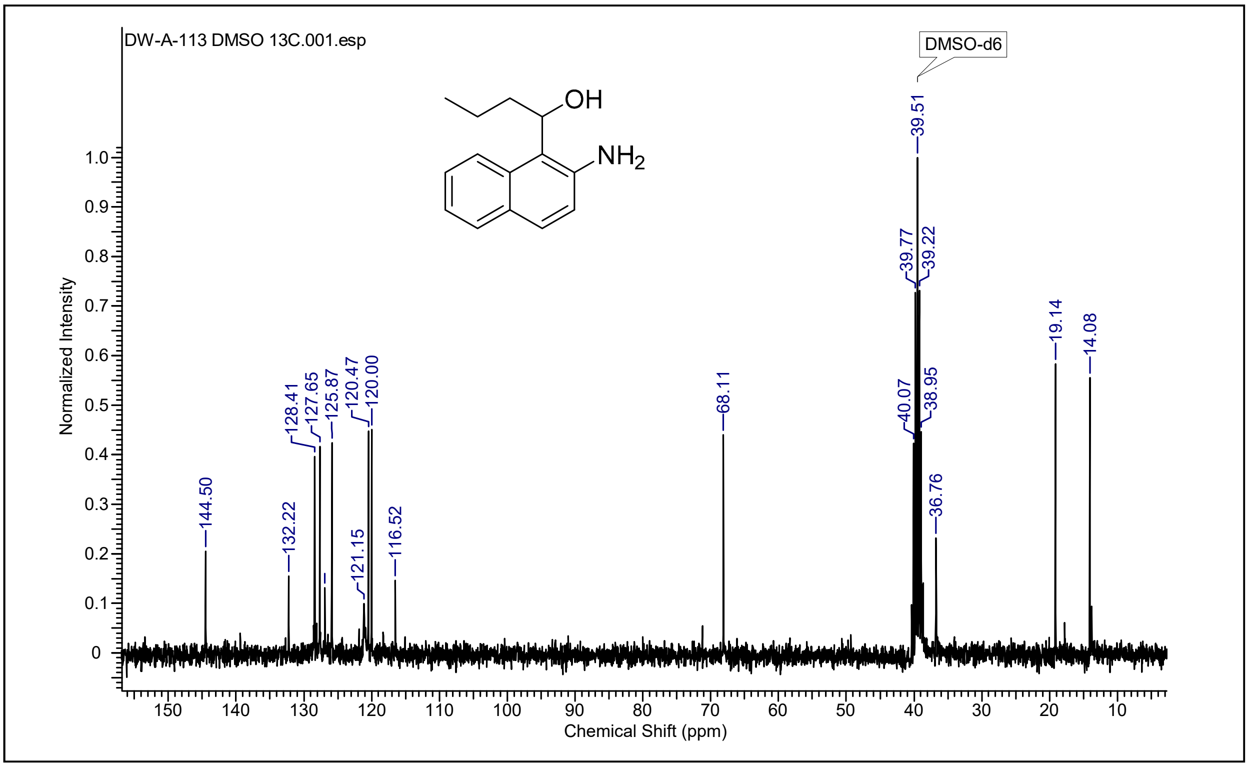


$^{1}H$ and $^{13}C$ NMR spectra of compound **3d**

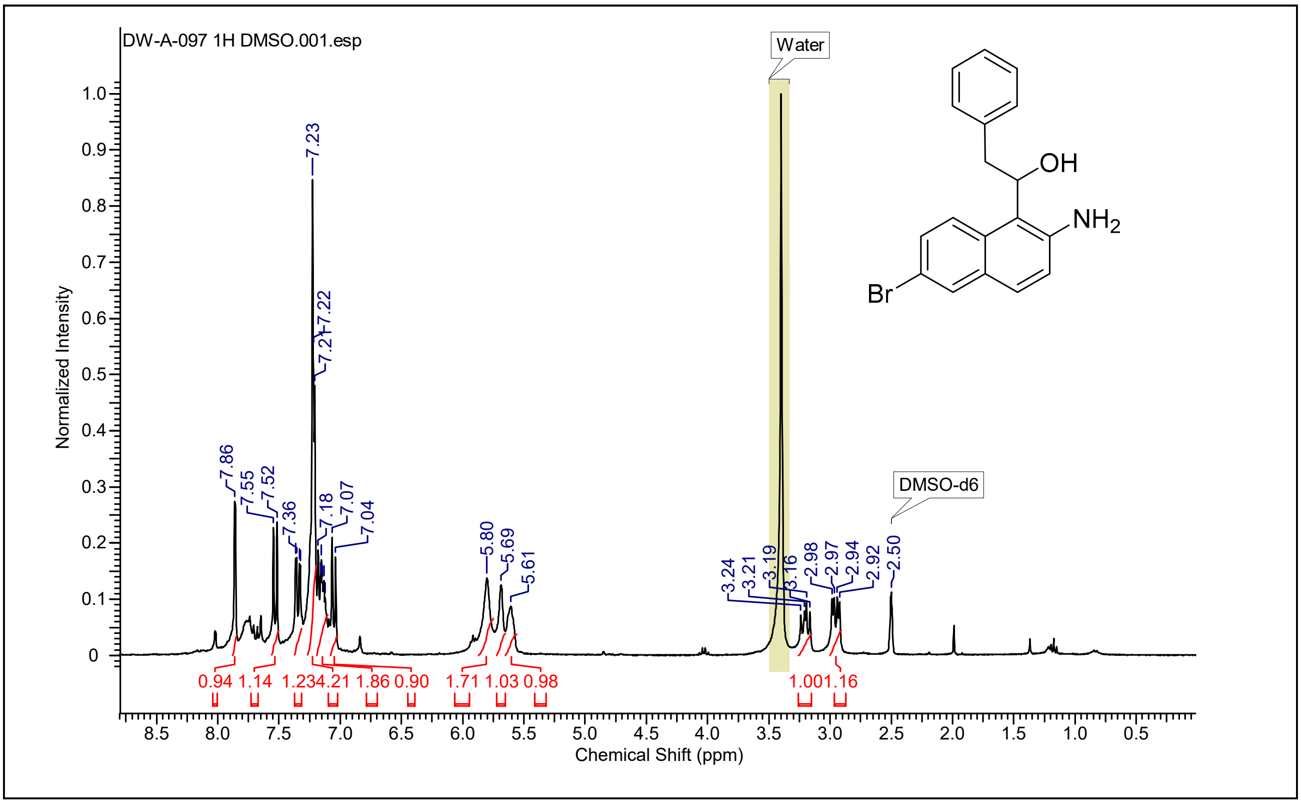


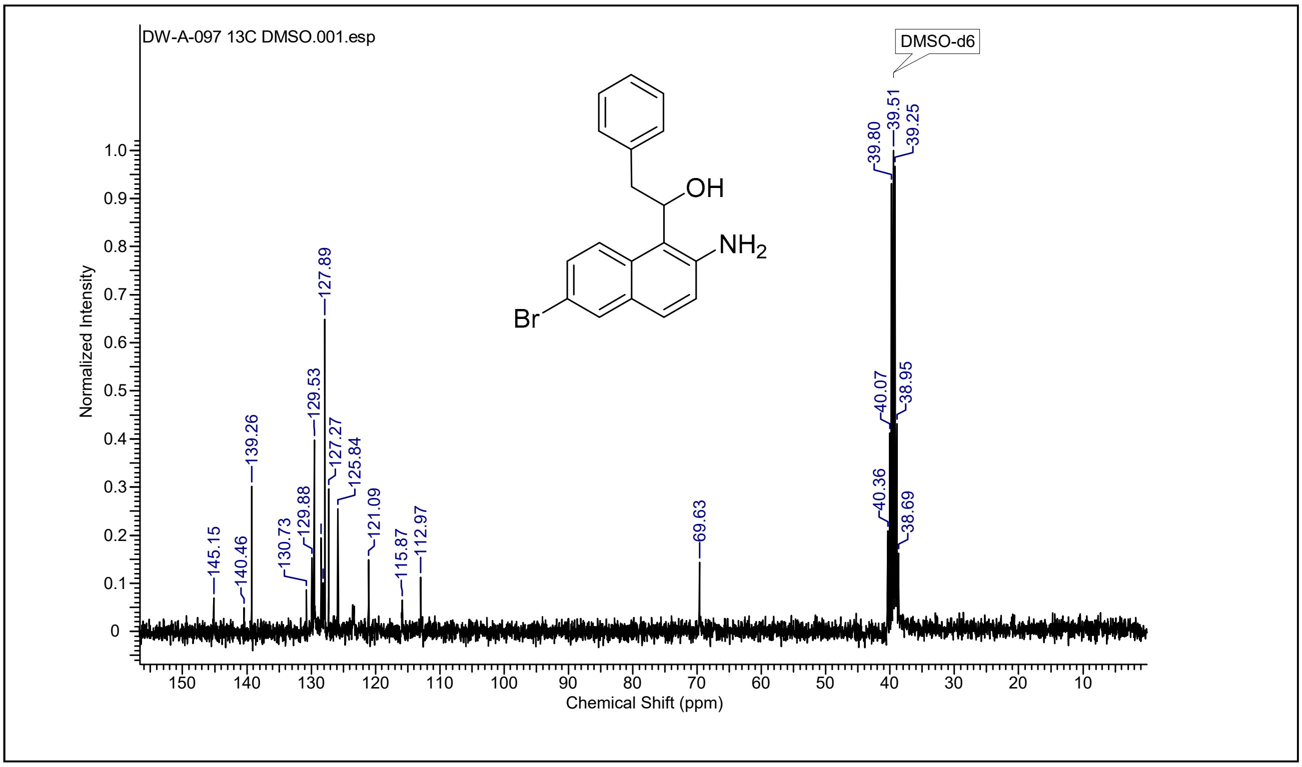


$^1H$ and $^{13}C$ NMR spectra of compound **3e**

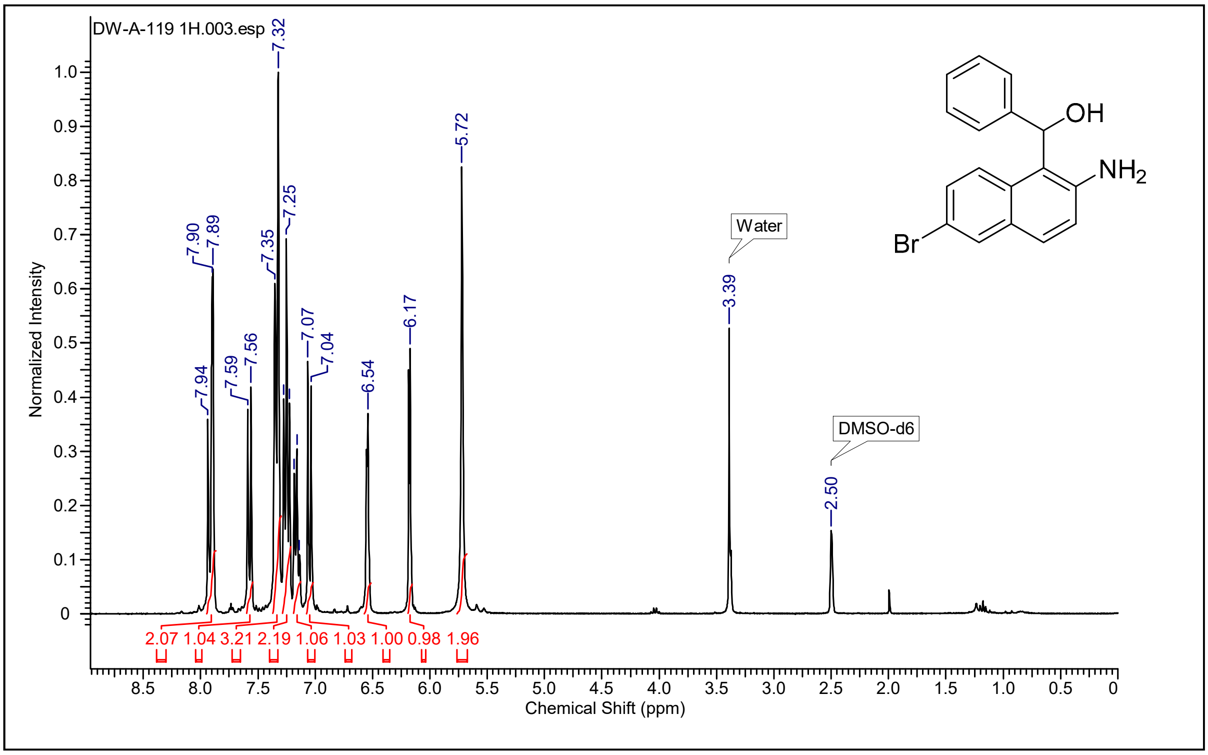


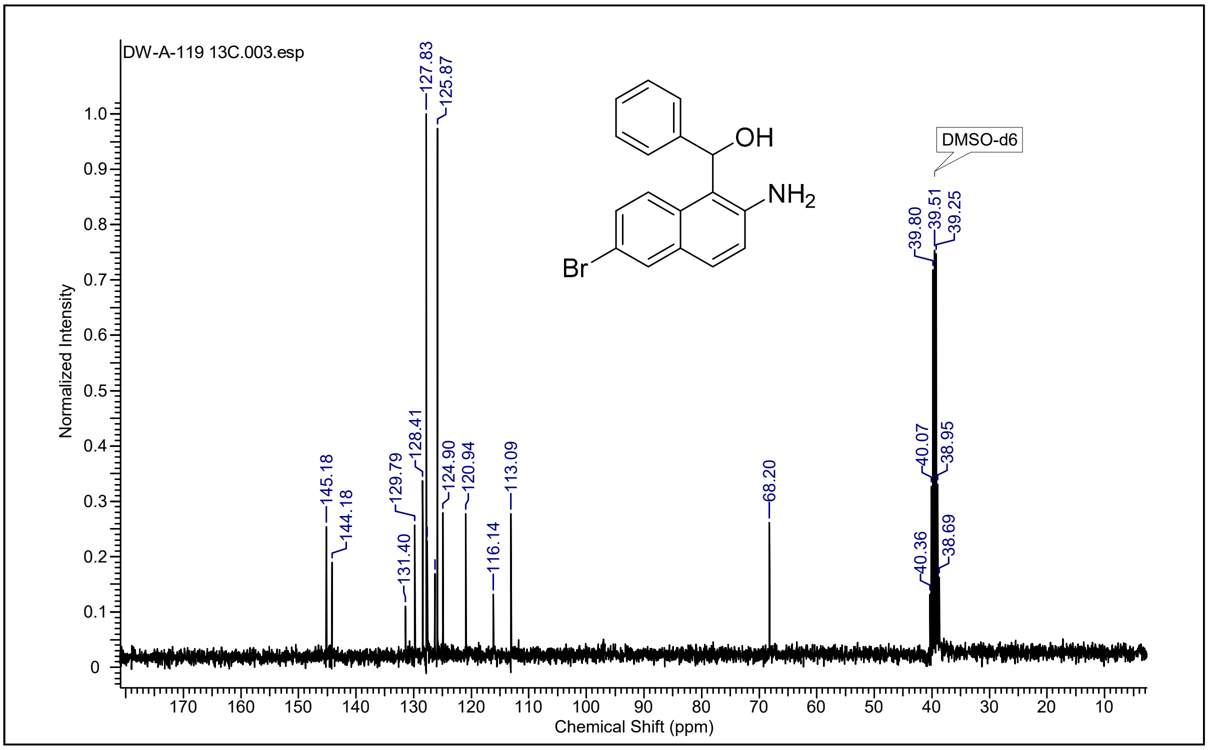


$^{1}H$ and $^{13}C$ NMR spectra of compound **3f**

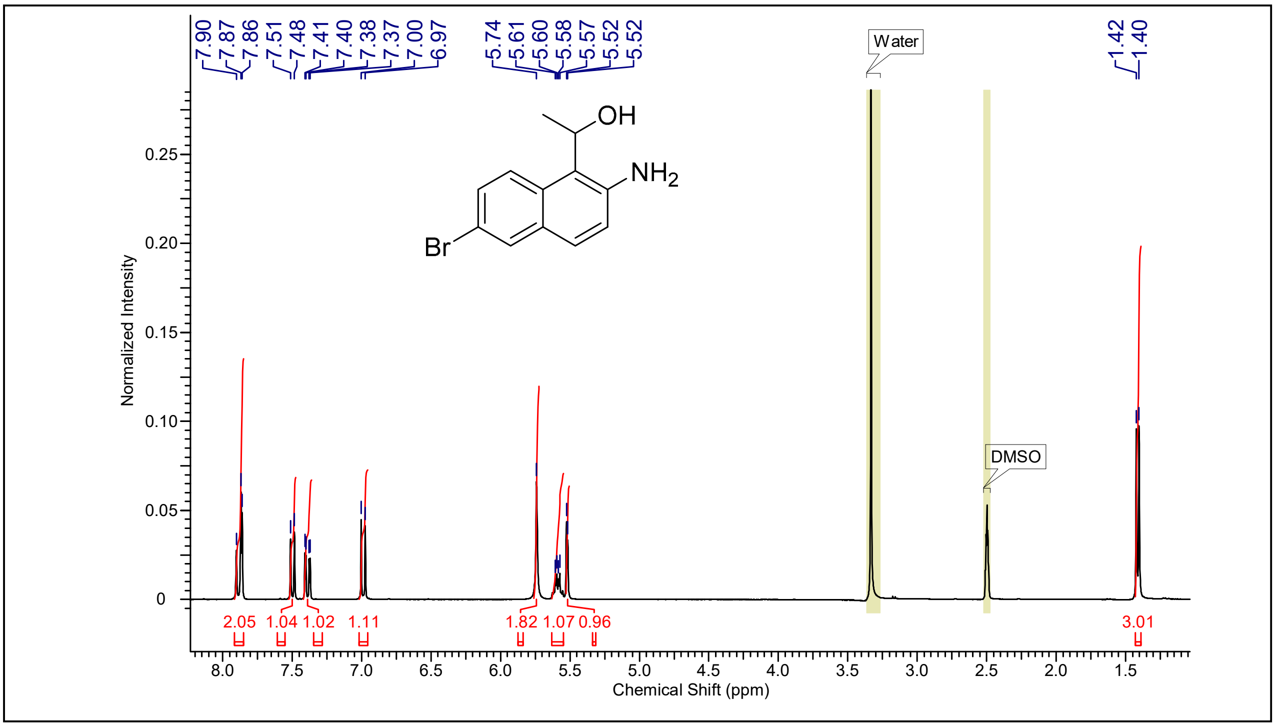


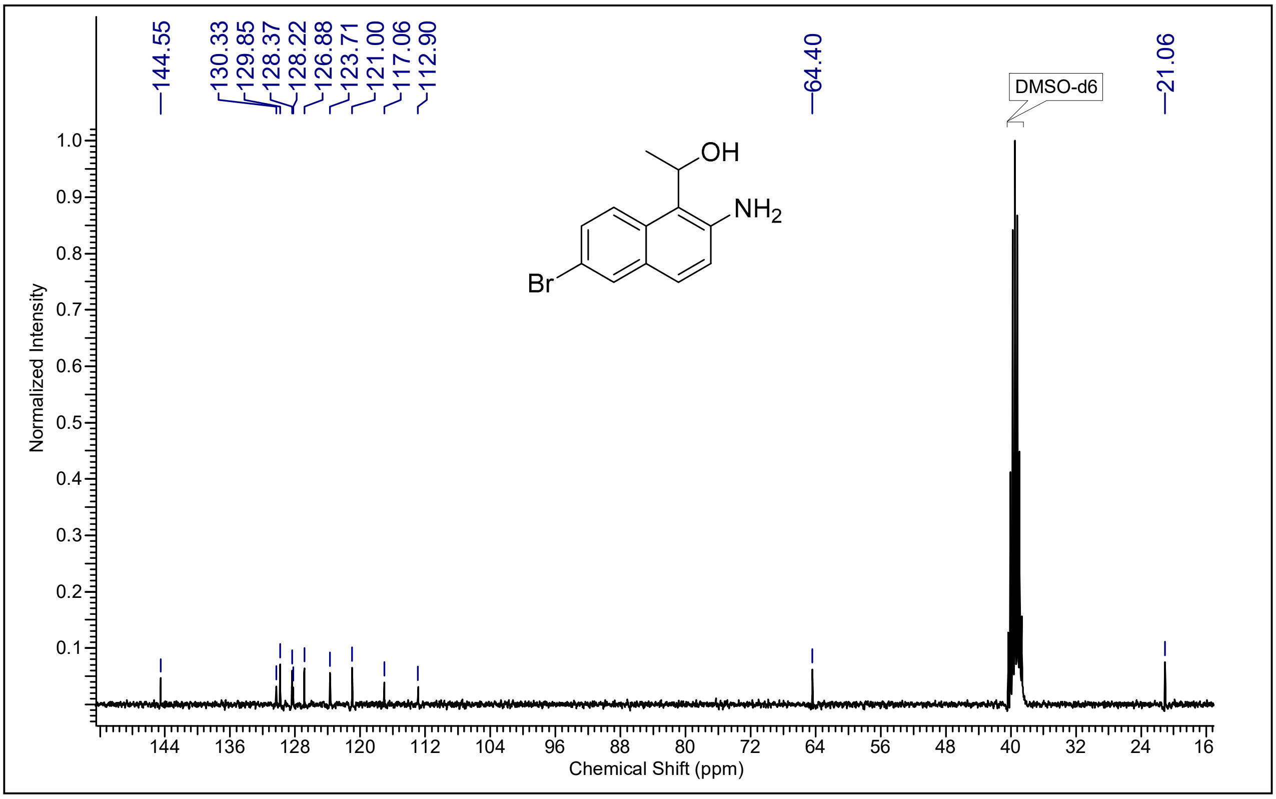


$^{1}$H and $^{13}$C NMR spectra of compound **3g**

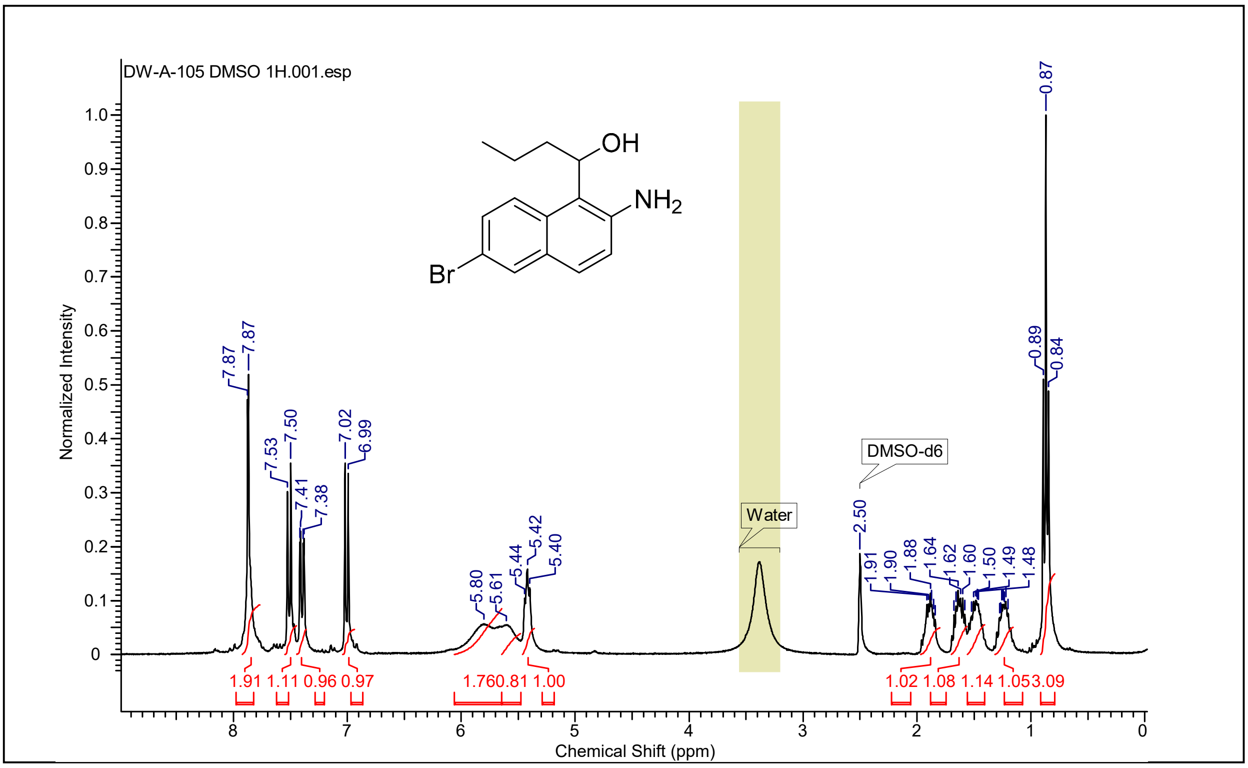


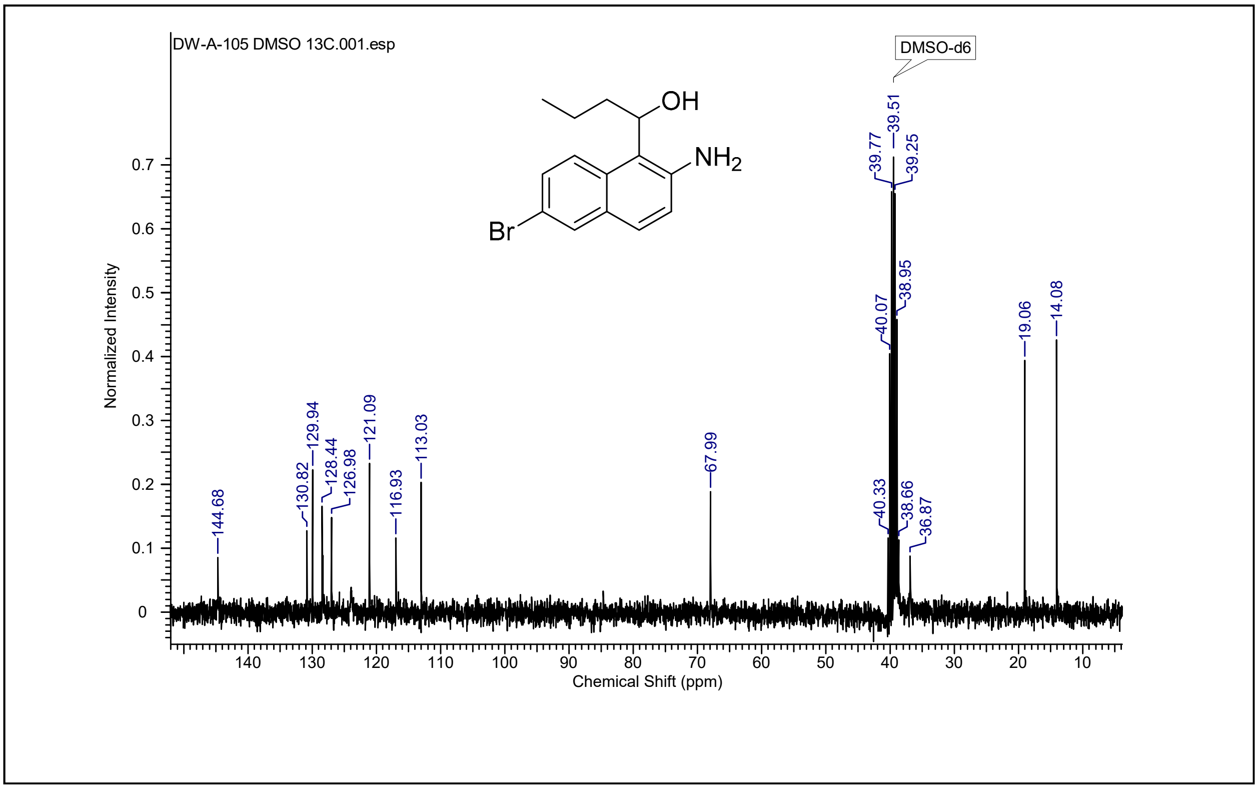


$^1$H and $^{13}$C NMR spectra of compound **3h**

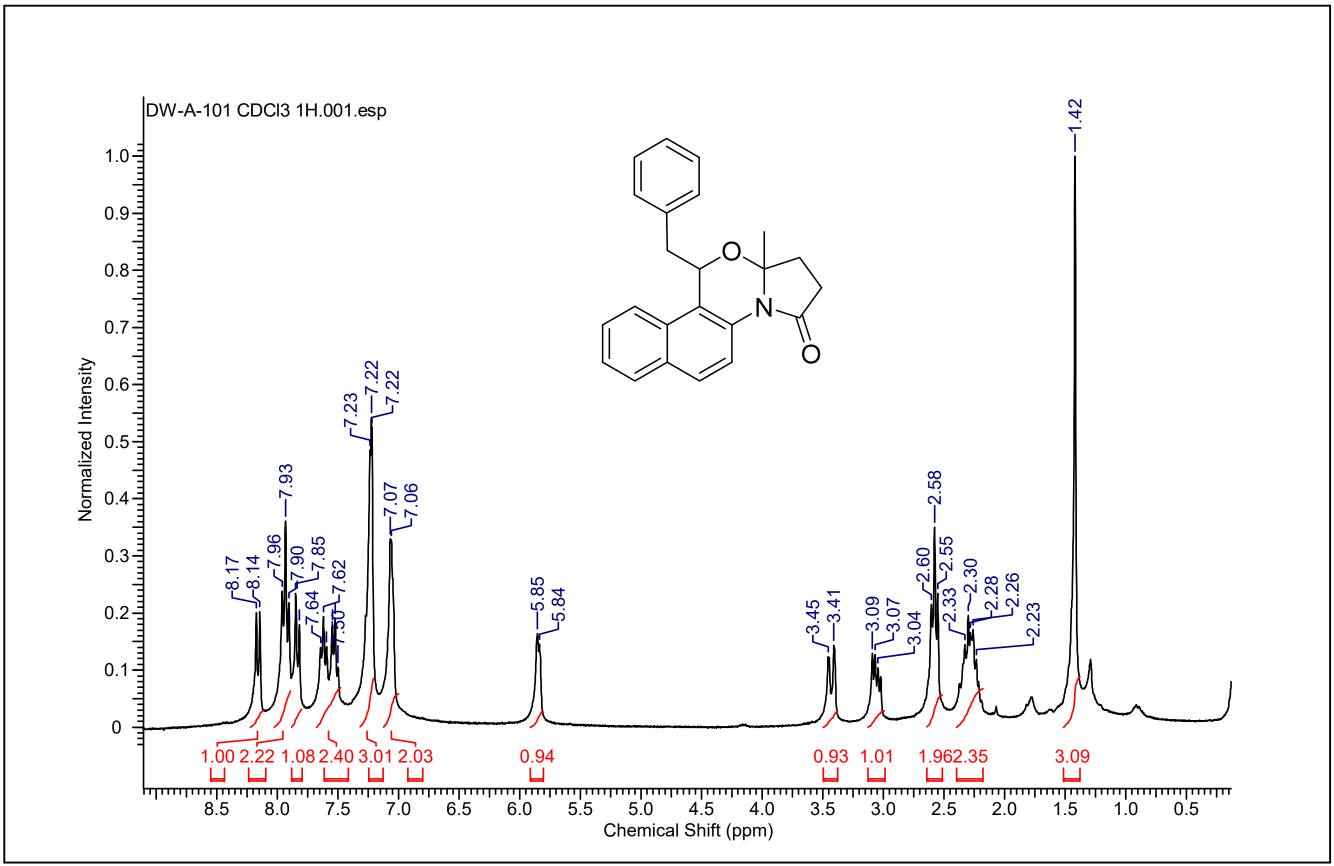


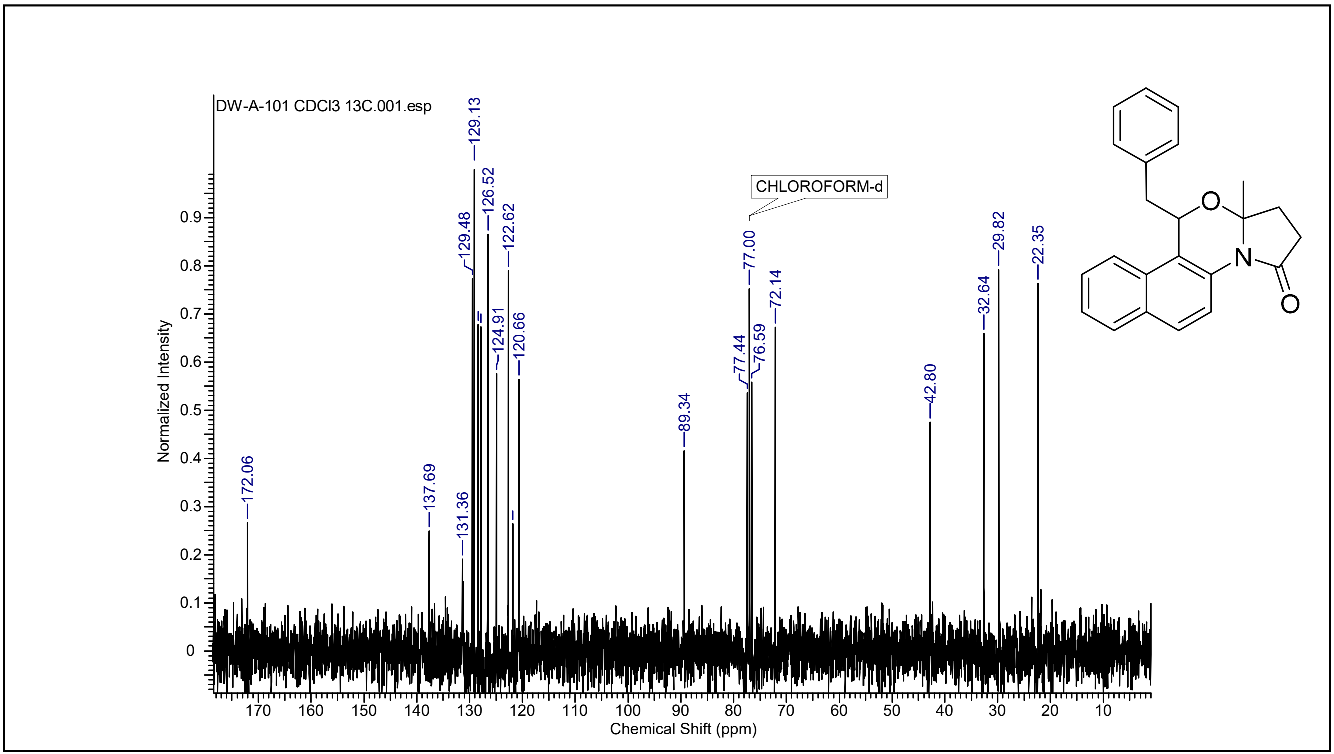


$^{1}$H and $^{13}$C NMR spectra of compound **4a**

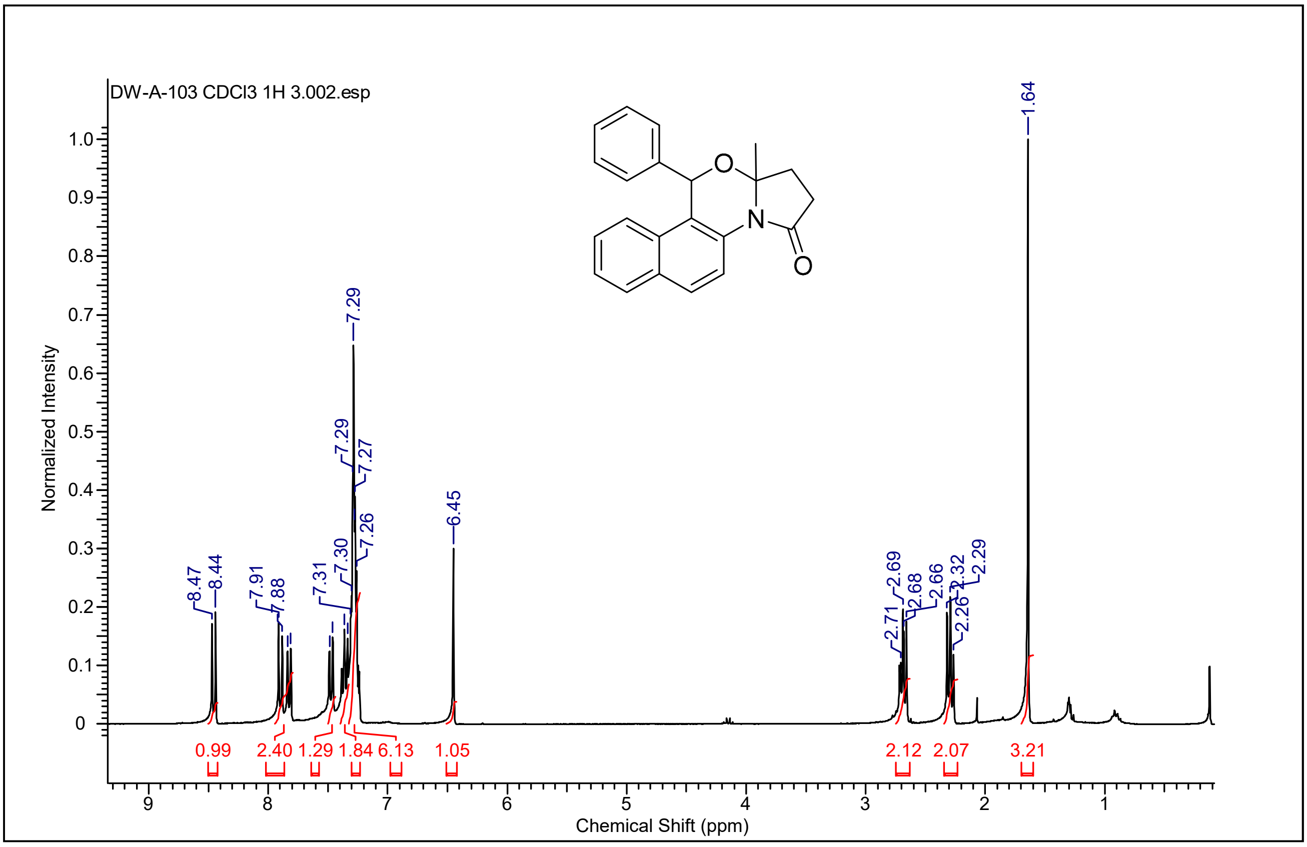


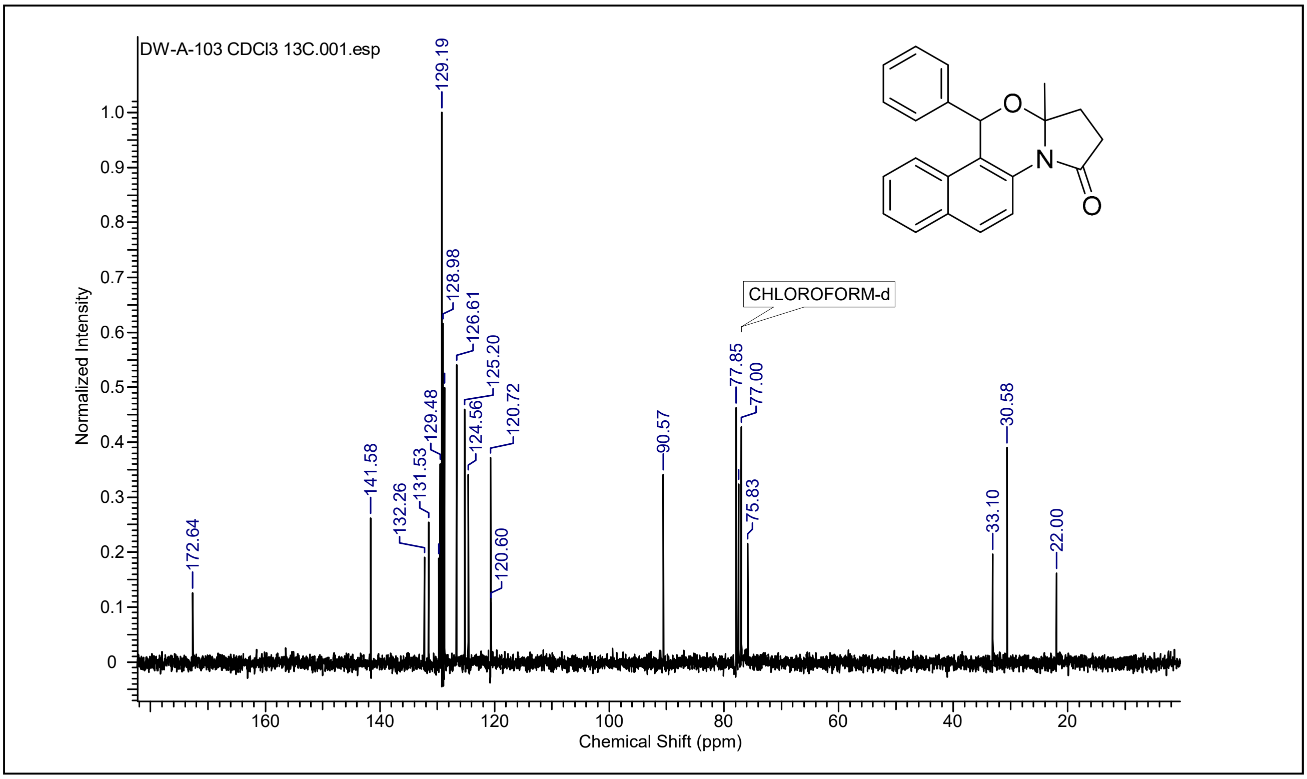


$^1$H and $^{13}$C NMR spectra of compound **4b**

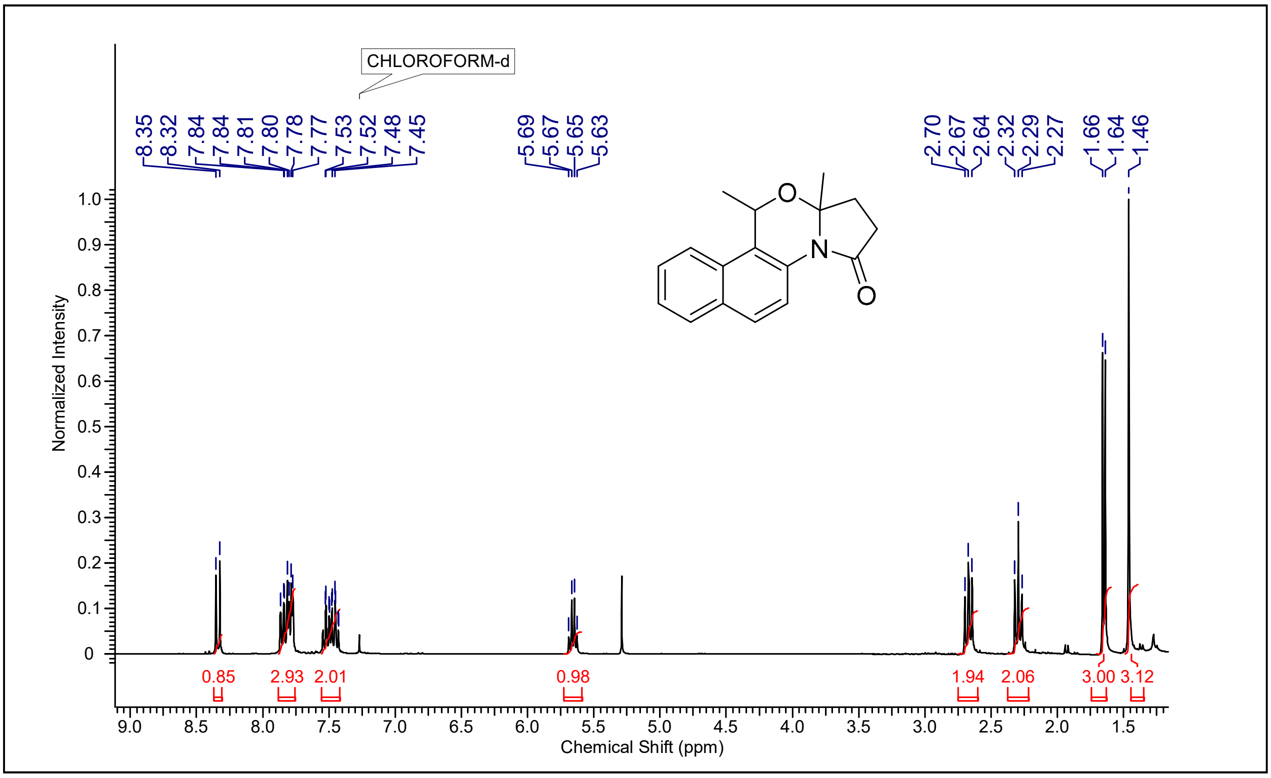


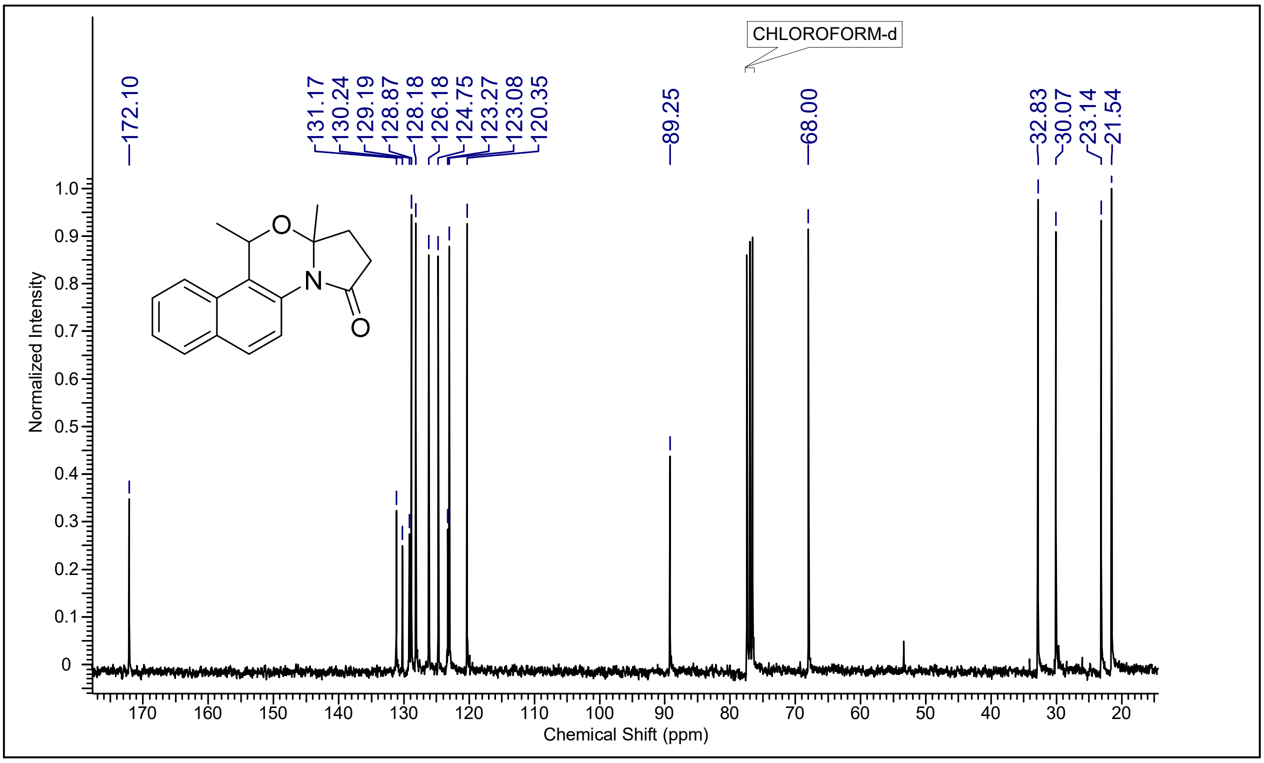


$^{1}$H and $^{13}$C NMR spectra of compound **4c**

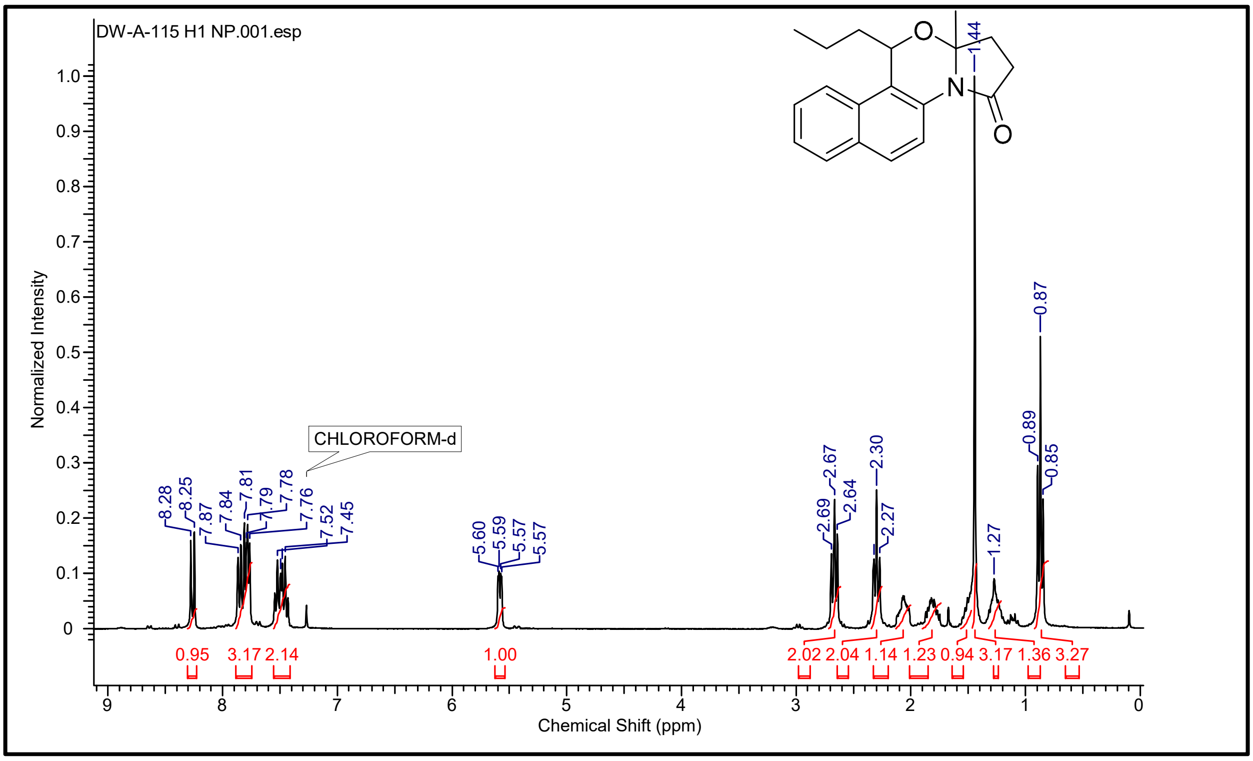


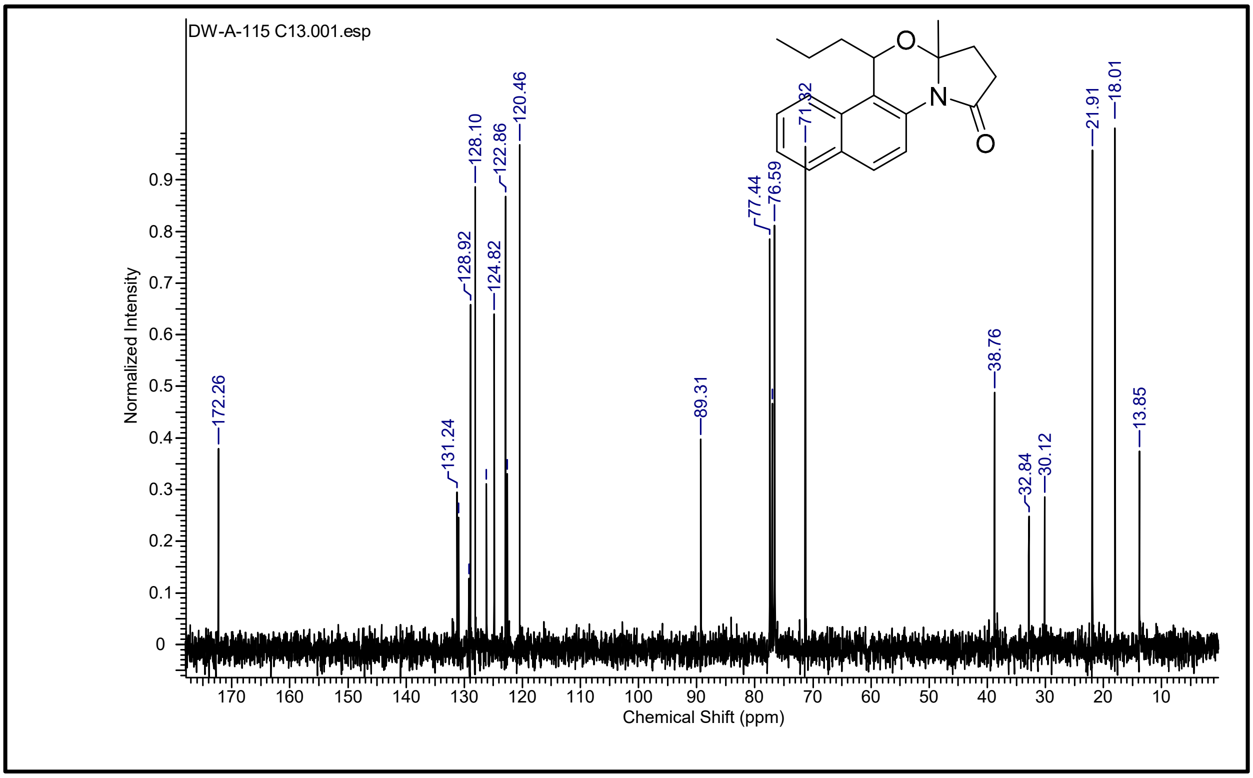


$^{1}$H and $^{13}$C NMR spectra of compound **4d**

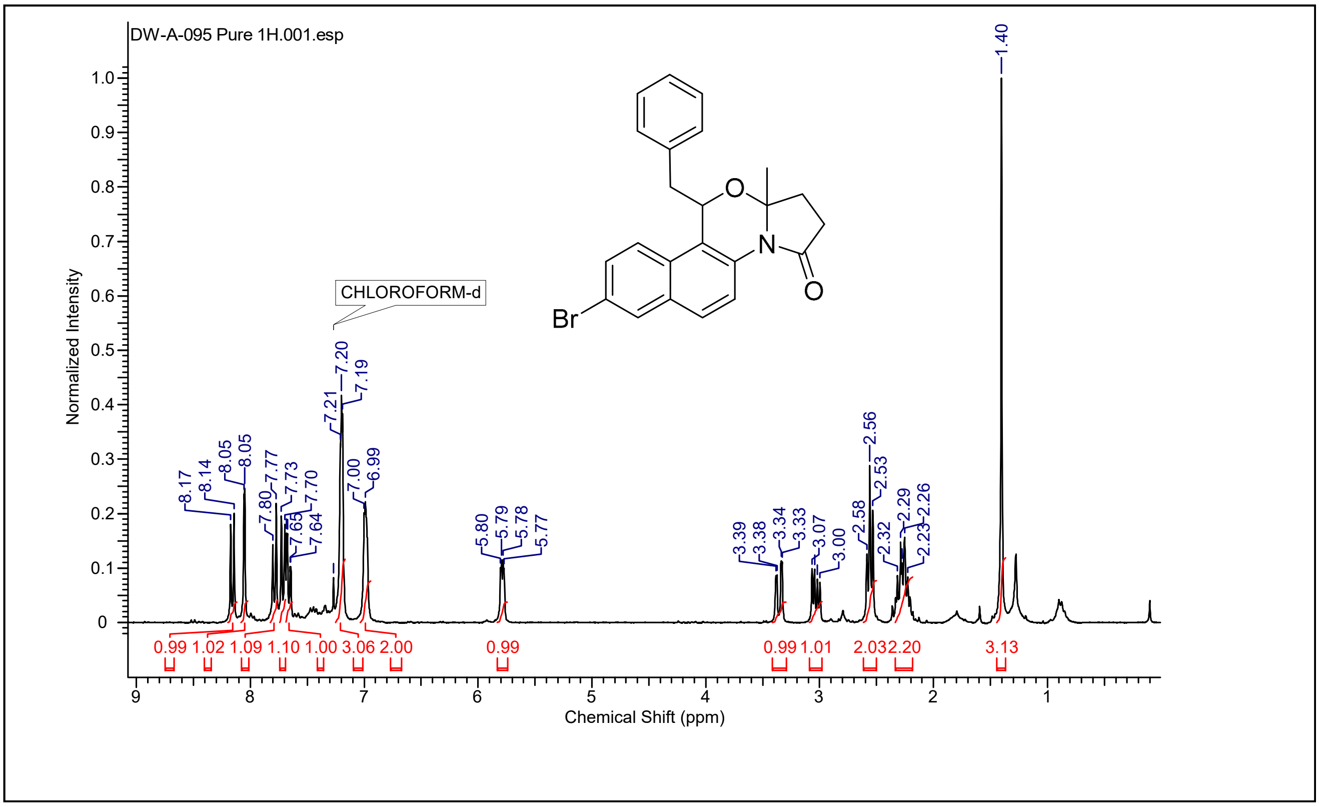


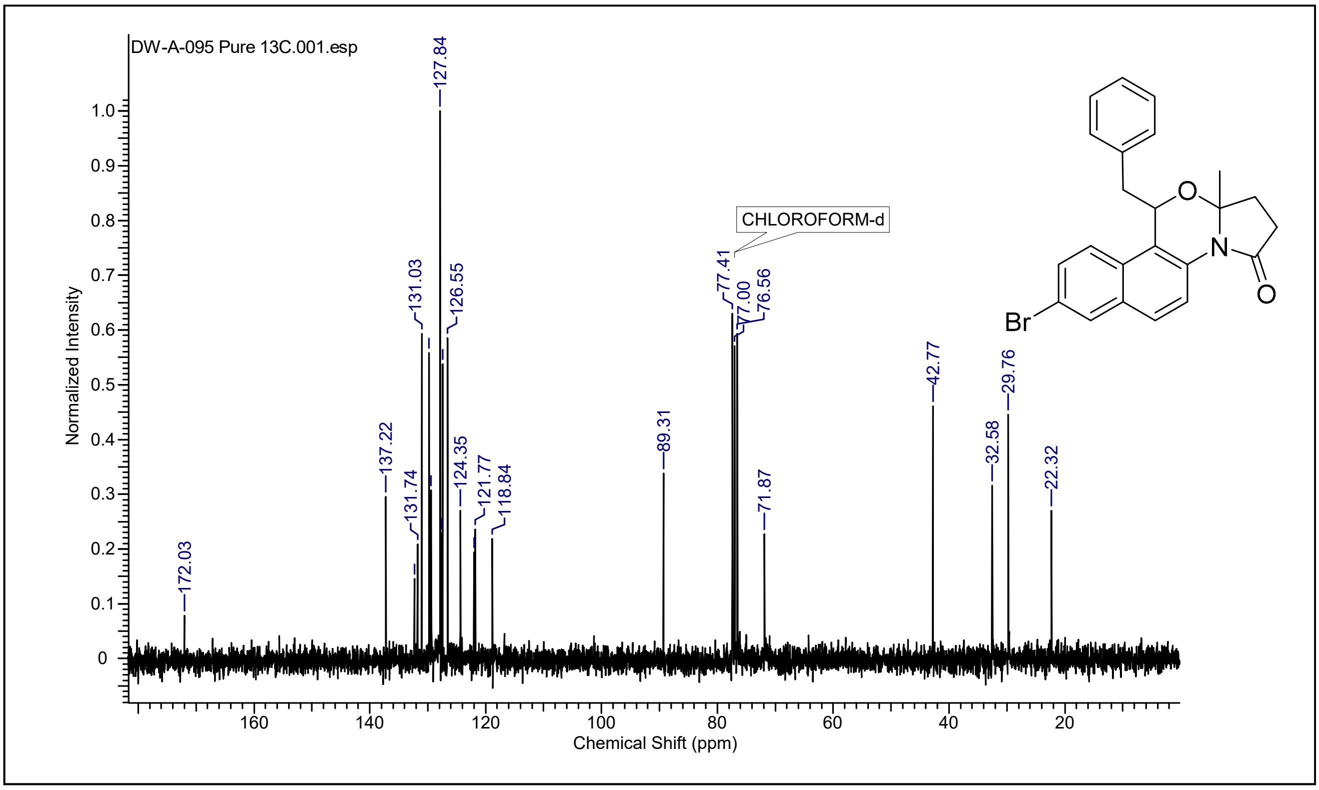


$^{1}$H and $^{13}$C NMR spectra of compound **4e**

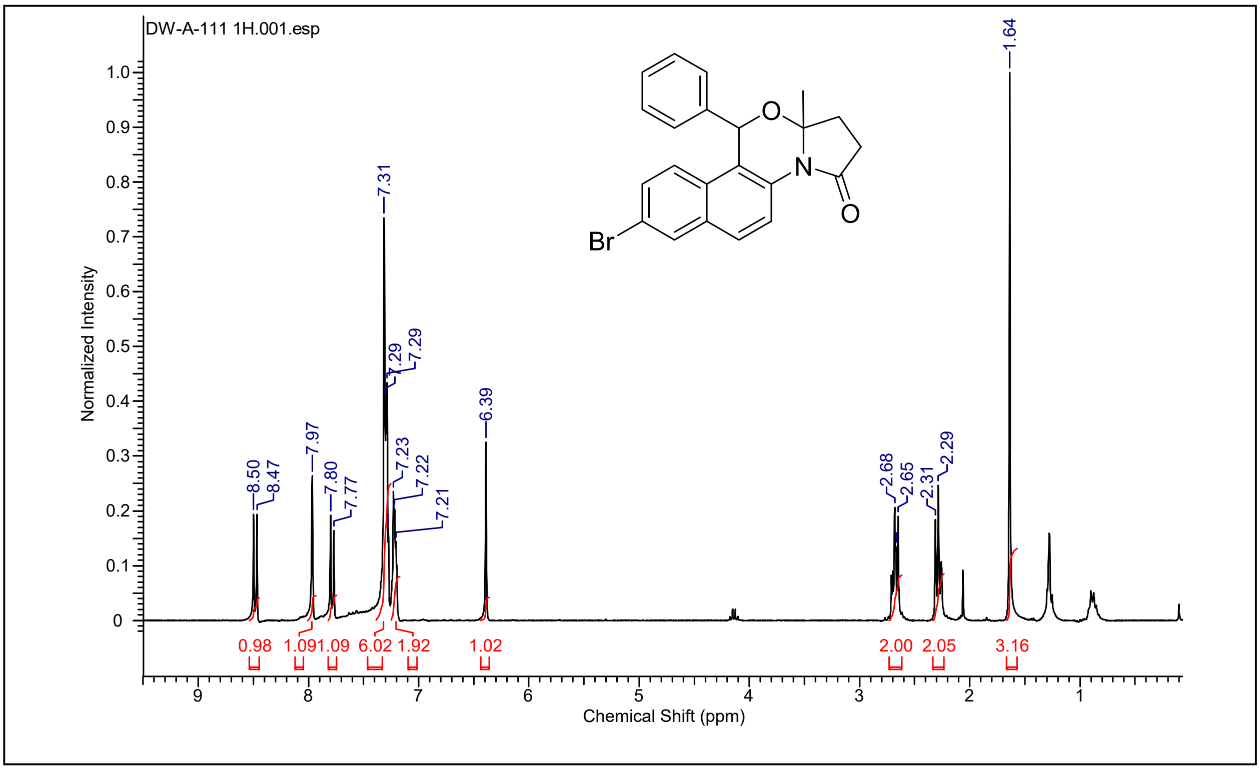


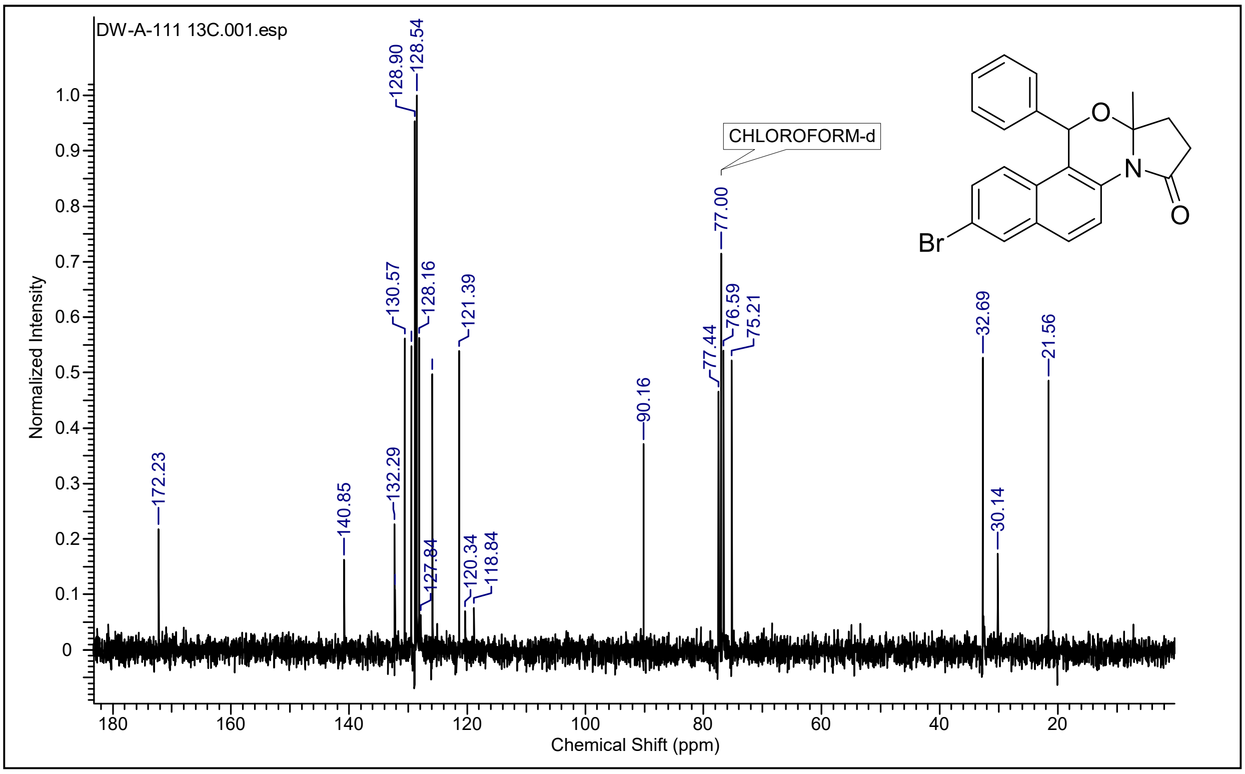


$^{1}$H and $^{13}$C NMR spectra of compound **4f**

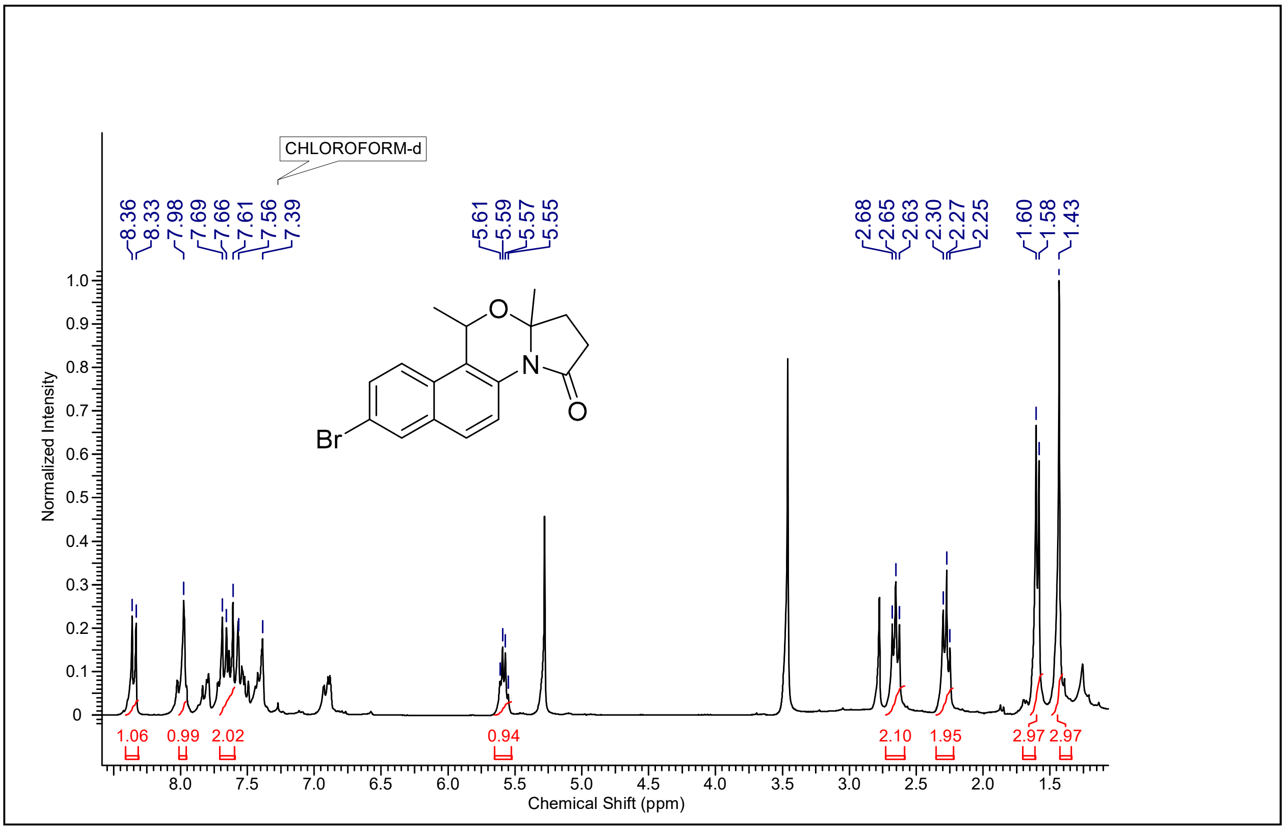


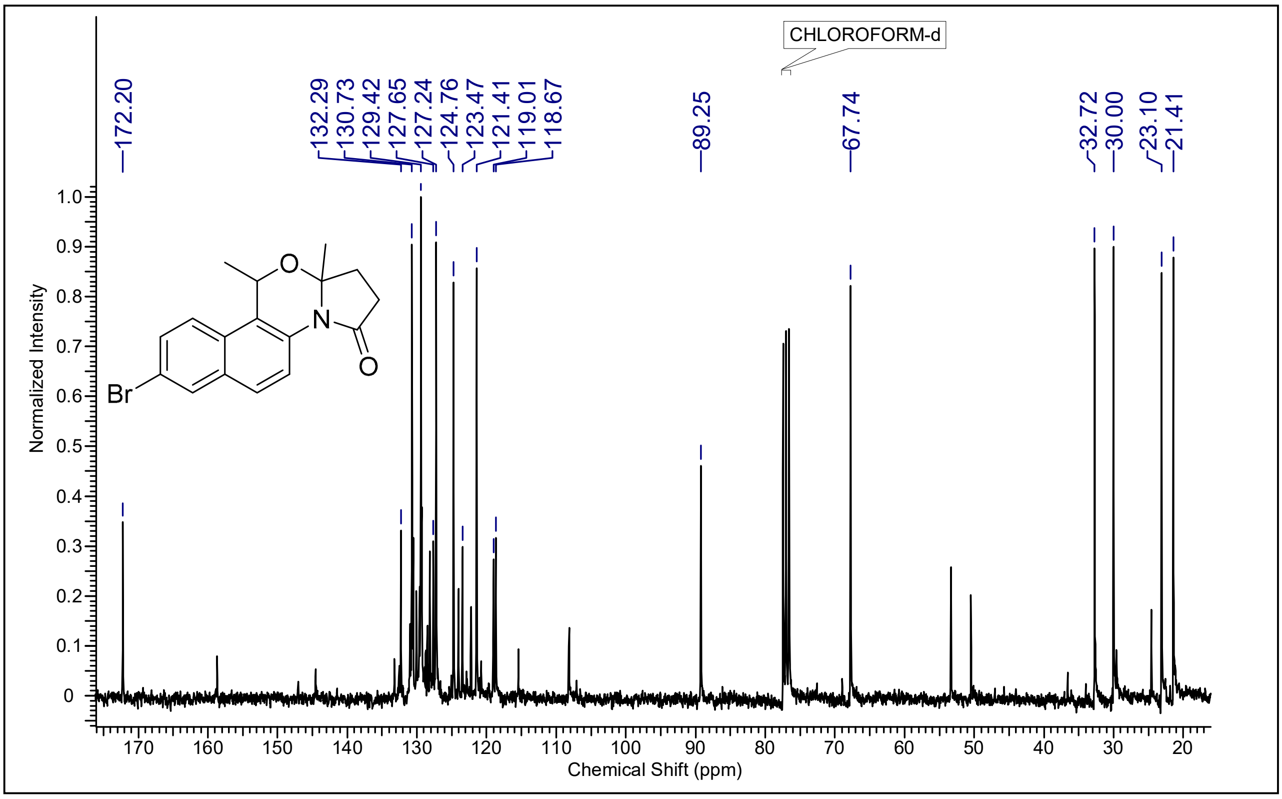


$^{1}$H and $^{13}$C NMR spectra of compound **4g**

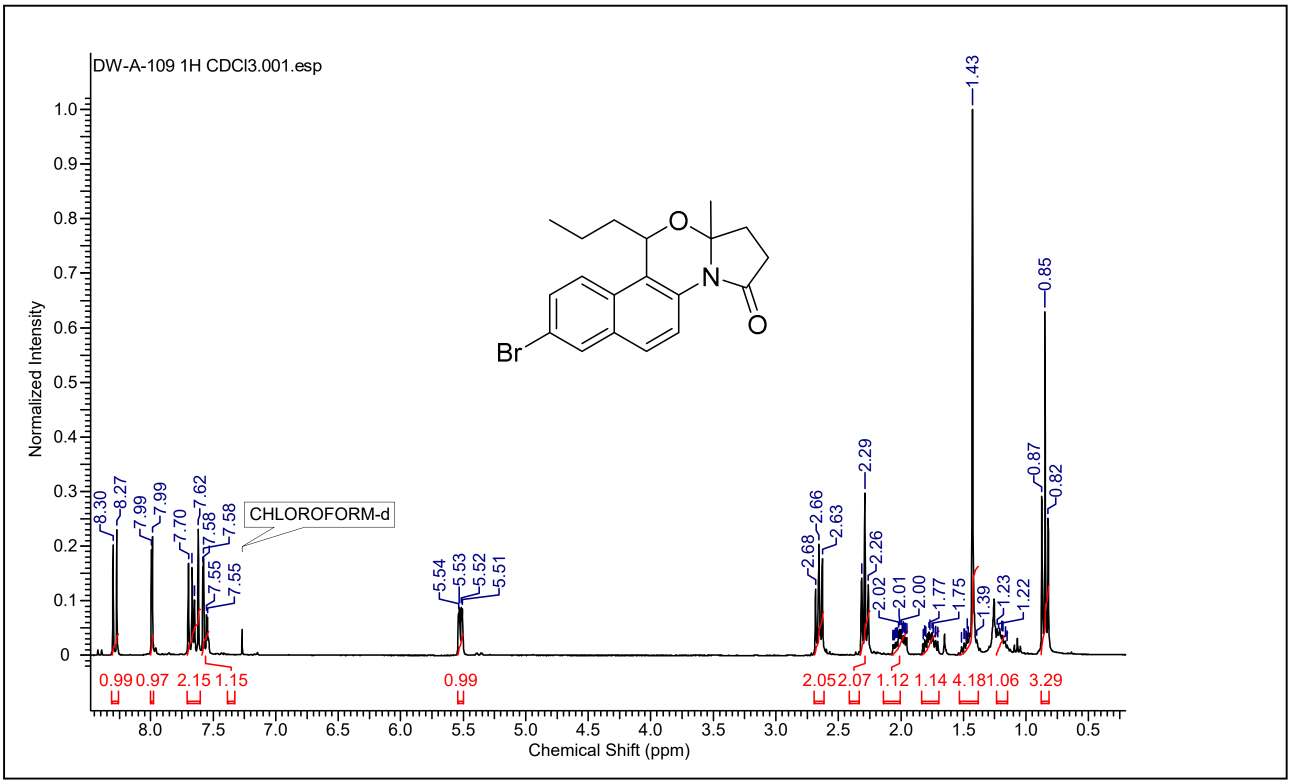


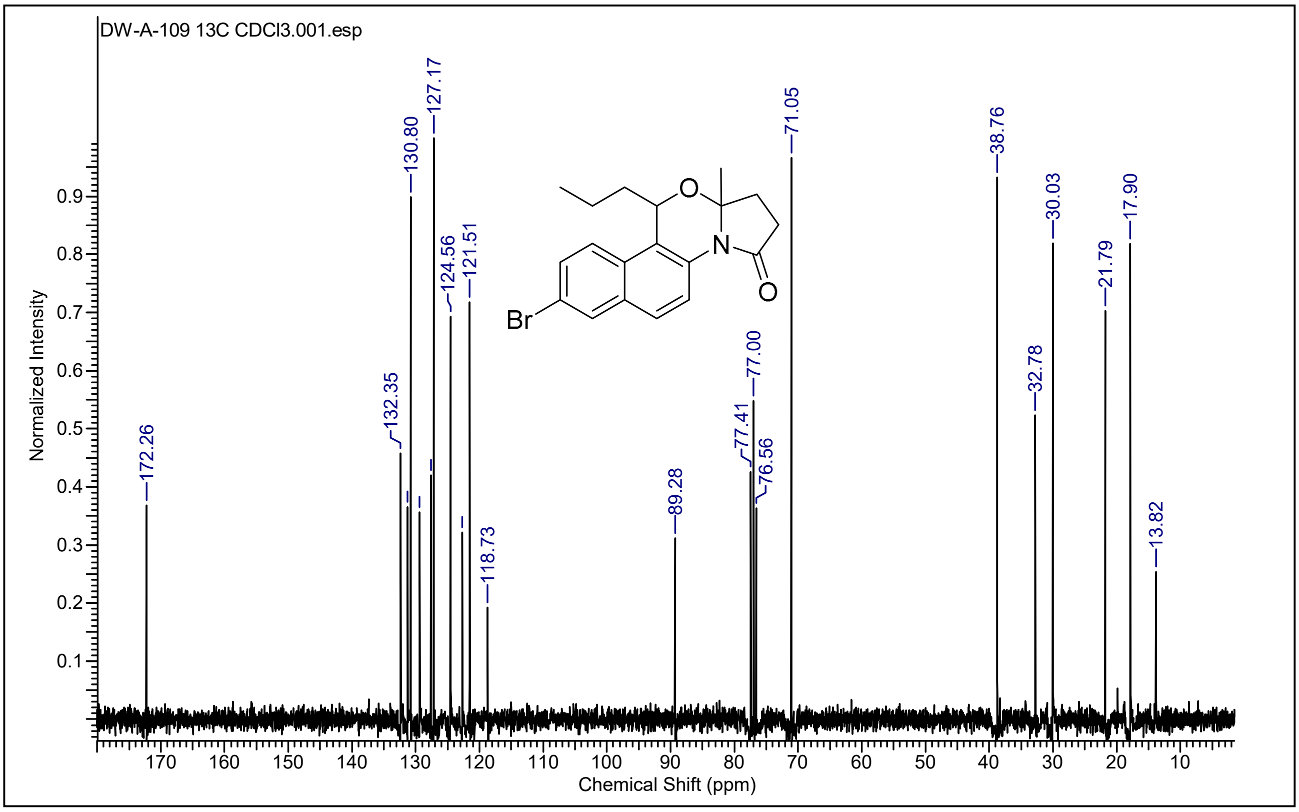


$^{1}$H and $^{13}$C NMR spectra of compound **4h**

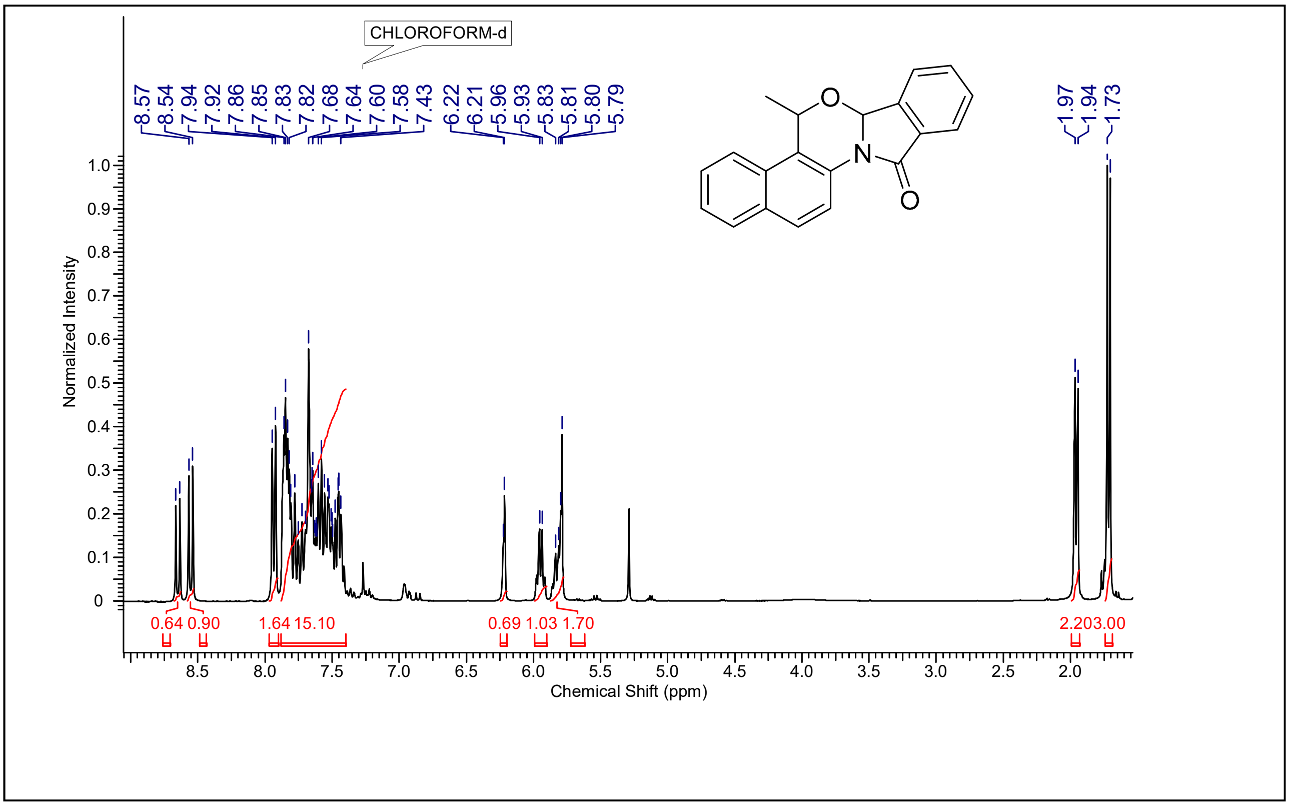


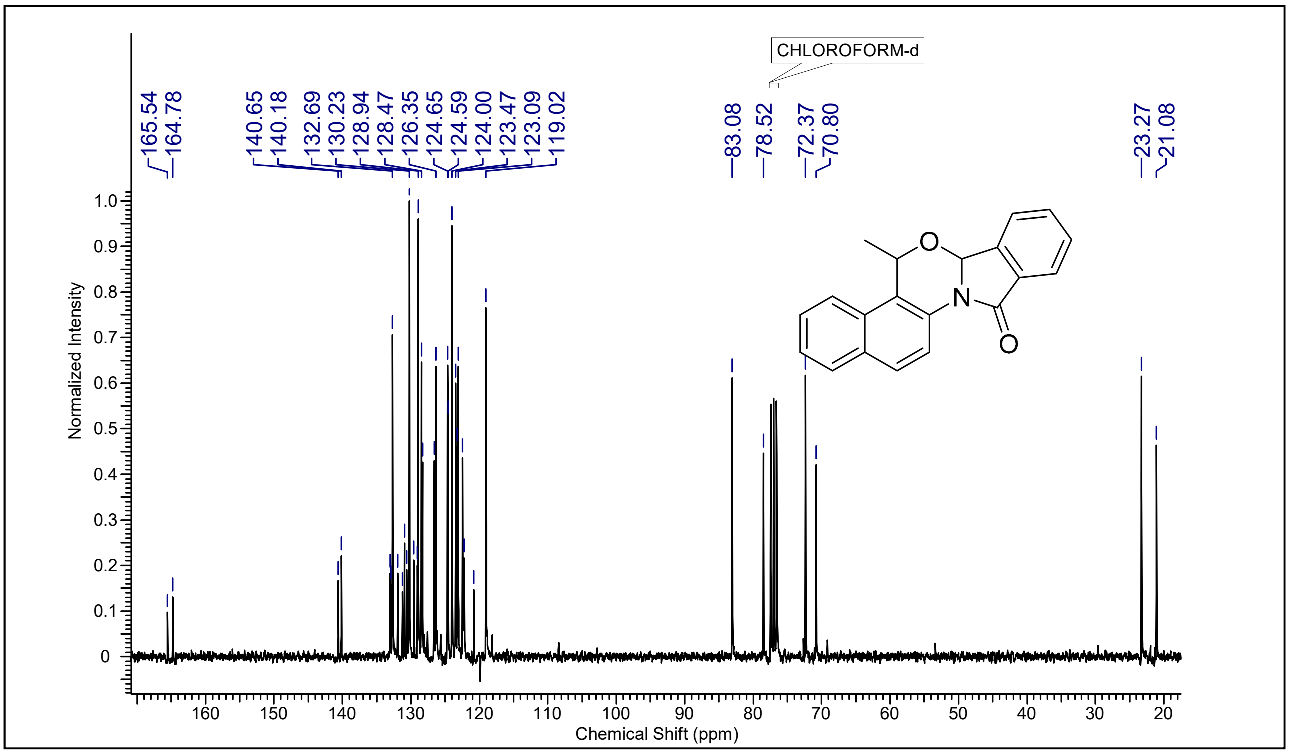


$^{1}$H and $^{13}$C NMR spectra of compound **4i**

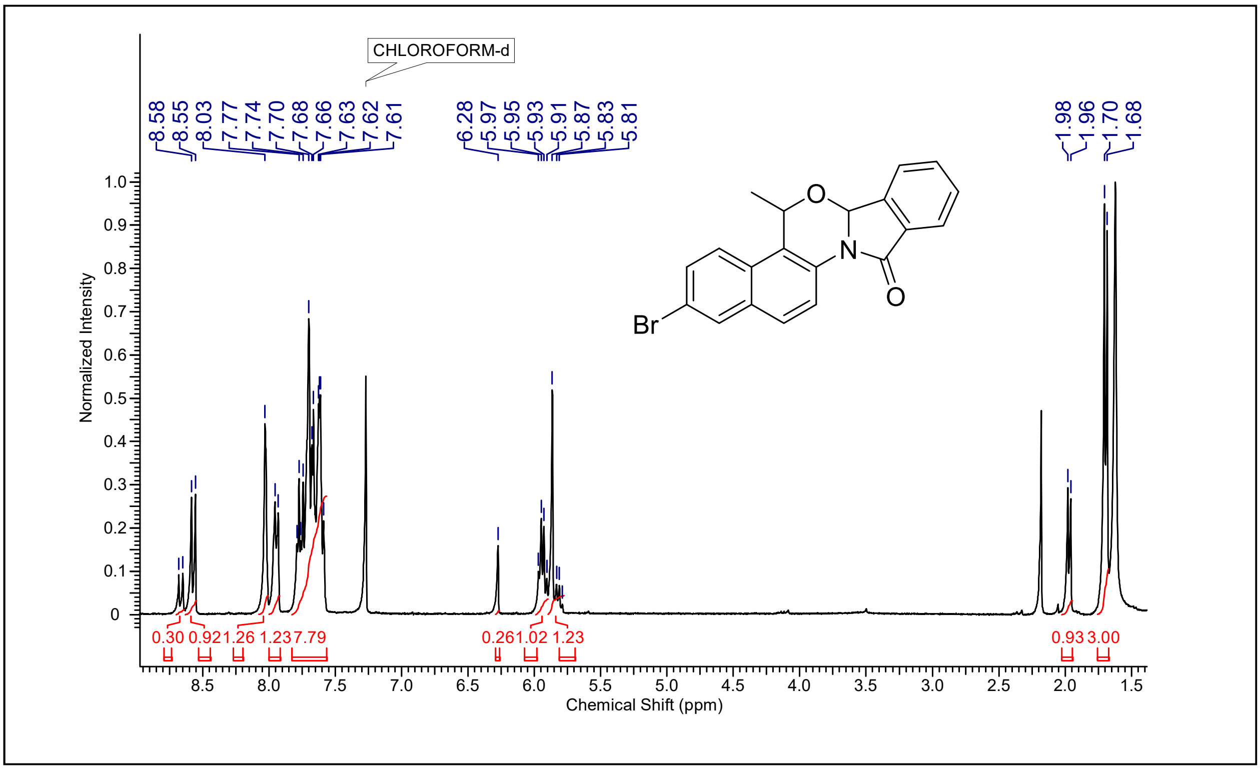


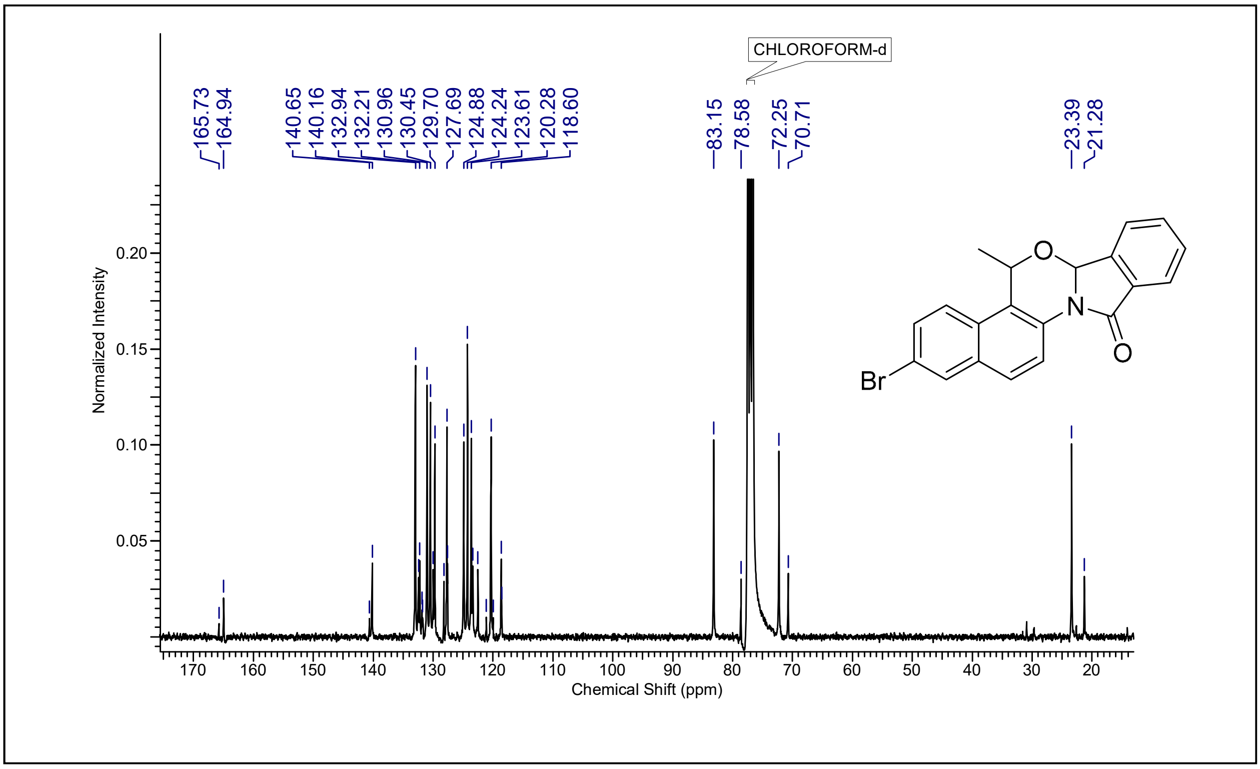


$^{1}$H and $^{13}$C NMR spectra of compound **4j**

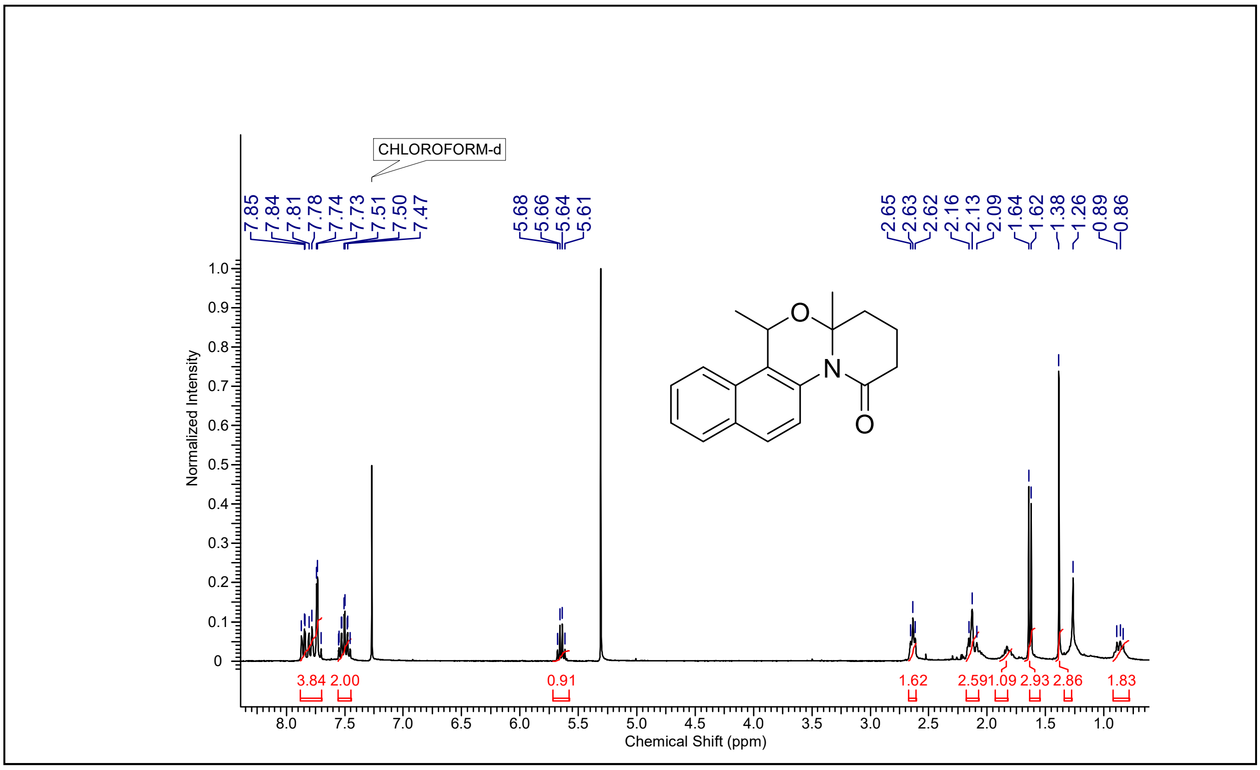


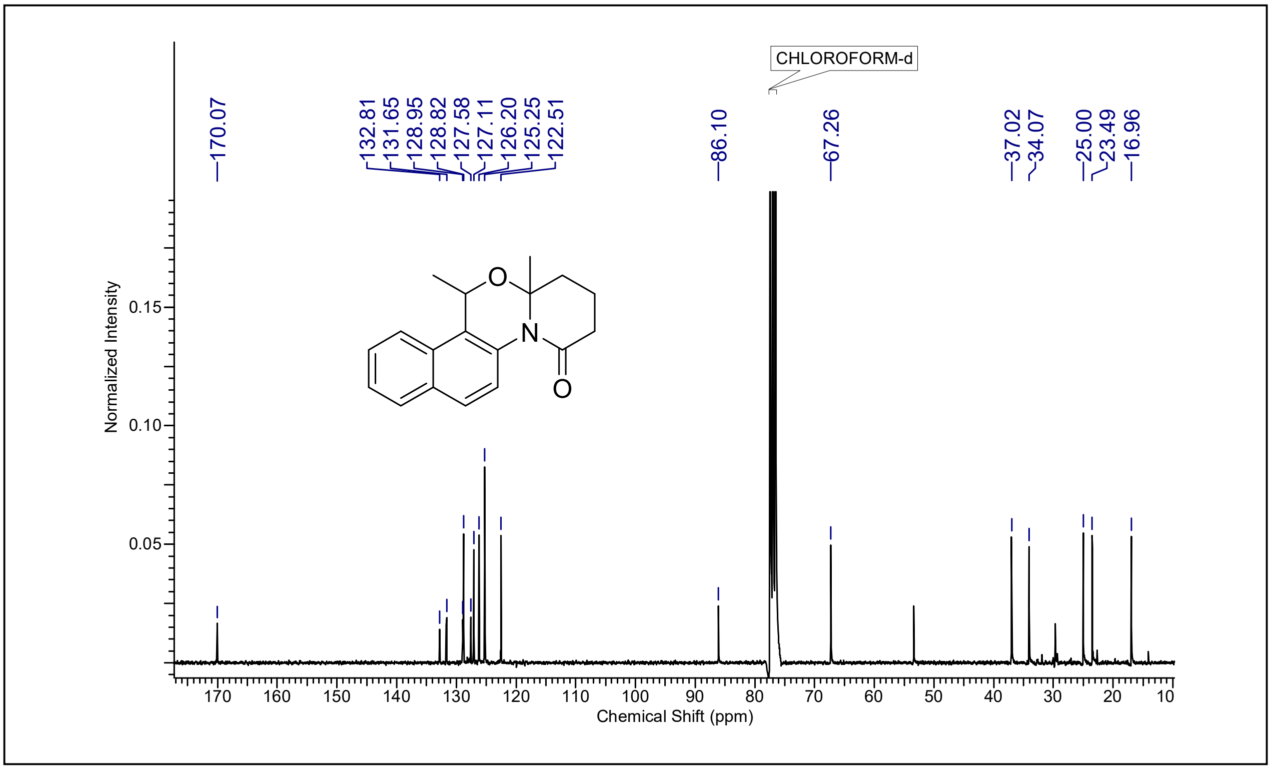


$^{1}$H and $^{13}$C NMR spectra of compound **4k**

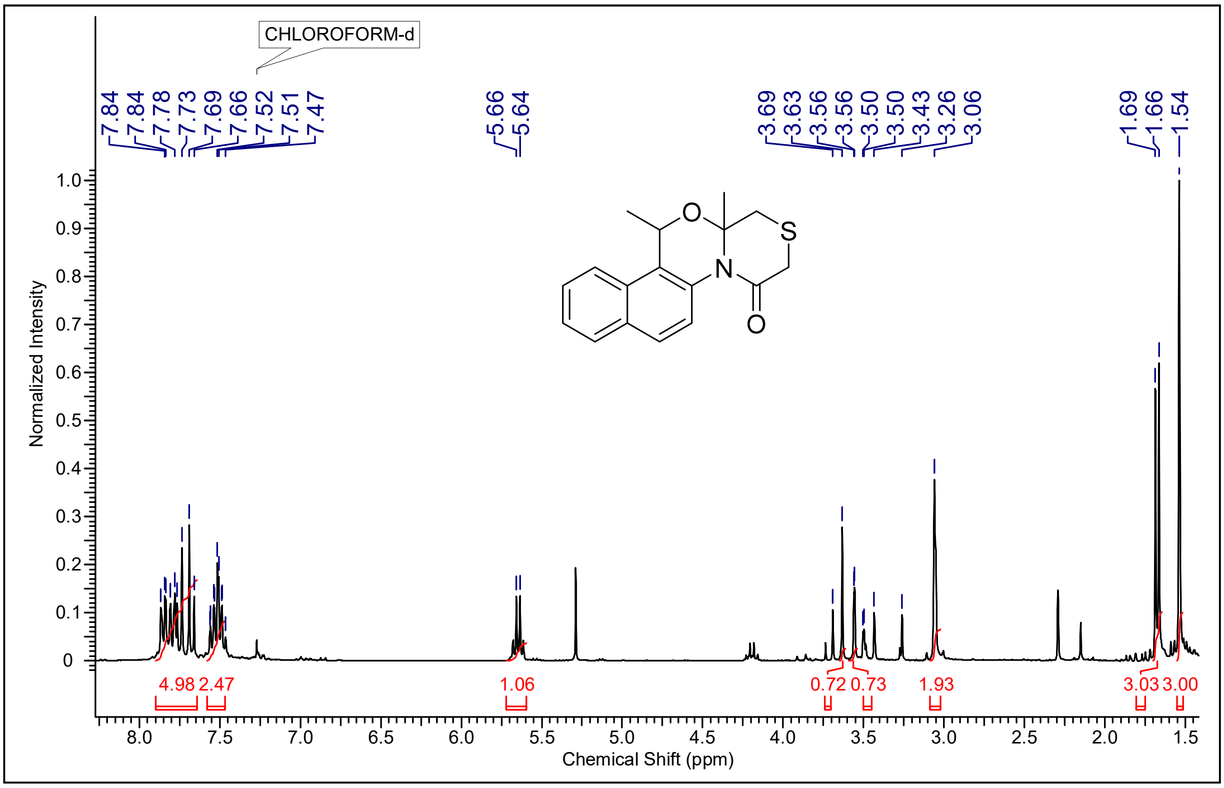


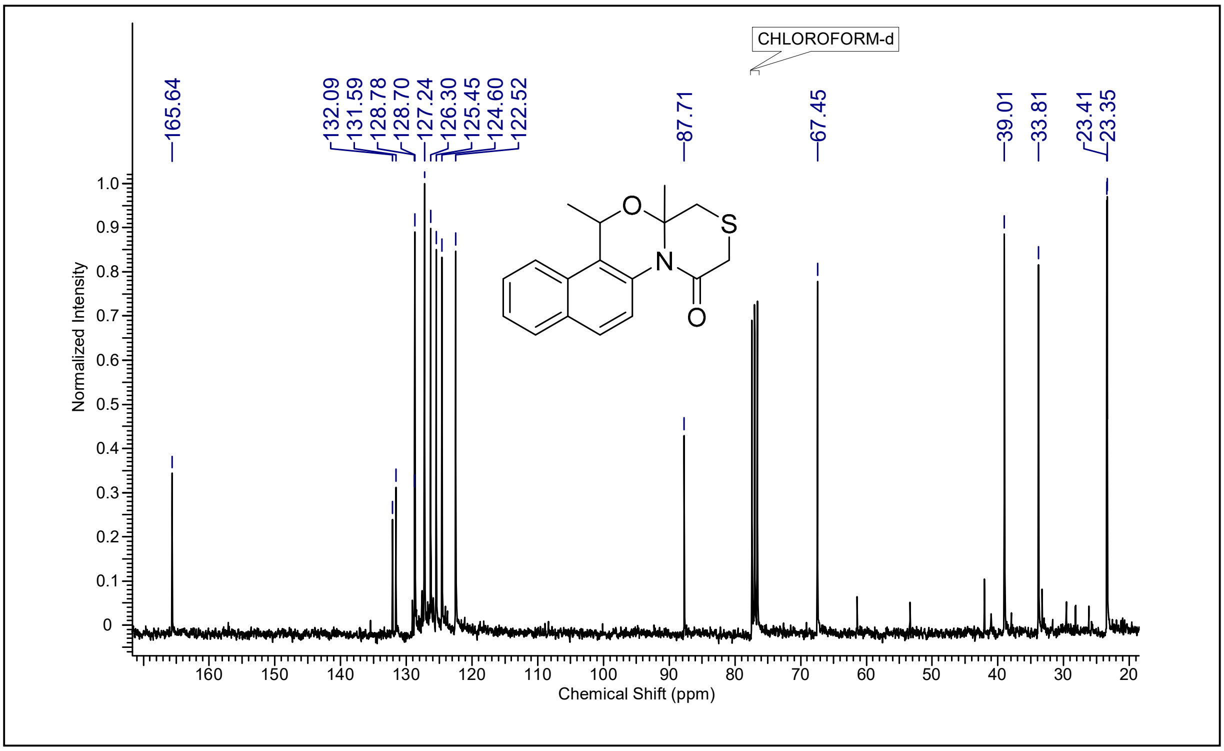


$^{1}$H and $^{13}$C NMR spectra of compound **4l**

**X-ray Crystal Structure of Compound 4b (CCDC-2006680)**

The crystal chosen was attached to the tip of a MicroLoop with Paratone-N oil. Measurements were made on a Bruker D8 VENTURE diffractometer equipped with a PHOTON III CMOS detector using monochromated Cu Kα radiation (λ = 1.54178 Å) from an Incoatec micro-focus sealed tube at 100 K [1]. The initial orientation and unit cell were indexed using a least-squares analysis of the reflections collected from a complete 360° phi-scan, 2 seconds per frame and 1° per frame. For data collection, a strategy was calculated to maximize data completeness and multiplicity, in a reasonable amount of time, and then implemented using the Bruker Apex 3 software suite [1]. The crystal to detector distance was set to 4.0 cm. Cell refinement and data reduction were performed with the Bruker SAINT [2] software, which corrects for beam inhomogeneity, possible crystal decay, Lorentz and polarization effects. A multi-scan absorption correction was applied (SADABS [3]). The structure was solved using SHELXT-2014 [4-5] and was refined using a full-matrix least-squares method on $F^2$ with SHELXL-2018 [4-5]. The non-hydrogen atoms were refined anisotropically. The hydrogen atoms bonded to carbon were included at geometrically idealized positions and were not refined. The isotropic thermal parameters of these hydrogen atoms were fixed at $1.2U_{eq}$ of the parent carbon atom or $1.5U_{eq}$ for methyl hydrogens.

The structure was found to crystallize in the polar space group $Pna2_1$, with two molecules in the asymmetric unit. Molecule one was found to have S chirality at C4 and R chirality at C6. Similarly, molecule two was found to have S chirality at C26 and R chirality at C28. The absolute structure of the molecule was reliably determined; although values did not refine completely to zero as expected, they are close enough to be taken as such. Using the program Platon [6] the refined structure was calculated to have a Flack parameter of 0.10(3), a Parsons parameter of 0.08(3) and a Hooft parameter of 0.08(3). These values agree with the Parson's value calculated by the program SHELXL, 0.098(32) from 3435 selected quotients.

CCDC-2006680 contains the supplementary crystallographic data for this paper. These data can be obtained free of charge via http://www.ccdc.cam.ac.uk/conts/retrieving.html (or from the CCDC, 12 Union Road, Cambridge CB2 1EZ, UK; Fax: +44 1223 336033; E-mail: deposit@ccdc.cam.ac.uk.

Table S1. Crystal data and structure refinement for Compound **4b**.

| | | |
|---|---|---|
| Identification code | **4b** | |
| CCDC deposit number | 2006680 | |
| Empirical formula | $C_{22}H_{19}NO_2$ | |
| Formula weight | 329.38 | |
| Temperature | 100(2) K | |
| Wavelength | 1.54178 Å | |
| Crystal system | Orthorhombic | |
| Space group | $Pna2_1$ | |
| Unit cell dimensions | $a$ = 11.3771(10) Å | α = 90° |
| | $b$ = 8.9425(6) Å | β = 90° |
| | $c$ = 33.105(3) Å | γ = 90° |
| Volume | 3368.1(5) $Å^3$ | |
| $Z$ | 8 | |
| Density (calculated) | 1.299 $Mg/m^3$ | |
| Absorption coefficient | 0.658 $mm^{-1}$ | |
| F(000) | 1392 | |
| Crystal size | 0.287 x 0.154 x 0.059 $mm^3$ | |
| Theta range for data collection | 2.669 to 79.547° | |
| Index ranges | -14<=h<=13, -11<=k<=11, -41<=l<=42 | |
| Reflections collected | 68387 | |
| Independent reflections | 7252 [R(int) = 0.0264] | |
| Completeness to theta = 67.679° | 100.0 % | |
| Absorption correction | Semi-empirical from equivalents | |
| Max. and min. transmission | 0.7543 and 0.6861 | |
| Refinement method | Full-matrix least-squares on $F^2$ | |
| Data / restraints / parameters | 7252 / 1 / 453 | |
| Goodness-of-fit on $F^2$ | 1.048 | |
| Final R indices [I>2sigma(I)] | R1 = 0.0283, wR2 = 0.0743 | |
| R indices (all data) | R1 = 0.0287, wR2 = 0.0747 | |
| Absolute structure parameter | 0.10(3) | |
| Largest diff. peak and hole | 0.228 and -0.213 e.$Å^{-3}$ | |

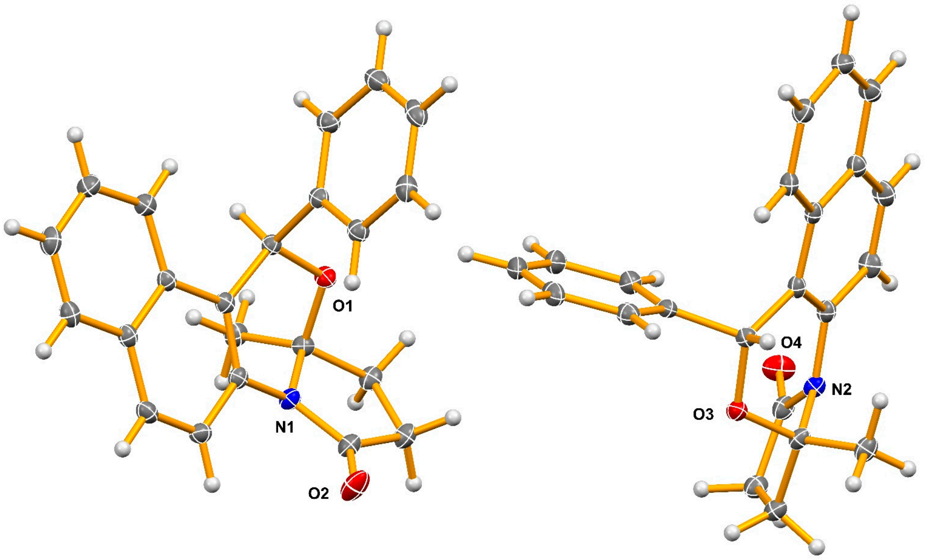


**Figure S1.** Structure of compound **4b** showing the two molecules of the independent unit in the correct relative orientation. Only the non-carbon atoms have been labelled. Thermal ellipsoids have been drawn at the 50% probability level. Hydrogen atoms are included but have not been labelled.

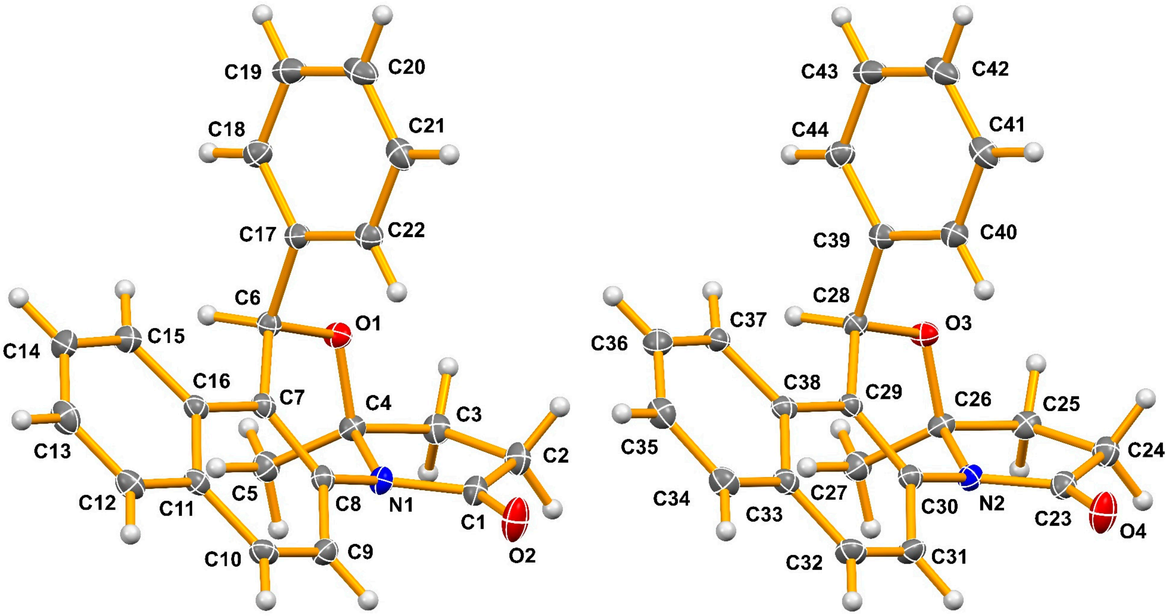


**Figure S2.** Separate diagrams of the two molecules in compound **4b** showing the full heavy atom labelling. Thermal ellipsoids have been drawn at the 50% probability level. Hydrogen atoms are included but have not been labelled.

**References**


[1] APEX 3 (Bruker, 2018) Bruker AXS Inc., Madison, Wisconsin, USA.
[2] SAINT (Bruker, 2016) Bruker AXS Inc., Madison, Wisconsin, USA.
[3] SADABS (Bruker, 2016) Bruker AXS Inc., Madison, Wisconsin, USA.
[4] Sheldrick, G.M. SHELXT – Integrated space-group and crystal structure determination. Acta *Cryst*. **2015**, A*71*, 3-8.
[5] Sheldrick, G.M. Crystal structure refinement with SHELXL. *Acta Cryst*. **2015**, *C71*, 3-8.
[6] Spek, A.L. Structure validation in chemical crystallography, *Acta Cryst*. **2009**, *D65*, 148- 155.
